# Differential protein expression and post-translational modifications in metronidazole-resistant *Giardia duodenalis*

**DOI:** 10.1093/gigascience/giy024

**Published:** 2018-03-13

**Authors:** Samantha J Emery, Louise Baker, Brendan R E Ansell, Mehdi Mirzaei, Paul A Haynes, Malcom J McConville, Staffan G Svärd, Aaron R Jex

**Affiliations:** 1 Population Health and Immunity Division, Walter and Eliza Hall Institute of Medical Research, Melbourne, VIC, Australia; 2 Faculty of Veterinary and Agricultural Sciences, The University of Melbourne, Melbourne, VIC, Australia; 3 Chemistry and Biomolecular Sciences, Faculty of Science, Macquarie University, North Ryde, NSW, Australia; 4 Australian Proteome Analysis Facility, Macquarie University, North Ryde, NSW, Australia; 5 Bio21 Molecular Science and Biotechnology Institute, The University of Melbourne, Melbourne, VIC, Australia; 6 Department of Cell and Molecular Biology, Uppsala University, Uppsala, Sweden

**Keywords:** *Giardia duodenalis*, quantitative proteomics, metronidazole, drug resistance, protein post-translational modifications

## Abstract

**Background:**

Metronidazole (Mtz) is the frontline drug treatment for multiple anaerobic pathogens, including the gastrointestinal protist, *Giardia duodenalis*. However, treatment failure is common and linked to *in vivo* drug resistance. In *Giardia, in vitro* drug-resistant lines allow controlled experimental interrogation of resistance mechanisms in isogenic cultures. However, resistance-associated changes are inconsistent between lines, phenotypic data are incomplete, and resistance is rarely genetically fixed, highlighted by reversion to sensitivity after drug selection ceases or via passage through the life cycle. Comprehensive quantitative approaches are required to resolve isolate variability, fully define Mtz resistance phenotypes, and explore the role of post-translational modifications therein.

**Findings:**

We performed quantitative proteomics to describe differentially expressed proteins in 3 seminal Mtz-resistant lines compared to their isogenic, Mtz-susceptible, parental line. We also probed changes in post-translational modifications including protein acetylation, methylation, ubiquitination, and phosphorylation via immunoblotting. We quantified more than 1,000 proteins in each genotype, recording substantial genotypic variation in differentially expressed proteins between isotypes. Our data confirm substantial changes in the antioxidant network, glycolysis, and electron transport and indicate links between protein acetylation and Mtz resistance, including cross-resistance to deacetylase inhibitor trichostatin A in Mtz-resistant lines. Finally, we performed the first controlled, longitudinal study of Mtz resistance stability, monitoring lines after cessation of drug selection, revealing isolate-dependent phenotypic plasticity.

**Conclusions:**

Our data demonstrate understanding that Mtz resistance must be broadened to post-transcriptional and post-translational responses and that Mtz resistance is polygenic, driven by isolate-dependent variation, and is correlated with changes in protein acetylation networks.

## Background

Nitroheterocyclics (e.g., metronidazole [Mtz], nitazoxanide, and furazolidone) include “redox-active” pro-drugs that cross the cell membrane via passive diffusion and are enzymatically reduced to cytotoxic intermediates that oxidize biomolecules. This occurs specifically within highly reducing intracellular environments of microaerophilic protists (*Giardia duodenalis, Trichomononas vaginalis*, and *Entameoba histolytica*) as well as anaerobic bacteria (*Helicobacter pylori, Clostridium difficile*, and *Bacteroides fragilis*) [1, 2]. By contrast, Mtz has low toxicity in aerobic cells, where dO_2_ re-oxidizes reduced Mtz to the pro-drug form, termed “futile cycling.” The specificity of nitroheterocyclic toxicity for low dissolved oxygen biochemistry makes this drug class the chemotherapeutic backbone against multiple bacterial and protozoan pathogens. However, drug resistance within this compound class is collectively widespread across species [2–4].

*Giardia duodenalis* (syn. *G. lamblia, G. intestinalis*) is a parasitic protist responsible for between 200 and 300 million cases of diarrheal disease (giardiasis) annually [5]. This microaerophile exhibits fermentative glycolysis coupled to an antioxidant system that maintains an electron-rich (i.e., highly reduced) intracellular environment. Chemotherapeutic treatments for giardiasis are limited but remain the primary treatment option, targeting the trophozoite, which is the infective life stage that attaches to the gastro-epithelial lining of the proximal small intestine [6]. Nitroheterocyclics, in particular Mtz, remain the predominant class against these parasites. However, the efficacy of frontline Mtz treatment ranges from 73 to 100% in *Giardia* [7], with clinical resistance confirmed [7, 8] and increasing in incidence [9].

Mtz interacts with oxidoreductase enzymes in *Giardia*, which include glycolytic and antioxidant enzymes, but is also influenced by enzymes that contribute to the reduction potentials through electron transport, cofactor abundance, and flavin metabolism. Changed activity or expression of these enzymes correlates with resistance, with changes leading to decreased activation or increased detoxification of Mtz, known as passive and active resistance mechanisms, respectively [2]. In *Giardia*, the downregulation or reduced activity of pyruvate:ferredoxin oxidoreductase (PFOR) is a centrally recognized passive resistance mechanism [10–14]. However, transcriptional studies suggest that exposure to Mtz elicits genome-wide changes in *Giardia* [15], including within wider glycolytic and redox systems. Notably, downregulation of thioredoxin reductase [10], which links thiol metabolism to thioredoxins and peroxiredoxins in the antioxidant system, is a passive resistance mechanism that can limit activation of Mtz, albeit at presumed costs to collateral antioxidant systems. Furthermore, the role of the 2 nitroreductases (NRs) in *Giardia* have been implicated in Mtz resistance, with NR-1 and NR-2 activating and detoxifying MtzR, respectively, and are active (NR-2) and passive (NR-1) resistance mechanisms. NR-1 transcript levels are reduced in Mtz-resistant lines [16–18], and the enzyme is increasingly recognized as a PFOR-independent mechanism of passive resistance. Drug-resistant lines also exhibit differential transcription protein chaperones, thiol-cycling, and stress response genes [16], as well as DNA repair mechanism transcriptional regulators [19, 20].

Collectively, evidence suggests that Mtz resistance is a complex polygenic phenotype (reviewed by Ansell et al. [2]). Namely, divergent changes in transcript abundance between genetically similar Mtz-resistant *Giardia* [10, 19] and laboratory lines [15, 18] suggest multiple Mtz-resistant molecular phenotypes. Further, the interactions of transcriptional expression, enzyme activity, and, recently, nonsynonymous mutations [18] remain to be understood in key enzymes. Phenotypic aspects including infectivity and fitness also differ in lines of different genetic background selected for Mtz resistance *in vivo* and *in vitro* [14]. Plasticity in the resistance phenotype during encystation [19] or when drug selection is discontinued [21] further suggests reversible or inducible transcriptional regulation. Transcriptional plasticity has been linked to Sir2 nicotinamide adenine dinucleotide (NAD)-dependent protein deacetylases (sirtuins) [2, 19] and may indicate a role for reversible protein modifications in resistance phenotypes. RNA transcription and control of gene expression in *Giardia* [22–24] suggest an important role for post-transcriptional and post-translation regulation, and a global description of protein expression is a key, missing link in Mtz-resistance research. In light of this, we undertook detailed, quantitative proteomic analyses in Mtz-resistant and -susceptible *Giardia* lines to identify differentially expressed proteins. To our knowledge, this marks the first such analysis of Mtz resistance in any parasitic pathogen. This work was conducted in 3 genetically distinct *Giardia* cell culture isolates that each have been heavily characterized in the literature [25–27] and have shaped the foundational understanding of Mtz resistance in the genus [10, 14, 28]. Moreover, we examined dynamic changes in a wide range of post-translational protein modifications in Mtz-resistant and -susceptible and isogenic isolates and, in the latter, after several months of drug-free passage.

## Data Description

*Giardia duodenalis* Mtz-resistant (MtzR) and Mtz-susceptible (MtzS) lines were previously generated at the Queensland Institute of Medical Research via long-term sublethal exposure to Mtz in *in vitro* culture. All lines are the Assemblage A genotype, include the genome reference genotype WB (American Type Culture Collection [ATCC] 50803), and have been extensively characterized in the literature in the context of Mtz resistance (reviewed by Ansell et al. [2]. *In vitro* culture for the 3 genotypes and drug selection for their resistant, isotype lines (Table 1) were continued in this study; protein was extracted from adherent, viable trophozoites. Protein was prepared for quantitative proteomics via tandem mass tag (TMT) isobaric labeling to establish fold change between each MtzR line compared to their drug-susceptible parent line. A total of 3 TMT experiments were performed, 1 for each genotype and its respective isotype and biological triplicates. Each TMT experiment and its fractions were analyzed using high-resolution mass spectrometry on a Q-Exactive mass spectrometer (Thermo). Ratios of TMT labels detected in each Mtz-resistant to Mtz-susceptible replicates were calculated using Proteome Discoverer software v1.3 (Thermo). The mass spectrometry raw data files, database search results, and TMT ratios have been deposited and can be accessed for free via the ProteomeXchange Consortium [29] via the PRIDE (PRoteomics IDEntifications) partner repository with the dataset identifier PXD007183.

**Table 1:** IC_50_ and resistance factor for metronidazole in the 3 isogenic isolates used in this study

					Resistance
Isolate	Strain	Abbreviation	Reference	Mtz IC_50_	factor
WB	WB1B	WB-MtzS	[26]	8.28 μM	-
	WB1B-M3	WB-MtzR	[31]	22.79 μM	2.8
106	106	106-MtzS	[101]	9.39 μM	-
	106-2ID10	106-MtzR	[30]	23.99 μM	2.6
713	713	713-MtzS	[26]	7.79 μM	-
	713-M3	713-MtzR	[31]	42.33 μM	5.4

Divergence between differentially expressed proteins in MtzR isotypes led us to consider the potential for genotypic variation, transcriptional plasticity, and reversible protein modifications in Mtz resistance. Immunoblotting was performed for acetylation, methylation, ubiquitination, and phosphorylation for the 3 genotypes and isotype lines, as well as for isotype lines monitored after cessation of drug selection every 4 weeks for up to 12 weeks. Isotype lines maintained without Mtz were also monitored for reversion to Mtz sensitivity, and IC_50_ was calculated at 4, 8, and 12 weeks after cessation of drug selection to observe resistance phenotype stability. Chemical inhibitors of protein acetylation, methylation, and phosphorylation were also used to further probe post-translational responses, with the IC_50_ calculated for post-translational modification (PTM) inhibitors in the 6 lines, which was further complemented by immunoblotting of protein extracted from trophozoites exposed to chemical inhibitors.

## Results

### Cell culture of MtzR lines

All 3 MtzR lines were generated at similar times at the Queensland Institute of Medical Research via long-term exposure to sublethal Mtz [30, 31], with intermittent drug treatment and ultraviolet radiation (WB and 713) also used to further induce resistance [31]. WB-M3, BRIS/83/HEPU/106-2ID10, and BRIS/83/HEPU/713-M3 were further explored in subsequent studies [2, 10–12, 14, 16, 32, 33]. The resistant lines used in the current study were selected in the presence of 30 μM Mtz [19] and exhibited significantly higher Mtz IC_50_ values than their respective parent lines, indicating increased drug tolerance (Table 1).

### Quantitative proteomics

The complete datasets for each isogenic pair, including protein identification and quantitation results as well as label ratios and statistical test results, can be found in Supplementary Data 1. Peptide-to-spectrum matching was performed for all 3 isolates using the A1 subassemblage genome (WB C6, ATCC 50 803). This reference has few single-nucleotide polymorphisms relative to other sequences to date [34] and has been previously used as a database for proteomic analysis of all 3 isolates with no significant differences in peptide identifications [35]. A nonredundant total of 1,571 proteins was identified across the TMT 10plexes analyzed, with 1,220, 1,126, and 1,060 proteins identified in 10plex 1–3, respectively (Table 2).

**Table 2:** Summary of protein identifications and differentially expressed proteins in the three TMT 10 plexes

	TMT1 (WB-MTZR V MTZS)	TMT2 (106-MTZR V MTZS)	TMT2 (713-MTZR V MTZS)
No. of protein IDs	1220	1126	1060
No. of nonredundant peptides	9684	8692	6349
No. of differentially expressed proteins	264	171	76
No. of upregulated proteins	128	87	39
No. of downregulated proteins	137	84	37

Proteins were considered differentially expressed if proteins were statistically significant (*P* value ≤ 0.05) and met ratio fold change cutoffs for upregulation (≥1.3) or downregulation (≤0.77).

To quantify protein abundance between drug-resistant (MtzR) and drug-susceptible (MtzS) isolates, 9 ratios were calculated with each MtzR replicate over all 3 MtzS replicates, and the geometric mean was calculated as a measure of fold change. Reporter ion intensity for each protein was calculated using the pooled control as a common denominator for each TMT channel for normalization and was analyzed statistically via a 1-sample *t* test between treatments. Differential expression was contingent on proteins meeting both fold change and *P* value cutoffs, as previously described for isobaric label quantitation [36, 37] and as depicted in Supplementary Fig. S1, panel A. Principal component analysis (PCA) of each of the 3 10plexes indicated good separation between MtzS parent isolates and their isogenic MtzR lines (Supplementary Fig. S1, panel B), with control MtzS replicates clustering together tightly in all 3 isolates. Clustering of MtzR lines in the PCA indicated that all 3 MtzR lines were more variable between replicates than MtzS isogenic parents. Nonetheless, analysis of *P* value distribution revealed an inverse exponential distribution (Supplementary Fig. S1, panel C), consistent with the existence of an underlying signal of differential expression between MtzS and MtzR populations in all 3 isolates [38].

### Differentially expressed proteins in MtzR lines

**Figure 1:**
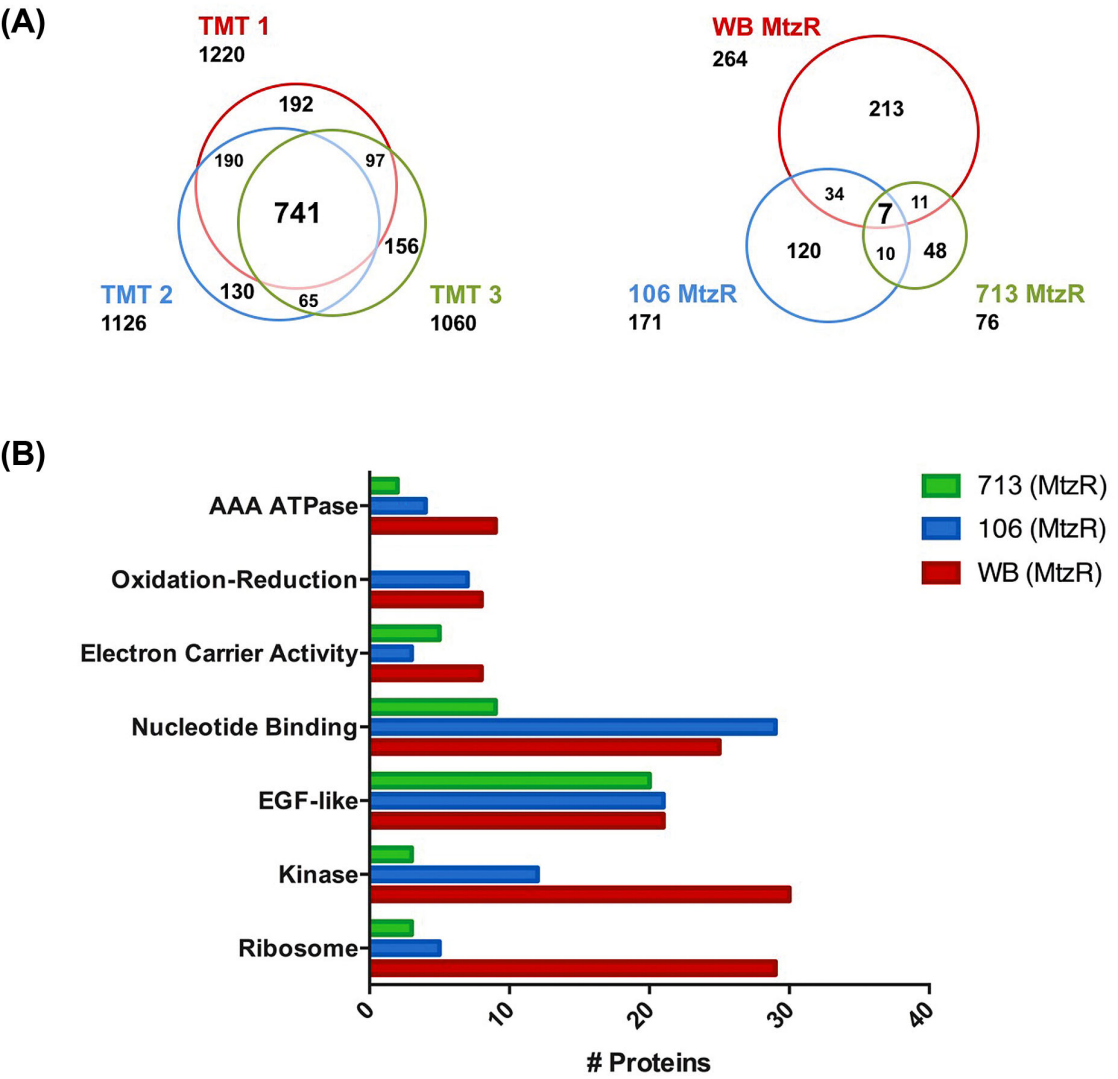
Protein identification, differential expression, and functional enrichment in Mtz lines. Proportional Venn diagrams showing unique and overlapping protein identifications in the 3 TMT 10-plexes (left) and for differentially expressed proteins in each MtzR line compared to MtzS parents (right). (B) The 6 functional clusters identified as enriched from differentially expressed proteins identified in MtzR isolates and their total protein number.

A nonredundant total of 443 proteins met both fold change and *P* value thresholds for differential expression in the 3 MtzR isolate lines. Correlations between protein and RNA fold changes [18] from the same cell-pellet material in MtzR lines compared to MtzS were calculated at r^2^ = 0.154 for WB, r^2^ = 0.105 for WB, and r^2^ = 0.187 for WB (*P* < 0.01) for genes identified in both datasets (Supplementary Fig. S2). The largest number of differentially expressed proteins (DEPs) was identified in WB-MtzR with 264 DEPs, followed by 106-MtzR (171 DEPs) and 713-MtzR (76 DEPs). The proportions of upregulated and downregulated proteins were approximately equal in each resistant line (Table 2). Of the nonredundant 443 DEPs, only 7 (1.6%) were common between all 3 MtzR lines (Fig. 1, panel A). A further 55 DEPs (12.5%) were variously detected in 2 of the 3 lines. Overall, the majority of DEPs (86.4%) were unique to each line. This distinction between DEPs was not due to discrepancies in protein identifications between TMT 10plex experiments, as in each of the 3 10plexes, 741 proteins were common identifications, constituting between 60.7% and 69.9% of proteins identified in each and 47.2% of the nonredundant total (Fig. 1, panel A). Furthermore, the majority of DEPs in each MtzR line were identified in the other experiments (Supplementary Fig. S3).

Of the 7 DEPs common between MtzR lines, there were 6 functionally annotated proteins, including 2 variant-specific surface proteins (VSPs) (137 620, 37 093), 1 membrane-associated cysteine-rich endopeptidase (14 225), 1 epidermal growth factor (EGF)-like transmembrane protein, and 2 proteins with oxidoreductase activity, glutamate synthase (7195) and NR-1 (6175), the latter of which was significantly downregulated in all MtzR lines and is consistent with its role in Mtz activation and resistance [16, 19, 39].

### Gene set enrichment analysis for differentially expressed proteins

The 264, 126, and 76 total DEPs in WB-MtzR, 106-MtzR, and 713-MtzR (relative to their isogenic susceptible parental control), respectively, were DAVID for functional clustering analysis. A nonredundant total of 6 functional clusters (Supplementary Table S3) were identified as enriched among DEPs across the 3 MtzR isolates (Fig. 1, panel B), including “AAA ATPase,” 2 “Electron Carrier Activity” clusters, and “EGF-like,” “Kinase,” and “Ribosome” clusters, with the “EGF-like” cluster the most consistent in terms of gene families and numbers between MtzR lines. Overall, although DEP identifications and their directionality diverged between MtzR isolates, there was some convergence between MtzR lines at the level of protein function, with different members of the same gene or functional families detected among differentially regulated clusters of DEPs for each isolate. Functional annotation including gene ontology (GO) and Interpro annotations for DEPs can be found in Supplementary Table S2. Some of the clusters identified in the GSEA were mirrored in results from STRING (Search Tool for the Retrieval of Interacting Genes) software, in particular, protein–protein interactions surrounding ribosomal function, ABC transporters, antioxidant and glycolysis, and phosphorylation (Supplementary Fig. S4).

The “Ribosome” term assigned to ribosome structural constituents and related proteins was specifically enriched within WB-MtzR and reflected in its STRING interaction network (Supplementary Fig. S4), with 29 DEPs compared to 5 and 3 in 106-MtzR and 713-MtzR, respectively. A total of 24 structural constituents of ribosomes were downregulated in WB-MtzR, which coincides with transcriptional data; transcripts of multiple ribosomal and ribosome-associated proteins as downregulated in WB-MtzR were also observed [18]. A set of P-loop containing nucleotide hydrolases (IPR027417), particularly AAA+/AAA-type ATPase domains (IPR003593/IPR003959), were enriched among DEPs in 3 isolates, albeit with differences in directionality of expression, with 17, 11, and 7 DEPS in WB-MtzR, 106-MtzR, and 713-MtzR, respectively. This gene set featured 2 main classes of proteins, including DNA/nucleic acid binding proteins, and transmembrane ABC transporters, which are frequently associated with the membrane translocation of toxic compounds, including drug compounds [40]. These ABC transporters were also detected as interaction partners through STRING interaction networks of DEPs in the 3 isolates (Supplementary Fig. S4). In WB-MtzR, 3 ABC transporters were upregulated (113 876, 28 379), including 1 that was also upregulated in 713-MtzR (16 592), which is specifically involved in lipid transport (GO:0 006869). However, in 106-MtzR, the 3 differentially expressed ABC transporters were downregulated (17 132, 38 104), including 1 also downregulated in 713-MtzR (115 052). Several proteins within this set were functionally related to transcriptional regulation, 3 of which were upregulated in WB-MtzR (89 112, 8228, 2098) and differentially expressed in 106-MtzR (112 978, 8228). Further, the MAD-2 mitotic regulator was downregulated in 713-MtzR compared to universal upregulation between isolates at the transcript level [18].

### EGF-like proteins and VSPs

**Figure 2:**
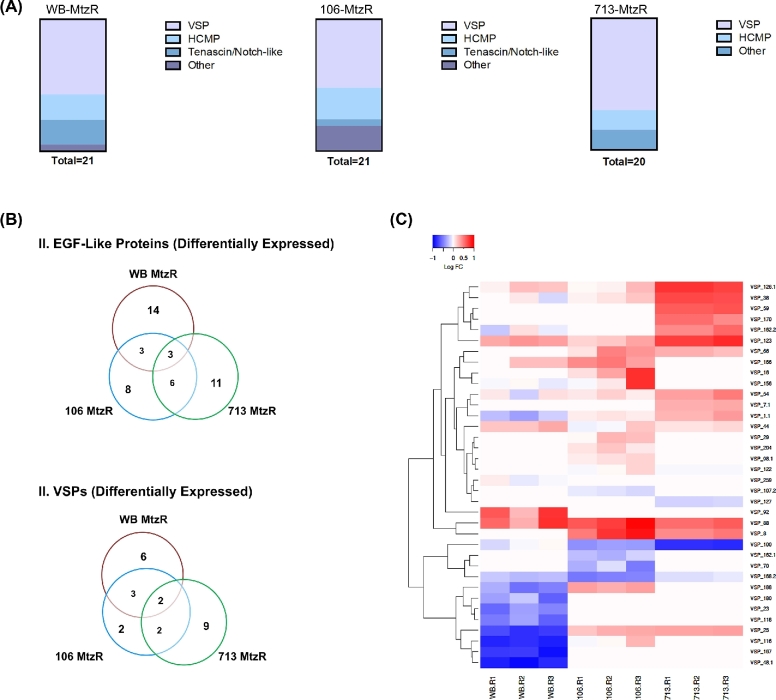
EGF-like differentially expressed proteins and VSP subpopulations. (A) Distribution of differentially expressed EGF-like proteins within *Giardia* protein families by MtzR line. (B) Proportional Venn diagrams showing overlapping identities of all differentially expressed EGF-like proteins in MtzR lines (above) and all differentially expressed VSP proteins (below). (C) Heat map showing fold change in expression of VSPs in MtzR lines compared to the MtzS parents; replicate details are shown on the bottom axis to represent biological variation within lines. Lower fold changes are represented by blue, while higher fold changes are represented in red.

The “EGF-like” gene set was enriched among DEPs for all 3 MtzR isolates (Fig. 2, panel A). This set included an abundance of VSPs, with high cysteine membrane proteins (HCMPs) as the second most pronounced group, particularly in 106-MtzR. The gene set also included EGF-like tenascin/notch-like proteins in WB-MtzR and 106-MtzR, which may be involved in signaling and have been observed to increase during *in vitro* host–parasite interactions [41, 42]. Several other EGF-like proteins were also present, including membrane-associated cysteine-rich endopeptidases in 106-MtzR and 713-MtzR. Although EGF-like proteins, particularly cysteine-rich families, were consistently differentially expressed in all MtzR isolates, only a few DEPs were common to the 3 MtzR lines (Fig. 2, panel B), with WB-MtzR and 713-MtzR most separated.

Overall, a nonredundant total of 36 VSPs (defined according to Adam et al. [43]) were identified in all 3 tenplexes, with fold change quantified in 23, 25, and 18 variants between MtzR and MtzS lines of WB, 106, and 713, respectively. The VSP gene family possessed the largest proportion of DEPs among *Giardia* gene families, with 47.8%, 36.0%, and 77.8% of VSPs differentially expressed in WB, 106, and 713-MtzR lines, respectively. Furthermore, the majority of differentially expressed VSPs were in the top 10 upregulated or downregulated proteins in terms of fold change, specifically, 9 of 11 VSPs in WB-MtzR were among the top DEGs, as were 7 of 9 in 106-MtzR and 9 of 13 in 713-MtzR. Although MtzR lines showed these similar trends in overall VSP differential expression, common specific VSP variants were limited between MtzR lines (Fig. 2, panel B), with only 2 common differentially expressed genes: VSP-123 (upregulated in all MtzR lines) and VSP-25 (upregulated in 106 and 713-MtzR and downregulated in WB-MtzR). Furthermore, MtzR lines also varied in directionality of differential expression (Fig. 2, panel C), with the majority of VSPs downregulated in WB-MtzR, upregulated in 713-MtzR, and dispersed between upregulated and downregulated in 106-MtzR. Overall, although the expressed VSP complement of MtzR lines differed from their parent MtzS lines, cluster analysis also revealed divergences between MtzR lines as well (Fig. 2, panel C).

### Oxidoreductase enzymes, PFOR, and pyruvate catabolism

**Figure 3:**
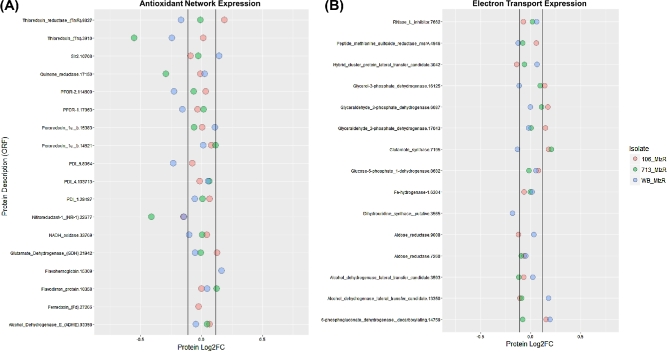
Protein expression in antioxidant and electron transport networks. Gene annotations including accession numbers (prefix “GL50803_”) and average protein expression fold change between MtzR from MtzS parents in (A) antioxidant proteins and (B) electron transport proteins.

The GO terms “electron carrier activity” (GO:0 009055) and “oxidation-reduction” (GO:00 55114) were enriched among DEPs in all 3 MtzR lines. In WB-MtzR, “iron-sulfur cluster” (GO:00 51536) and “iron ion binding” (GO:0 005506) annotation terms were also enriched. The overall expression of enzymes with oxidoreductase activity is shown in Fig. 3, depicting enzymes specifically implicated in the *Giardia* antioxidant network (Fig. 3, panel A) [2, 44] and others involved in electron transport and cofactor abundance (Fig. 3, panel B). Overall, although oxidoreductases were significantly enriched within differentially expressed proteins in all 3 MtzR lines, MtzR lines had largely divergent expression profiles, indicative of alternative mechanisms and pathways for either reduced activation of Mtz or mitigation of oxidative damage. Among all 3 lines, NR-1 (22 766) was universally significantly downregulated.

WB-MtzR displayed the most prominent downregulation trend of proteins in the antioxidant network (Fig. 3, panel A), with both PFOR proteins (114 609, 17 063) downregulated, as well as PDI5 (8064), thioredoxin reductase (9827), and a putative thioredoxin (3910). This indicates strong downregulation Mtz-activating genes in WB-MtzR and contrasts with upregulation of the transcripts encoding these proteins in 713-MtzR [18]. In contrast, 713-MtzR downregulated both a putative thioredoxin protein (3910) and a putative quinone reductase (17 150). Although transcriptomics previously indicated downregulation of quinone reductase as a universal mechanism of Mtz activation, the protein was not among DEPs in 106- and WB-MtzR. In 106-MtzR, NR-1 (22 766) was the only downregulated oxidoreductase enzymes. By contrast, thioredoxin reductase and glutamate dehydrogenase (21 942) were upregulated in 106-MtzR, both of which have been previously implicated in Mtz resistance but usually as being downregulated [2, 10, 18]. No antioxidant-related proteins were upregulated in 713-MtzR, while a Sir2 homologue (10 708) implicated in redox-mediated epigenetic regulation of transcription [2, 18, 45] was upregulated in WB-MtzR.

**Figure 4:**
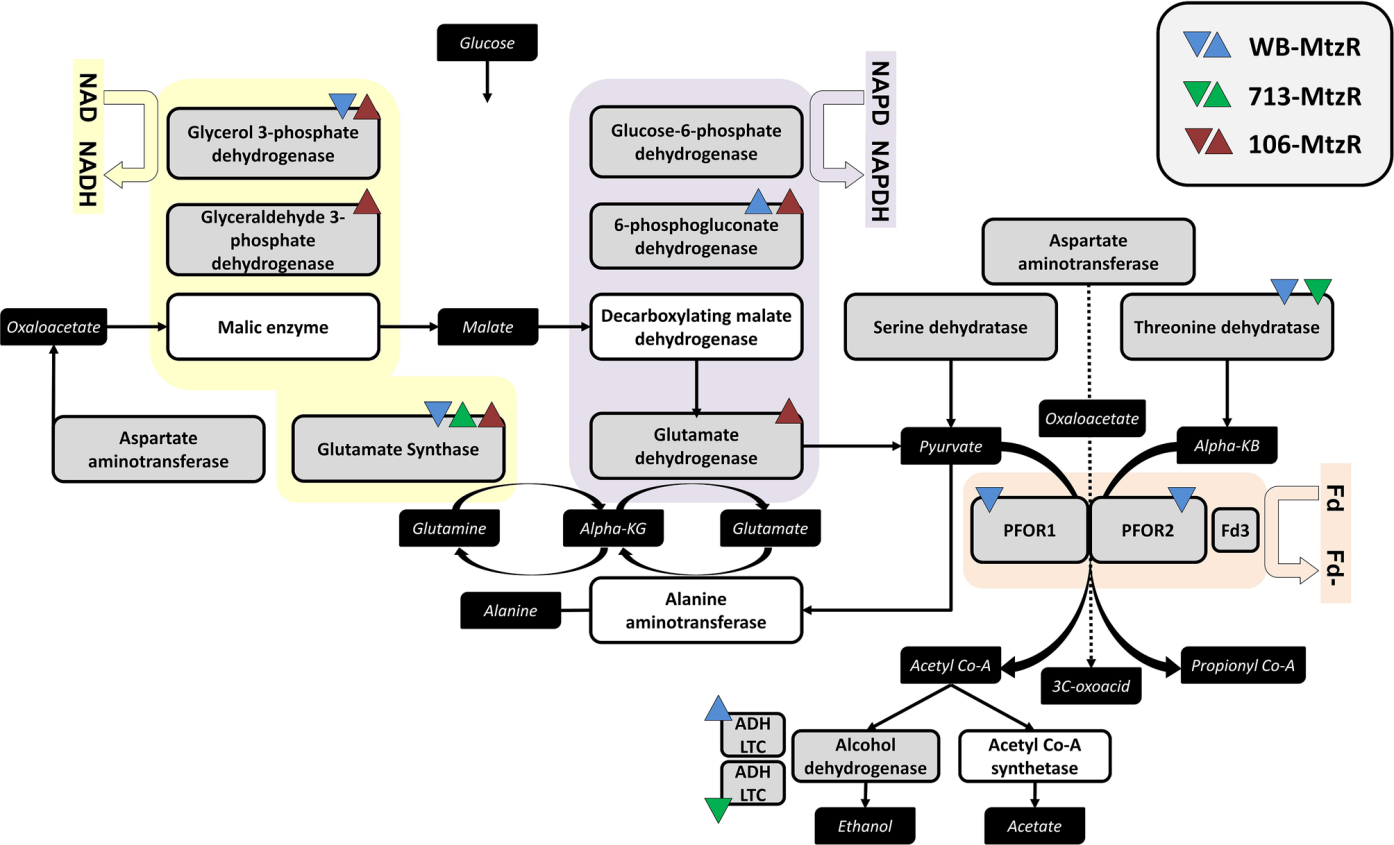
Differential protein expression in glycolysis and pyruvate catabolism. Pyruvate catabolism, with emphasis on enzymes with electron transport in upstream glycolysis. Enzymes with a white background were not identified in the protein dataset but have been included for completeness of pathway. Metabolites are shown in black boxes. Direction of differential expression in proteins is indicated using arrows and colors as designated in the top right corner. Fd, ferredoxin; ADH LTC, alcohol dehydrogenase lateral transfer candidate; KB, ketobutyrate; KG, ketoglutarate.

The extended oxidoreductase network was examined, specifically enzymes involved in cofactor abundance and electron transport, which showed trends towards upregulation in 106-MtzR (Fig. 3, panel 2). The magnitude of differential expression in these enzymes was lower overall than DEPs in the antioxidant network, and again no common expression profiles emerged between MtzR lines. While glutamate synthase was differentially expressed in all 3 lines, it was upregulated in 106- and 713-Mtz, and downregulated in WB-MtzR. Recent models suggest this enzyme may be more structurally similar to trimethylamine dehydrogenase of bacteria [44], which might indicate a role beyond amino acid metabolism (Fig. 4). Many of the changes related to enzymes consuming Nicotinamide adenine dinucleotide phosphate (NADPH) or NADH are better contextualized relative to electron transport via ferredoxin-containing enzymes (Fig. 4). Thus, 106-MtzR upregulates multiple enzymes that may increase electron transport and the reducing potential available to antioxidant enzymes that activate Mtz. Lower transcript levels of glutamate dehydrogenase in all 3 lines [18], hypothesized to similarly conserve NADPH, were not observed in protein expression, with 106-MtzR, in fact, displaying a higher abundance than its MtzS parents. In agreement with existing transcriptomics, threonine dehydratase was downregulated in WB- and 713-MtzR, perhaps indicating a preference for pyruvate over alpha-ketobutyrate as a PFOR substrate, as well as its downstream metabolite acetyl-CoA.

### Signaling and kinases

Proteins involved in signaling, predominantly kinases, were particularly enriched in WB-MtzR (30 DEPs), which were also detected in protein–protein interaction networks (Supplementary Fig. S4). A total of 12 such DEPs were detected in 106-MtzR, of which 5 were shared with WB (6700, 113 456, 17 069, 17 622, 3957), and 1 was shared between 106 and 713-MtzR (3677). The majority of differentially expressed kinases belonged to the uniquely expanded NEK kinase family in *Giardia*, with 16, 10, and 2 NEK kinases in WB, 106, and 713-MtzR lines, respectively. Multiple members of the NEK kinase family in *Giardia* are missing key catalytic amino acid residues and are predicted not to have catalytic activity [46]. Seven of the 16 DEP NEKs in WB-MtzR are predicted to lack activity, as are 5/10 in 106-MtzR and 1/2 in 713-MtzR. Interestingly, 4 of the 5 most upregulated NEK kinases in WB-MtzR were considered catalytically inert, as well as the most upregulated NEK in 106-MtzR.

An additional 14, 5, and 1 non-NEK kinases as well as 3, 5, and 1 phosphatases were differentially expressed in WB-MtzR, 106-MtzR, and 713-MtzR, respectively. In 106-MtzR 3 regulatory subunits of protein phosphatase type 2A (PP2A) activity were upregulated (9058, 4079, 17 538), while inositol 5-phosphatase 4 (9077) and another serine/threonine phosphatase (2053) were downregulated. In 713-MtzR the single serine/threonine phosphatase (2053) observed as downregulated was upregulated in the WB-MtzR line. Similarly, an additional 2 phosphatases in WB-MtzR were upregulated, including the PP2Ac phosphatase (5010), known to regulate encystation [47], and a PP2C phosphatase. Of the 14 differentially expressed, non-NEK kinases in WB-MtzR, 12 were upregulated, including a putative ethanolamine/choline kinase, while the 5 additional non-NEK kinases detected in 106-MtzR were downregulated, including a phosphatidylinositol-4-phosphate 5-kinase (gPI4P5K) (13 606) involved in lipid-based signaling [48].

### Lipid metabolism and membrane proteins

Multiple proteins involved in lipid metabolism in *Giardia* [48] were differentially expressed in at least 1 MtzR isolate. Among proteins involved in phospholipid metabolism, PI transfer protein alpha isoform (PITPα) (4197), a phospholipid-transporting ATPase IIB (gPLTATPase IIB), and a putative gPLTATPase IIB (38 104) were downregulated in 106-MtzR, while PS synthase (gPSS) (17 427) and another putative phospholipid ATPase transporter (16 592) were upregulated in 713-MtzR, with the later also upregulated in WB-MtzR along with another putative acyltransferase (15 987). PITPα was also downregulated in 106-MtzR. Furthermore, the highest upregulated kinase in WB-MtzR was the putative ethanolamine/choline kinase, implicated in phospholipid metabolism [48].

A range of proteins involved in fatty acid lipid metabolism were also among DEPs. These included 3 fatty acid acyltransferases in WB-MtzR, including significant downregulation of 1-acyl-sn-glycerol-3-phosphate acyl transferase (12 109) and upregulation of the glycylpeptide N-tetradecanoyltransferase homologue (5772) involved in protein myristoylation modifications. The majority of the long chain fatty acid CoA ligases (gLCFACL) were unchanged in expression in MtzR lines, though 17 170 was upregulated in both WB-MtzR and 713-MtzR. Among proteins involved in neutral lipid metabolism, only 1 putative phospholipase-B like protein (115 159) was identified, which was upregulated in 106-MtzR. Multiple proteins related to inositol signaling lipids were detected among DEPs, although none observed in 713-MtzR. In WB-MtzR upregulation of inositol 5-phosphatase 4 (gI5Pase) (9077) and inositol-3-phosphate synthase (17 579) proteins was observed, while 106-MtzR, in contrast, downregulated gI5Pase along with a putative gPI4P5K.

Additionally, a range of transmembrane and plasma membrane associated proteins were differential expressed in all MtzR lines, which have been summarized and sorted into functional categories in Table 3. Of the groups not previously addressed in the results section ‘EGF-Like Proteins and VSPs’, section 3.4, several peptidases were observed as differentially expressed throughout MtzR lines. Some of these were EFG-like, cysteine-rich endopeptidases, with 1 (14 225) universally downregulated in all 3 lines. Additionally, several different dipeptidyl-peptidases were differentially expressed, which have been previously implicated in regulation and signal transduction relating to encystation [49]. These dipeptidyl-peptidases, while potentially linked to amino acid metabolism, are also secreted proteins [50, 51], which may allow them to compete with host amino acid metabolism. Several members from the annexin-like alpha-giardins that interact with phospholipids [52] were exclusively observed only within downregulated proteins across MtzR lines, although no specific alpha-giardins were common between lines. Beta-giardin (4812), which is a microtubule associated protein in the adhesive disc [53], was also downregulated in MtzR lines.

**Table 3:** Differentially expressed membrane protein families in MtzR lines, including subgroups and protein and annotation features along with overall differential expression (DE) trends in MtzR lines

		Membrane association				
Group	Subgroup	TMH^a^	Extra/Intracellular domains	Additional features	DE trends	Key DE ORFs
EGF-like proteins	VSP	Yes	CXXC-rich extracellular	S-Palmitoyl necessary for lipid raft localization and lipid signaling [74]	Large DE in terms of proportion and magnitude, little specific variant overlap and variable directionality	GL50803_137 620
			CRGKA cytoplasmic tail	Citrullination of arginine [102]		GL50803_37 093
	HCMP	Yes	CXC/CXXC extracellular	Possible organelle membrane localization.	Several highly upregulated/downregulated variants, little specific variant overlap	GL50803_112 673
						GL50803_112 633
	Tenascin/Notch-like	Yes	EGF-like conserved site (IPR013032); usually extracellular	Some possess IPR013111 (EGF-like domain, extracellular)	DE with varying directionality between lines	GL50803_11 420
						GL50803_16 322
Peptidases	Cysteine-rich	Yes*	Growth factor receptor cysteine-rich domain (IPR009030)	Separate from VSP/HCMP, do not contain IPR005127 or IPR006212 annotations	Downregulation	GL50803_14 225
						GL50803_101 832
	Dipeptidyl-peptidases	Yes*	Serine-type (GO: 0 008236), cysteine-type (GO:0 008234) or dipeptidyl-peptidase activity (GO:0 008239)	Can localize to plasma membrane in absence of TMH [49]	Dipeptidyl-peptidase III upregulation [106]	GL50803_15 574
				Possible role in signal transduction, particularly during encystation [49]	Alanyl dipeptidyl peptidase downregulation (713, WB)	
					Dipeptidyl-peptidase I downregulation (WB).	
ABC transporters	Plasma membrane	Yes	IPR017871 (ABC transporter, conserved site)	Features some lipid-transporting ATPases	DE with varying directionality between lines	GL50803_115 052
						GL50803_16 592
Giardins	Alpha-Giardins	No	Calcium-depending phospholipid binding (GO:0 005544)	Localize to membrane and cytoskeletal structures, including as flagellar [52, 103–105]	Downregulation	
				Evidence for dual acylation [106]		
	Beta-Giardin	No	Cytoskeletal	Ventral disc localization [53]	Downregulation	GL50803_4812

ORFs listed in the last column were observed to be differentially expressed in at least 2/3 MtzR lines. *Some, but not all, members possess annotated TMH.

### Post-translational modifications in MtzR lines

**Figure 5:**
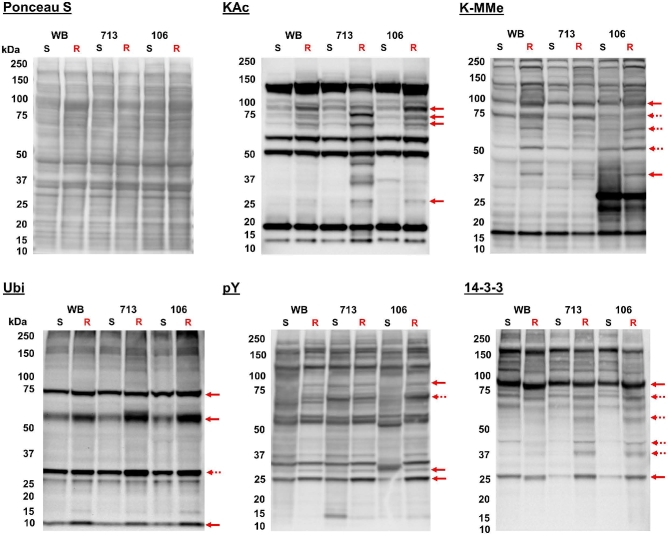
Western blots of post-translational protein modifications in MtzS and MtzR lines. Total protein lysate from trophozoites (15 μg) from WB, 713, and 106 MtzS and MtzR lines was probed with antibodies against acetylated lysine (KAc), mono-methylated lysine (K-MMe), ubiquitin (Ubi), phosphorylated tyrosine (pY), and the 14–3-3 binding motif (including phosphorylated serine) (14–3-3). Protein loading was verified after transfer using Ponceau S staining (first row, left). MtzS and MtzR lanes are designated by “S” and “R,” respectively. Altered protein features detected in 3/3 MtzR lines are designated with a solid red arrow, while protein features changed in 2/3 lines are designated with a broken red arrow.

Western blotting was used to assess protein PTMs in trophozoite lysates of each MtzS and MtzR isogenic line (Fig. 5). Overall, these PTM blots (phosphorylation, acetylation, methylation, and ubiquitination) changed in both the appearance of new modified protein features in MtzR lines, as well as increases in intensity (abundance) of modified protein features in MtzR lines compared to MtzS parents (Fig. 5). Total acetylated lysine (KAc) increased in all 3 MtzR lines compared to the MtzS parents. This included the appearance of multiple new KAc-modified protein features in MtzR lines, particularly in the 713-MtzR line. Four common protein bands (3: approximately 70–100 KDa; 1: approximately25–50 KDa) with detectable KAc increased in intensity in all MtzR lines; albeit the smallest of the 4 bands was more highly expressed in 106- and 713-MtzR. Another 3 bands (approximately 25–50KDa) with detected KAc increased in 713-MtzR. Mono-methylated lysine (K-MMe) also increased overall in all 3 MtzR lines, but most changes were unique to 1 or observed in 2 of the 3 lines. Presumptive histone proteins H3 (approximately 17kDa) and H4 (approximately 11kDa) were detected by anti-KAc antibodies at the same molecular weight and pattern as previously detected by anti-KAc in enriched fractions [54], while H3 protein was also detected by anti-K-MMe antibodies. Both KAc and K-MMe modification detection for histone variants is consistent with previously detected histone modification states via Western blot in *Giardia* [55], and subsequent immunoblotting with Anti-H3 and Anti-H4 showed that the modified protein bands at approximately 17 and approximately 11KDa in KAc and K-MMe blots aligned with antibodies for these histone variants (Supplementary Fig. S5).

In order to investigate the role of phosphorylation in MtzR, blots were performed to assess changes between isogenic lines in total tyrosine phosphorylation (pY) or the 14–3-3 substrate network. Although there were limited common fluctuations in pY modifications between lines, there were changes in the phosphorylated 14–3-3 substrate network. Changes in 14–3-3 sites included an increase and mass shift between MtzS and MtzR in a protein band at approximately 90kDa, as well an increase in a protein band at approximately 25kDA. These were accompanied by increases in 5 protein bands common between 106- and 713-MtzR lines. Of the 314 known 14–3-3 interacting substrates [56], 139 proteins were detected in all 3 isolates, and 52 proteins were detected in 1 or 2 of the isolates, constituting 60.8% of known substrates. However, only 57 of these substrates (18.2%) were detected among DEPs, indicating the majority of potential 14–3-3 substrates did not have significant changes in abundance. Western blots targeting ubiquitin also detected that 2 large protein bands at approximately 75 and approximately 55kDa increased in all 3 MtzR lines, as well increases in free ubiquitin (approximately 10kDa) in resistant lines.

There were also several notable differences occurring in the 106-MtzS line distinguishing it from the other 2 isolates. These include a unique KAc protein band in 106-MtzS at approximately 37kDa, as well as 2 unique, high-intensity protein bands with K-MMe at approximately 30 and approximately 25 kDa. Last, 106-MtzS displayed a divergent pY profile compared to WB- and 713 MtzS, although the 106-MtzR line displayed a more congruent pY profile compared to WB- and 713-MtzR.

### Chemical inhibitors of post-translational protein modification networks

**Figure 6:**
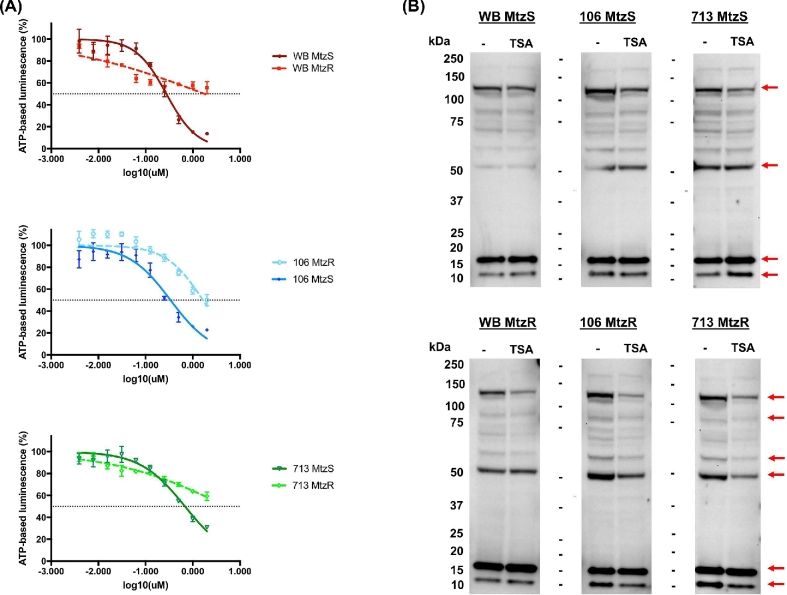
Results of trichostatin A exposure in MtzS and MtzR lines. (A) Dose–response curves for MtzR and MtzS lines of WB, 106, and 713 to deacetylase inhibitor TSA. Error bars represent ±1 standard deviation; experiments were performed in triplicate. (B) Western blots for total protein lysate from trophozoites (10 μg) exposed to TSA from WB, 713, and 106 MtzS and MtzR lines probed with antibodies against acetylated lysine (KAc). Trophozoites were exposed for 18 hours to 2 μM TSA, with control flasks exposed to the same volume DMSO as used as a vehicle for TSA exposure. Red arrows designate protein features with significant changes between TSA exposed and control trophozoites in the 6 lines.

Four broad-spectrum chemical inhibitors of PTM networks were compared in MtzS and MtzR lines: deacetylase inhibitor trichostatin A (TSA), broad-spectrum methyltransferase inhibitor chaetocin, kinase inhibitor staurosporine, and phosphatase inhibitor calyculin A. In addition to chaetocin, all other inhibitors have been previously verified for activity in *Giardia* [55, 57]. The dose–response curves, IC_50_, and resistance factors in MtzR lines for the 4 inhibitors are shown in Supplementary Fig. S6. Staurosporine, calyculin A, and TSA were all highly effective against *Giardia*, with wild-type parent IC_50_ >1μM. Chaetocin, however, was only slightly effective against *Giardia*. Of the 4 inhibitors, all MtzR lines had significant increases in IC_50_ to TSA compared to their MtzS parents (Fig. 6, panel A), indicating Mtz resistance correlated with cross-resistance to TSA [55, 57] (Fig. 6, panel A), with TSA resistance factors ranging from +4.8 to +7.5 in MtzR lines compared to MtzS parents.

To explore the effect of TSA on protein acetylation in trophozoites, TSA exposures were performed and protein lysate immunoblotted. Trophozoites exposed to TSA remained both viable and adhered until 18 hours. Trophozoites were detached but viable at 24 hours but were completely nonviable by 36 hours (data not shown). Exposure of trophozoites to TSA at 1μM, 2μM, and 4μM for 8 hours demonstrated hyperacetylation of histone variants (17 and 11 kDa) in parent, wild-type lines, but not MtzR lines (Supplementary Fig. S7). When trophozoites were exposed to 2μM TSA for 18 hours (Fig. 6, panel B), wild-type MtzS parent lines showed trends toward increased acetylation, as expected with the inhibition of deacetylases. However, MtzR isolates showed decreases in overall KAc, which was also observed across multiple concentrations of TSA at 8 hours (Supplementary Fig. S7). In contrast MtzR lines did not show significant increases in histone variant hyperacetylation after 8 or 16 hours of TSA exposure (Fig. 6, Panel B; Supplementary Fig. S7).

### MtzR stability after discontinuation of drug selection

Drug exposure was discontinued in 713- and 106-MtzR lines to observe effects on Mtz resistance (this was not undertaken in the WB-MtzR line due to impracticalities owing to its slow growth rate and lower adherence and confluence (Supplementary Fig. S8). MtzR lines were recovered and cultured for 1 week with Mtz drug selection, followed by 12 weeks of routine twice weekly passage (24 passages in total) without drug selection (Supplementary Fig. S9). Both MtzR isolates had lower confluence levels during original drug selection (week 1, P0; approximately 45%), which improved upon discontinuation of drug selection. In order to achieve higher confluence for routine passage twice a week, higher seed volumes were required between passages for both isolates compared to MtzS lines, an effect that diminished over time as gains in growth were observed (Supplementary Fig. S9). These improvements for *in vitro* growth occurred within 2 weeks in 106-MtzR, while fluctuations in growth rate requiring higher seeding volumes at passage were observed for 713-MtzR for up to 4 weeks (P8). As both WB-MtzR and 713-MtzR have reduced abundance of beta-giardin and SALP-1 (4410) proteins of the ventral disc [58], it is possible both lines have lower attachment (Supplementary Fig. S8) due to changes in the structure of the ventral disc and, therefore, lower confluence and slower division.

**Figure 7:**
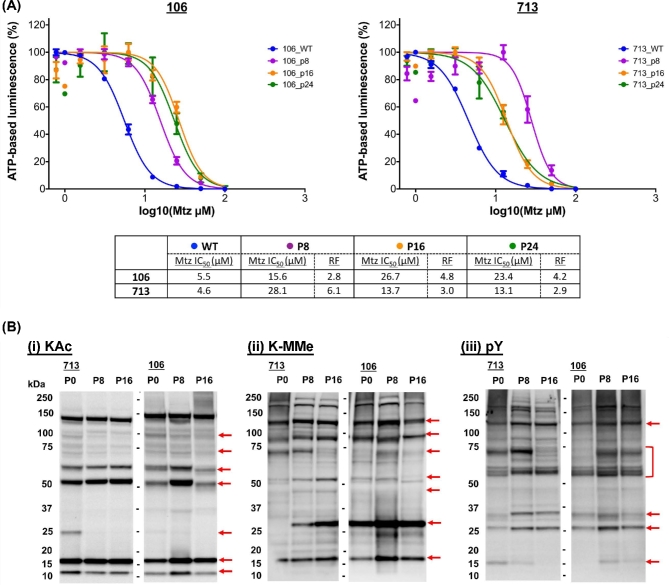
IC_50_ profiles and post-translational modifications upon discontinuation of Mtz selection. (A) Dose–response curves for MtzR lines of 106 and 713 upon discontinuation of drug selection at 4 (P8), 8 (P16), and 12 (P24) weeks compared to MtzS lines. MtzS lines were used and are designated 106_WT and 713_WT. Error bars represent ±1 standard deviation; experiments were performed in triplicate. The table records the calculated IC_50_ for each time point, with resistance factors calculated against the IC_50_ of the MtzS parent isolate. (B) Western blots against lysate from trophozoites (15 μg) from 713 and 106-MtzR (P0) and 4 (P8), 8 (P16) after discontinued drug selection was probed with antibodies against acetylated lysine (KAc), mono-methylated lysine (K-Mme), and phosphorylated tyrosine (pY). Altered features within 713 and 106 lines are designated on the right of the blot using a solid red arrow. Exposure times have been reduced by 20% to prevent overexposure of major bands (e.g., histone variants).

Mtz IC_50_ was calculated at each consecutive 4-week passage (P8, P16, P24; Fig. 7, panel A). After 4 weeks without Mtz selection, MtzR-P8 trophozoites in both lines had significantly increased Mtz susceptibility compared to MtzR lines (Table 1), with a lower IC_50_ in 106-MtzR-P8 of the 2 isolates. In MtzR-P16 trophozoites, 713-MtzR-P16 had further increases in Mtz susceptibility, while 106-MtzR-P16 increased in Mtz resistance compared to 106-MtzR-P8 to levels equivalent to its drug-selected parent (i.e., 106-MtzR at week 0; Table 1). After 12 weeks (P24) without drug selection, IC_50_ in both lines plateaued, with no significant change in Mtz sensitivity compared to week 8 (P16) trophozoites. Based on this, 106-MtzR-P16 and 106-MtzR-P24 trophozoites retained both drug resistance and improved growth rates (Supplementary Fig. S4) compared to their drug-selected parents [18]. At their lowest IC_50_ throughout the 12 weeks, neither MtzR line returned to levels of Mtz susceptibility as recorded in parent MtzS lines, with the lowest fold changes (resistance factor) in IC_50_ throughout the 12 weeks at +2.8 and +2.9 in 106-MtzR-P8 and 713-MtzR-P24, respectively.

### Post-translational network stability upon discontinuation of drug selection

Divergent IC_50_ profiles showed 713-MtzR had consecutive gains in drug susceptibility at week 4 (P8) and week 8 (P16) upon cessation of drug selection, while the 106-MtzR line displayed its lowest Mtz susceptibility at week 4, then Mtz resistance equivalent to parental MtzR lines at week 8. Given that large changes in PTM proteins were detected between MtzS and MtzR isogenic lines (Fig. 5), total protein lysate from MtzR lines at weeks 4 and 8 was probed for fluctuations in PTM networks upon cessation of drug selection (Fig. 7, panel B). KAc, K-MMe, and pY networks showed significant fluctuations between MtzR-P0 and MtzR-P8 and MtzR-P16, as well isolate-specific variations between 713- and 106-MtzR. There were changes in the intensity of modifications of H3 and H4 variants for both lines. In 106-MtzR, both histone variants at 4 weeks without Mtz had increased KAc and K-MMe, while by 8 weeks KAc and K-MMe modification levels still remained higher than MtzR selected lines on the H3 variant. In 713-MtzR, H4 KAc levels decreased, while H3 modifications were stable at 4 and 8 weeks. Together, these suggest that the cessation of Mtz exposure caused significant changes to epigenetically linked modifications.

Beyond histone proteins, KAc and K-MMe modification networks showed further fluctuations. The approximately 2-kDa band observed in MtzR isolates was lost upon discontinuation of drug exposure, as were several lower intensity bands between approximately 70–100 kDa. Two prominent bands observed at approximately 60 and approximately 50kDa showed significantly different intensity profiles between 713-MtzR-P0 and 106-MtzR-P0, increasing linearly in 713-MtzR-P8 and again in 713-MtzR-P16, while increasing in 106-MtzR-P8, then decreasing in 106-MtzR-P16 to levels lower than in 106-MtzR-P0 lines. Changes in the K-MMe modification networks increasingly diverged between 713-MtzR and 106-MtzR. Overall, 713-MtzR displayed a trend to increased K-MMe in 713-MtzR-P8 and 713-MtzR-P16, including appearance of the methylated protein band at approximately 25 kDa previously observed exclusively in 106-MtzS and 106-MtzR isolates (Fig. 5). As observed for KAc, K-MMe was highest in trophozoites at 106-Mtz-P8 and then decreased comparatively at 106-MtzR-P16.

Numerous protein features were detected with anti-pY antibodies, indicating many tyrosine kinase substrates in trophozoites (Fig. 5), which underwent widespread changes upon discontinuation of Mtz exposure. Although a unique pY modification profile was observed for 106-MtzS (Fig. 5), neither 106-MtzR-P8 nor 106-MtzR-P16 showed the same profile observed in 106-MtzS. However, by the end of 12 weeks, the 106-MtzR line still possessed significant levels of Mtz resistance compared to its MtzS parent. There were multiple protein bands with divergent profiles in MtzR lines between isolates in the absence of drug selection, including 2 bands at approximately 27 and approximately 30 kDa as well as multiple bands clustered between approximately 60–70 kDa, although 1 protein band at approximately 125 kDa increased in intensity in both isolates. Overall, discontinuation of Mtz selection widely altered phosphorylation signaling in both isolates.

## Discussion

Given the limited options for treating microaerophilic parasites, Mtz resistance is a major obstacle in the control of giardiasis and a widespread issue for metabolically related pathogens treated with nitroheterocyclics. To our knowledge, our study provides the first quantitative proteomic data for any MtzR pathogen. Further, we performed the first exploration of protein PTM networks in MtzR parasites and identified substantial changes in 4 major protein modification networks (Fig. 5). This indicates that PTM and differential protein expression may both contribute to Mtz resistance phenotypes. Further, we demonstrated trends of increased protein acetylation in MtzR lines, as well as cross-resistance to the histone deacetylase inhibitor TSA (Fig. 6, panel A), which also produced divergent KAc profiles in exposed MtzS and MtzR lines (Fig. 6, panel B; Supplementary Fig. S7). Perhaps most significantly, this study documented changes in Mtz susceptibility in 2 MtzR lines upon cessation of drug selection, and we observed IC_50_ variation between isolates and widespread fluctuations in 3 PTM networks (Fig. 7). However, a key aim of this 3-way, isogenic analysis of MtzR lines was to reconcile observed inconsistencies of passive and active MtzR traits and isolate-dependent variation [2]. Our results show that although there was some equivalency at the functional level, differentially expressed proteins diverged considerably between isolates (Fig. 1, panel A), within protein families (Fig. 2, panel B and C), networks (Fig. 3), and pathways (Fig. 4). When interrogating post-translational networks, again multiple isolate-specific features differentiated each MtzR line (Fig. 5), including some preexisting, unique features in parental MtzS lines. Last, isolate variation was evident in Mtz sensitivity and PTM profiles upon exposure to TSA (Fig. 6, panel B) and cessation of Mtz selection (Fig. 7). Overall, we believe this study confirms that MtzR is polygenic, plastic, and likely post-translationally regulated, with particularly strong links between Mtz resistance and protein acetylation. Our data therefore support earlier hypotheses that suggest links between MtzR and epigenetic regulation [18, 19], expand previously limited data concerning post-translation modifications in MtzR [20], and highlight for the first time that changes in post-translational modifications in MtzR are widespread.

### Oxidoreductases, electron transport, and active/passive MtzR mechanisms

The unique glycolytic and antioxidant system in aerophiles has been a primary focal point in the study of Mtz resistance, including for *Giardia*. Our results indicate that NR-1 (22 677) was downregulated in all MtzR lines, which concurs with previous results [16, 17, 19], including transcriptomics results for the same lines [18]. Previously, recombinant NR-1 was shown to reduce (i.e., activate) Mtz using NADH as a donor [59]. Ansell et al. [18] subsequently hypothesized that NR-1 could use electrons from ferrodoxin to reduce Mtz, which could functionally link NR-1 to pyruvate catabolism and, by extension, to the PFOR-ferredoxin electron transport chain (Fig. 4). Taken together, downregulation of NR-1 is the strongest candidate for a universal passive resistance mechanism. Downregulation of PFOR expression was observed only in WB-MtzR in this study, which agrees with transcriptomic data for WB-MtzR and other resistant clones in this genotype [18, 19, 39], although not all clones [14]. It is also possible that reduced PFOR enzyme activity, previously observed in 106 and WB Mtz resistant lines [10, 12, 28], might lower the rate of Mtz activation to be functionally analogous as decreased PFOR protein expression. As such, combined investigations of enzyme activity and protein expression may be required to test the interaction of different regulatory mechanisms in Mtz resistance.

Thioredoxin reductase was also inconsistent as a passive resistance mechanism, up- and downregulated in 106-MtzR and WB-MtzR, respectively, but unchanged in 713-MtzR. This may be due to expression-independent changes to enzyme activity [12] or, alternatively, that downregulation of thioredoxin reductase negatively impacts downstream thiol antioxidant systems, outweighing benefits of decreased Mtz activation. While downregulation of thioredoxin reductase in WB-MtzR was accompanied by decreases in thioredoxin, in 106-MtzR, the enzyme substrate of the increased reducing power postulated to result from upregulated thioredoxin reductase is unclear. The wider profile of the oxidoreductase network also indicates a multiplicity of isolate-dependent expression patterns (Fig. 3). For example, changes in the expression of oxidoreductases contributing to cofactor abundance and electron transport (Fig. 3), including upregulation of multiple enzymes involved in NAD(P)H consumption and production, were observed in 106-MtzR. Despite the absence of shared differentially expressed oxidoreductases, all MtzR lines exhibited functional enrichment of electron carrier activity (Fig. 2, panel B), indicating that Mtz variably affects the reducing power in the antioxidant network, the electron acceptors within glycolysis, as well as the critical relationship between them.

Some isotype-specific passive resistance mechanisms were also observed at the functional level. WB-MtzR downregulated 29 structural ribosomal proteins, which was similarly observed in transcript data [18] and suggests significant reduction in ribosomal levels, protein production, and *in vitro* generation times. While 106-MtzR downregulated only 5 structural ribosomal constituents, 2 of the 4 genomically encoded FtsJ domain-containing rRNA methyltransferases (6055, 16 993) were upregulated. Null mutations in FtsJ are linked to altered ribosomal structures and impaired growth rates [60, 61] and indicate potential links between post-translational methylation, growth rate, and ribosome function in MtzR lines.

### Cysteine-rich and membrane-associated proteins and lipids

Increasing evidence suggests that oxidative damage to cell membranes and their constituents can disrupt downstream, lipid-based signaling [62, 63]. A range of proteins associated with lipid metabolism or the membrane (Table 3) were differentially expressed in each MtzR line, along with kinase and non-kinase proteins linked to lipid-based signaling. Oxidative damage to the membrane, particularly via lipid peroxidation or mutually competitive modifications, is well documented in other systems [62], but not in *Giardia*, nor specifically in the context of oxidative stress or Mtz resistance. As such, metabolic analyses of the lipid and oxylipid composition of MtzR and MtzS lines would offer insights into the relationships between oxidative stress, Mtz, and resistance.

Phospholipid-transporting ATPases (gPLTATPase) and gPSS have been suggested to form crucial links in the import and metabolism of phospholipids [48] and were upregulated in WB-MtzR along with multiple genes involved in fatty acid intake, synthesis, modification, and metabolism. This suggests lipid metabolism may be increased in some MtzR isolates, possibly to ensure membrane integrity for signaling pathways. In addition, phosphatidylinositol pathways, which are associated with regulating cell growth in *Giardia* [48], were upregulated in WB-MtzR, which has impaired growth rates compared to its MtzS parent [18]. In WB-MtzR, the highest upregulated kinase was a putative choline/ethanolamine kinase responsible for initiating synthesis of phosphatidylcholine and phosphatidylethanolamine via phosphorylation. In *Plasmodium falciparum*, inhibition of choline/ethanolamine kinases leads to a significant decrease of phospholipids and arrest of parasite growth [64] and suggests links between lipid composition, oxidative damage, and growth in WB-MtzR. In contrast, 106-MtzR downregulated phospholipid import and metabolism genes, as well as phosphatidylinositol pathways. Additionally, membrane-associated dipeptidyl peptidases, which are known to regulate crucial proteolytic events during encystation [49], were differentially expressed across all lines, suggesting the importance of membrane signal-transduction events in *Giardia* MtzR phenotypes. Further, differentially expressed alpha-giardin proteins, which are known to interact with phospholipids in the membrane, were exclusively downregulated in MtzR lines. Membrane lipid composition, such as un/saturation of phospholipid fatty acyl components, is known to change the sensitivity of the membrane to oxidative stress and damage [65], and increased fatty acid unsaturation is dynamically regulated during encystation in *Giardia* [66]. Although not detected in the proteomic dataset, at the transcript level [18], fatty acid elongase 1 (gFAELO) (92 729) was upregulated in WB-MtzR and 713-MtzR, along with 3 gLCFACL long chain fatty acid CoA ligases, providing further evidence of fatty acid composition changes in the membranes of MtzR isolates.

VSP variants in MtzR lines displayed similar trends in the proportion and magnitude of differentially expressed VSPs, although specific VSP variants and the direction of differential expression diverged, and MtzR lines were substantially different from MtzS parents and each other (Fig. 2, panels B and C). This divergence may be a product of spontaneous VSP-variant switching [67] and differences in both time in culture and generation time for the 6 lines. However, large changes in VSP expression and turnover have been observed in other oxidative stress experiments [68], and it remains unclear if a specific variant may have functional contributions to limit oxidative damage. Both VSP and HCMP gene families are rich in cysteine, which is the major low-molecular-weight thiol in *Giardia* [69], and their multiple CXC/CXXC motifs potentially contribute to thiol and redox chemistries [70, 71]. Proteomic methods are available that allow selective modification to identify and discriminate reversible and irreversible oxidation states in cysteine [72, 73], which would provide evidence of the states of the multiple cysteines in VSPs, including those known to be lipid modified [74]. While further evidence is required to determine whether specific *Giardia* VSPs contribute to alleviating oxidative stress [68], including Mtz-induced oxidative stress [18, 39], collective VSP protein expression as a measure of antigenic switching rates may sensitively indicate significant restructuring of populations following xenobiotic or physiological stress. Large and statistically significant changes in VSP expression have been previously detected during proteomic analyses of encystation [75] and *in vitro* host–parasite models [41] in shorter timeframes than reported for spontaneous antigenic switching [76]. This indicated that VSPs may be one of the most dynamic and sensitive protein families within *Giardia*.

### Post-translation modifications networks in MtzR lines

To date, the role of protein modifications in MtzR has been inferred through differential expression of their modifying enzymes [15, 18, 19] rather than modified substrates. Our results indicate that multiple proteins within MtzR lines are differentially modified by ubiquitination, phosphorylation, methylation, and acetylation on multiple protein substrates. These modification marks varied in intensity and detectability within MtzR lines (Fig. 5). This is significant if increases in these modifications are in site occupancy unaccompanied by increased protein abundance, as it would indicate regulation independent of expression levels. Probes for ubiquitin revealed 3 common protein bands that increased in MtzR lines, including free ubiquitin, and a fourth band that increased in 713- and 106-MtzR lines only (Fig. 5). Seven ubiquitination and proteasome-related proteins were differentially expressed across MtzR lines, 6 of which were in WB-MtzR. This correlates in WB-MtzR with significant differential expression of both ribosomal proteins as well as ribosomal transcripts [18], indicating a potential for heightened demand of protein production to compensate for turnover of damaged proteins. Ubiquitination blots were highly congruent between isolates and lines, although only major protein bands were detected and lower abundance proteins may not have been detected. As mono-ubiquitinated proteins are associated with proteasome-independent processes, including gene transcription, while poly-ubiquitinated proteins are more likely to be associated with proteolysis and proteasome-degradation [77], analysis of the states of ubiquitination in MtzR lines would allow further extrapolation of the role of this modification in resistance.

*Giardia* has a uniquely reduced core kinome and a significantly expanded NEK kinome that consist of 80 and 278 kinases, respectively [46]. Our results indicated that both core and NEK kinases were differentially expressed and significantly enriched during functional clustering within MtzR lines. To survey changes to the phosphoproteome, we examined tyrosine phosphorylation and serine/threonine phosphorylation in the conserved 14–3-3 binding motif [(R/K)XX(s/t)XP] via Western blot. *Giardia* has a single 14–3-3 homologue with 314 documented protein substrates [56]. Immunodetection of the 14–3-3 motif revealed multiple novel and increased intensity protein features in MtzR lines relative to the isogenic parents (Fig. 5). However, our protein expression data showed that although 60.8% of known 14–3-3 substrates were detected among identified proteins, only 18.2% were among differentially expressed proteins, implying that the stoichiometric balance between modified and unmodified protein isomers is more variable than substrate abundance. Western blots of pY reinforce observations of Manning et al. [46] that despite the absence of canonical tyrosine kinases or tyrosine-kinase like (TKL) kinases in the *Giardia* genome, pY is readily detectable and abundant across the proteome (Fig. 5 and 7, panel B). MtzR lines were quite congruent between isolates for pY and (excluding the unique pY profile of 106-MtzS) shared many features with their MtzS parents. With pY most likely to be catalyzed by dual-specificity serine-threonine kinases [46], of which a wide range were differentially expressed in MtzR lines, including within the NEK kinase family, more work is required to define pY kinases in *Giardia*.

Dose–response curves for kinase inhibitor staurosporine and phosphatase inhibitor calyculin A (Supplementary Fig. S6) were similar and overlapping in MtzR and MtzS lines, with only minor shifts in IC_50_ between lines. Staurosporine and calyculin A have significant efficacy against *Giardia*, with IC_50_ for both compounds >50 nm (Supplementary Fig. S6), and have also been shown previously to block entry into and prevent exit from mitosis, respectively. Although their enzyme specificity and targets are not known [57], there is nonetheless a need for more targeted inhibitors to functionally probe specific phosphorylation pathways or enzymes between Mtz-resistant and -susceptible lines [57]. Therefore, more work is required to annotate core kinases in *Giardia* into subfamily classifications, define their homology, and, more broadly, screen chemical inhibitors of phosphorylation.

Acetylation (KAc) may be an important histone modification in MtzR; however our data reveal that KAc modifications occur and change on a wide range of protein substrates (Fig. 5 and 7, panel B). These changes appear linked to Mtz resistance, with all 3 MtzR lines showing increases in total KAc across a range of nonhistone proteins compared to their MtzS parents and cross-resistance to deacetylase inhibitor TSA (Fig. 6, panel A). This implies significant changes in lysine acetylase (KAT) or deacetylase (KDAC/HDAC) activity, which is further evidenced by differential KAc profiles and a lack of histone hyperacetylation in MtzR lines during TSA exposure (Fig. 6, panel B; Supplementary Fig. S7). There are 5 lysine acetyltransferases (KATs) and 6 lysine deacetylases (KDACs) encoded by the *Giardia* WB genome [54], which include 5 nuclear (4 sirtuin [Sir2] KDACs and 1 NAD^+^-independent HDAC) and 1 cytostolic (a Sir2 KDAC) deacetylase in trophozoites [55]. Although TSA is considered a class I and II, but not class III, (sirtuin) HDAC inhibitor, some *Giardia* sirtuins have very low homology to mammalian families [55] and more work is required to demonstrate that TSA specifically inhibits the single NAD^+^-independent HDAC in *Giardia*. Furthermore, given changes in KAc in response to TSA were not confined to histone variants (Fig. 6, panel B; Supplementary Fig. S7), HDAC substrates also require identification. In Ansell et al. [18], 4 of the 5 KATs were transcriptionally downregulated in 713-MtzR, along with numerous N-acetyltransferases. Among K/HDACs, the cytosolic Sir2 (10 708) is significantly upregulated at the transcript [18] and protein levels in WB-MtzR, and the NAD^+^-independent deacetylase (HDAC) is upregulated at the protein level in 106-MtzR [55]. Sir2 KDACs are linked to increased longevity, antioxidant gene expression, and cell cycle regulation in yeast during oxidative stress [78], while sirtuins in high eukaryotes are known to ameliorate oxidative stress by deacetylating enzymes and increasing their antioxidant activity [79, 80]; similar diverse KAc regulation may function in *Giardia* in MtzR lines.

KAc derives from acetyl-CoA, a substrate for protein acetylation, and is thus closely linked to central metabolism. In *Giardia*, acetyl-CoA production is downstream of PFOR and the ferredoxin-based electron transport metabolism [81]. The PFOR metabolic node is perturbed by multiple mechanisms in MtzR lines (Fig. 4), which may influence acetyl-CoA availability and, in turn, KAc modifications [18]. As such, MtzR phenotypes with either increased production or decreased metabolism of acetyl-CoA may have more substrate available for higher rates of KAc in MtzR lines [82, 83]. Metabolite-linked increases in KAc rates and substrates have been observed in *P. falciparum*, where addition of acetate increased the intracellular acetyl-CoA pool and downstream protein acetylation in rates of site occupancy, including for transcription factors and on histone variants [84]. As such, our observed increase of KAc in MtzR lines could trace back to both redox-regulated enzymatic (Sir2) as well as altered metabolic (acetyl-CoA) sources. Measuring acetyl-CoA levels in MtzS and MtzR lines would provide insight into metabolic disruptions downstream of PFOR and pyruvate metabolism. However, additional information on KAc substrates is still required, as these may also occur on, and influence activity of, key redox proteins in MtzR.

The *Giardia* genome encodes a highly reduced methylation network, with 6 histone lysine methyltransferases, but no canonical arginine methyltransferases or demethylases [54, 85], and 3 methylation states (mono-, di-, and tri-methylation) observed on *Giardia* histone variants [55]. This significant reduction of the methylation network may explain the high IC_50_ of the broad-spectrum histone lysine methyltransferase chaetocin (Supplementary Fig. S6), which is significantly more effective in mammalian systems [86]. Substrates of lysine methylation beyond histones are unknown, although our results have demonstrated K-MMe is an extensive modification network in *Giardia* trophozoites (Fig. 5 and 7, panel B). Protein methylation modifications do not neutralize the substrate amino acid charge, as in acetylation and phosphorylation, or produce a significant mass shift as in ubiquitination. However, methylation still influences accessibility of protein–protein interactions and binding, particularly in gene regulation, where multiple methylation states are observed on histones, transcription factors, and DNA-modifying enzymes [87]. All 3 MtzR lines showed increases overall in total K-MMe (Fig. 5), with many features unique to each isolate or line. Three and 2 of the *Giardia* histone methyltransferases, which were below detection in our proteomic dataset, are differentially transcribed in 713- and WB-MtzR relative to MtzS parental lines [18]. Conversely, in 106-MtzR, these enzymes are unchanged at the transcriptional level. However, at the protein level, these enzymes displays upregulation of a putative S-adenosylmethionine-dependent methyltransferase and 2 FtSJ rRNA methyltransferases. Although we can confirm lysine methylation as a correlate of MtzR across the proteome, substrate identities are now required.

### Fitness costs, isolate variation, and MtzR stability across lines

*In vitro* acquisition of MtzR variably affects trophozoite fitness among MtzR lines relative to their MtzS parents, implying varying clinical *in vivo* relevance. The *G. duodenalis* WB-MtzR line exhibits markedly decreased growth rate with the development of MtzR compared to its susceptible parent [18] and lower rates of trophozoite adherence and confluence (Supplementary Fig. S8). Reduced growth rate is also reported for other MtzR lines [19], some of which also failed to encyst *in vitro*. While *in vitro* growth appears less affected in 106-MtzR and 713-MtzR lines, the ability of trophozoites to attach *in vitro* to inert surfaces (culture tubes) and *in vivo* to gastric epithelium (suckling mice intestine) is significantly impaired [14]. Of the 3 lines explored here, only 106-MtzR retains infectivity in suckling mice [14].

Our results add to evidence that demonstrates that MtzR exacts significant metabolic costs to *G. duodenalis* and also highlights variability in the magnitude and reversibility of MtzR changes between and within genotypes. Indeed, the impaired growth rate and lower confluence of WB-MtzR relative to other MtzR lines [18] (Supplementary Fig. S8) meant it was not possible to compare passage and time from cessation of drug selection equivalently as for 713- and 106-MtzR lines. Further, 106-MtzR recovered from slower growth and lower confluency more quickly than 713-MtzR upon discontinuation of drug exposure (Supplementary Fig. S9). Removing Mtz selection corresponds to restored transcription and enzyme activity of drug-activating enzymes and increased drug susceptibility [14, 21] in as little as 4 months. Transcriptomic analyses of WB, 713, and 106 isogenic lines indicated qualitative differences between 106-MtzR and WB- and 713-MtzR lines [18]. Correlations with previous studies revealed the 106-MtzR transcriptome to be most similar to the “wild-type” WB-MtzS after exposure to sub-lethal Mtz [15]. The 106-MtzR molecular phenotype is also potentially the most clinically relevant, in that it grows relatively quickly and remains cytoadherent and infective in suckling mice, while also showing a relatively stable MtzR phenotype after discontinuation of Mtz selection [14].

The source of isolate variation in Mtz tolerance both in the presence and absence of drug, particularly in context of the 106-MtzR line, is not clear. There are unresolved chromosomal aberrations in all 3 MtzR isogenic lines [88–90] as well nonsense mutations in multiple transcripts, including in NR-1 in 106-MtzR [18]. Western blots of protein post-translational modifications showed preexisting differences in 106-MtzS for KAc, K-MMe, and pY networks (Fig. 5). Epigenetic regulation of transcriptional plasticity is implicated in MtzR and its stability, as the encystation–excystation process involves extensive epigenetic remodeling [54] and restores Mtz susceptibility in formerly MtzR lines [19]. The lack of a histone H1 linker in *Giardia* has been proposed to shift reliance to histone modifications for chromatin remodeling and gene regulation [91], with acetylation and methylation already demonstrated as regulators of chromatin state for key processes of antigen switching and encystation [55, 92]. Although widespread changes were detected for KAc and K-MMe modifications between MtzR and MtzS lines, many of these occurred on nonhistone substrates (Fig. 5). In contrast, cessation of Mtz selection produced significant changes, particularly on H3 and H4 modifications in MtzR lines (Fig. 7, panel B) albeit in an isolate-dependent manner. Although histone acetylation generally correlates with transcriptional activation, methylation occurs in mono-, di-, and tri-methyl moieties that differentially influence gene expression and DNA methylation [87, 93]. Together, this indicates that a suite of epigenetically regulated fluctuations are likely to be occurring in transcription in 106-MtzR at 4 and 8 weeks and that MtzR may be regulated through different mechanisms in 713-MtzR. Given our emerging understanding of the links between histone modifications in oxidative stress responses [94], differences in Mtz- and oxygen-induced oxidative stress loads postulated in these lines may differentially influence epigenetic transcriptional phenotypes via epigenetic induction.

### Conclusion

This study used 3 well-characterized isogenic MtzS and MtzR lines to investigate correlates of resistance at the proteomic and post-translational levels in a genetically controlled design. Substantial genotypic variation was found in DEPs and post-translational marks. Together, these data regarding post-translational modifications as well as quantitative proteomics of protein abundance represent the most comprehensive post-transcriptional analysis of any pathogen in the context of nitroheterocyclic resistance to date and suggest Mtz resistance, at least in *Giardia*, is significantly more complex than previously thought. Our data confirm that Mtz induces significant changes within proteins in antioxidant, electron transport, and pyruvate catabolism networks in *Giardia*, of which NR-1 downregulation coincides with multiple observations at the transcript level as a universal feature among a multiplicity of isolate-specific expression profiles (Fig. 3). Our data also provide the first evidence to credit hypotheses that link acetylation to Mtz resistance, with increased acetylation in MtzR lines (Fig. 5), as well as substantial cross-resistance to deacetylase inhibitor TSA (Fig. 6). Further, given the relationships between KAc, PFOR expression, and production of acetyl-CoA, we hypothesize that links exist between Mtz resistance, metabolism, and protein modifications.

Last, we also provide novel insights through longitudinal surveillance of MtzR after discontinuation of drug selection, highlighting the loss of plastic traits and the potential of stable resistance traits. Our results also add to data that suggest 106-MtzR is both a clinically relevant [14] and transcriptionally unique [18] isotype, avoiding major fitness costs and retaining its MtzR phenotype *in vivo* and *in vitro* at parental MtzR IC_50_ levels at 12 weeks (Fig. 7, panel A). Among a range of changes observed at the level of post-translational modifications, 106-MtzR showed significant fluctuations in KAc and K-MMe modifications of H3 and H4 variants (Fig. 7, panel B), the first evidence to implicate epigenetic modifications in the stability of Mtz resistance. The interrogation of specific acetylation and methylation marks, as performed for differentiation and antigenic switching [55], would be the next step in investigating the role of chromatin state and transcriptional plasticity in Mtz resistance. Furthermore, given cross-resistance of Mtz resistance lines to TSA, further screening and characterization of specific and targeted epigenetic, as well as broader PTM, chemical inhibitors are required to further probe post-translational regulation of Mtz resistance.

## Methods

### Isogenic isolate cell culture

Trophozoites from each isolate were maintained in flat-sided 10-mL tubes (Nunclon delta) filled with complete TYI-S33 medium [81] containing 6 mM glucose and subcultured twice weekly. Mtz-resistant lines were cultured in the presence of Mtz (Sigma Aldrich; 100 mM stock dissolved in dimethyl sulfoxide [DMSO]) at a final concentration of 30 μM, while parental lines were maintained in 1% DMSO. The Mtz-sensitive lines used in this study included WB1B (WB-MtzS), BRIS/83/HEPU/106 (106-MtzS) and BRIS/87/HEPU/713 (713-MtzS) along with their respective Mtz-resistant progeny lines WB1B-M3 (WB-MtzR), BRIS/83/HEPU/106-2ID10 (106-MtzR) and BRIS/83/HEPU/713-M3 (713-MtzR). IC_50_ for Mtz for resistant and susceptible isolates was previously determined as detailed in Ansell et al. [18]. Isolate nomenclature and references for axenization and induction of Mtz resistance induction are provided in Table 1.

### Protein extraction, digestion, and TMT labeling for proteomics

Trophozoite cultures for protein extraction were generated as previously described [18]. Briefly, trophozoites were seeded at a number normalized to growth rate in order to achieve equivalent final cell numbers in t25 flasks (Falcon), followed by decanting of media and unattached and nonviable trophozoites. Adherent trophozoites were then harvested by chilling trophozoites in fresh, complete TYI-S33 media on wet ice before collection by centrifugation. Total protein, RNA [18], and DNA were extracted using the TriPure reagent (Roche) from the same trophozoite pellet material according to the manufacturer's instructions.

Protein pellets were solubilized in 2% sodium dodecyl sulfate (SDS) in 50 mM Tris (pH 8.8) (Sigma Aldrich) before reduction in 5 mM dithiothreitol followed by alkylation in 10 mM iodoacetamide in the dark (with alkylation quenched with 5 mM dithiothreitol). To remove interfering reagents, proteins were precipitated via the methanol/chloroform approach [95] and transferred to 8 M Urea in 50 mM Tris (pH 8.8) and protein concentration quantitated by bicinchoninic acid (BCA) assay (Pierce). A 2-stage digestion was performed first with Lys-C (Wako) overnight at 30°C (1 μg enzyme to 100 μg protein), followed with Trypsin (Promega) digestion at 37°C (1 μg enzyme to 100 μg protein) for 6 hours. Samples were acidified with trifluoroacetic acid to 1% concentration acid and then desalted using solid phase extraction (SPE) with in-house tips packed with styrene divinyl benzene (3M Empore). Peptide extracts were dried by vacuum centrifuge, reconstituted in 200 mM N-(2-Hydroxyethyl)piperazine-N^΄^-(2-ethanesulfonic acid) (pH 8), followed by quantification via Micro BCA (Pierce).

For TMT labeling, 35 mg of peptides per sample were used for each TMT label reaction. Samples were labeled across 3 TMT 10-plex reactions (Thermo, San Jose, CA) using 0.14 mg of each reagent, with each of the 3 TMT 10-plex experiments containing MtzS and MtzR replicates of each of the 3 isolates (WB, 106, and 713). Samples were incubated with labels for 1 hour at room temperature and then quenched with 5% hydroxylamine (Sigma Aldrich). Each of the 10 labeled samples for each of the 3 TMT 10-plex experiments were combined, dried by vacuum centrifuge, reconstituted in 1% formic acid, and desalted on a 200-mg C18 SepPak (Waters, Massachusetts) prior to strong cation-exchange (SCX) fractionation as described previously [41]. A total of 10 pooled SCX fractions were desalted using SPE as before, dried down using a vacuum centrifuge, and reconstituted in 1% formic acid for nanoflow liquid chromatography tandem mass spectrometry (nanoLC-MS/MS).

### Nano LC-MS/MS of TMT-labeled peptides

MS analysis was performed on a Q Exactive Orbitrap (Thermo Scientific) coupled to an EASY-nLC1000 (Thermo Scientific) as previously described [41]. Reversed-phase chromatographic separation was performed on a 75 μm id. × 100 mm, C18 HALO column, 2.7 μm bead size, 160 Å pore size. Samples were run on a linear gradient of 1–30% solvent B (99.9% acetonitrile/0.1% formic acid) over 170 minutes, with the Q Exactive operating in the data-dependent mode to automatically switch between Orbitrap MS and ion trap MS/MS acquisition. Survey full-scan MS spectra (from m/z 350 to 1850) were acquired with a resolution of 70,000 at m/z 400 and an automatic gain control target value of 1 × 10^6^ ions. The top 10 most abundant ions were selected for higher-energy collisional dissociation (HCD) fragmentation, with the HCD normalized collision energy set to 35% and fragmentation ions detected in the Orbitrap at a resolution of 70,000. Dynamic exclusion of target ions selected for MS/MS was set to 90 seconds, and the lock mass option was enabled using the polydimethylcyclosiloxane ion (m/z 445.12003) as an internal calibrant.

### Database searching

Raw data files produced in Xcalibur (Thermo Scientific) were processed in Proteome Discoverer V1.3 (Thermo Scientific) and searched using Mascot against the WB C6 (ATCC 50 803), V5.0, genome release obtained from GiardiaDB.org [50]. Parameters and modifications were as follows: MS tolerance was set to ±10 ppm, MS/MS tolerance was set to 0.1 Da, 1 missed cleavage was allowed; static modifications were set for carbamidomethylation of cysteines, while variable modifications were set to TMT 10-plex modification of peptide N-termini and lysine residues, methionine oxidation, and deamidation of asparagine and glutamine. Search results only included peptides with a score >15 and below the Mascot significance threshold filter of *P* = 0.05. False discovery rate (FDR) was set for 1%. Protein grouping for homologous peptide identification was enabled such that protein identifications were based of peptides with amino acid sequences equal to or contained within the sequence of more than 1 protein; the 2 proteins were grouped together in a single protein group. The MS raw data files, database search results, and TMT ratios have been deposited to the ProteomeXchange Consortium [29] via the PRIDE partner repository with the dataset identifier PXD007183.

### Analysis of differentially expressed proteins

Relative quantitation of protein abundance in MtzR compared to MtzS isogenic lines was derived from the ratio of the TMT label detected in each MtzR to MtzS replicate. As such, there were 9 ratios for each MtzR vs MtzS comparison, and the geometric mean was calculated to establish the fold change for each protein identified. Further to ratio-derived fold changes, protein abundance between MtzR and MtzS lines was evaluated statistically via a 1-sample *t* test using the tenth channel (pooled control) to normalize MtzR and MtzS replicate labels. Differential expression required proteins to meet both ratio fold change (<1.3 or >0.77) and a significant *P* value (> 0.05) [36, 37]. Further statistical evaluation of the dataset was performed, with an unsupervised multivariate PCA performed on the entire dataset using the log-transformed ratios of samples over the pooled control (tenth channel) and an analysis of the *P* value distribution using paired *t* tests between triplicates of MtzR and MtzS lines. The Pearson correlation between log-transformed fold change in transcript [18] and protein abundances was calculated in R and visualized using the ggplot2 library. For brevity, the gene accession prefix, GL50803, was omitted during further discussions of individual genes.

### Gene set enrichment analysis 

Functional annotation of proteins was performed using Uniprot to assign GO function, subcellular localization, Interpro protein domains, and structure annotations where available. Gene set enrichment analysis (GSEA) was performed on differentially expressed proteins using the DAVID bioinformatics resource [96]. GiardiaDB.org open reading frame identifiers from combined up- and downregulated proteins in each MtzR isolate were converted to gene identifiers using the National Center for Biotechnology Information Batch Entrez tool [97]. Converted gene identifier lists were submitted by isolate to DAVID for GSEA, with GO annotations and Interpro annotations submitted for testing. Gene sets with an EASE score ≤0.2 in at least 1 MtzR line were retained.

### Protein–protein interaction networks

Network analysis was performed by submitting DEP ascensions to the STRING software (v10.5) [98, 99]. Interaction networks were visualized for proteins with medium confidence (0.4), with network edges based on confidence, continuous lines for direct interactions, and indirect interactions with interrupted lines. Clustering was based on a Markov Cluster Algorithm (MCL) inflation default parameter of 3.

### MtzR revertant cell culture

Trophozoites from the lines 106-MtzR and 713-MtzR were cultured in TYI-S33 in the presence of Mtz as above and designated passage 0 (P0). Subsequently, Mtz drug selection was discontinued, and isolates were subcultured twice weekly without Mtz. MtzR revertant cultures were preserved every 4 weeks. IC_50_ values were determined for cells at P8, P16, and P24 relative to susceptible parent isolates as detailed in Section 2.1 in Ansell et al. [18].

### Western blotting of post-translational protein modifications

Adhered trophozoites grown to confluence from all MtzR and MtzS isogenic isolates, as well as 106-MtzR and 713-MtzR P0, P8, and P16 revertant cultures, had protein extracted in 2.5% SDS in 100 mM Tris containing 5 mM Trichostatin A (BioAustralis) and HALT^®^ protease and phosphatase inhibitor (Life Technologies). The protein concentration was determined via BCA assay (Pierce), and then samples were reduced with 15 mM dithiothreitol at 90°C. A total of 15 μg of proteins were resolved on 4–12% Bis-Tris gradient gels (Invitrogen) in 1 × 3-(N-morpholino)propanesulfonic acid running buffer (Invitrogen) and were transferred to nitrocellulose membranes (Sigma). All antibodies were obtained from Cell Signalling Technologies and included antibodies were directed to KAc, K-MMe, phosphorylated tyrosine (pY), the 14–3-3 binding motif (including phosphorylated serine) (14–3-3), and ubiquitin. Anti-H3 and anti-H4 antibodies were obtained from Abcam. Consistent protein loading was verified after transfer using Ponceau S staining (Sigma). Protein–antibody interaction was detected with an horseradish peroxidase (HRP)-conjugated immunoglobulin G secondary antibody using enhanced chemiluminescent reagent (LumiGLO^®^, Cell Signalling Technologies) on a BioRad ChemiDoc MP imaging system with exposure times optimized for each antibody; images were collected up to 200 seconds and selected based on resolution and absence of oversaturation.

### Chemical inhibitors of post-translation protein modification networks

Staurosporine, chaetocin, and TSA were obtained from BioAustralis, and balyculin A was obtained from Sigma. For each of the chemical inhibitors, IC_50_ was calculated as previously determined and detailed in Section 2.1 in Ansell et al. [18] in both MtzS and MtzR lines. All stocks were solubilized in DMSO.

TSA exposure courses were optimized at 1, 2, and 4μM TSA for 8 hours after 48 hours growth prior to exposure and then exposed to 2 μM for 18 hours after 72 hours growth prior to exposure. For both exposure courses, trophozoites were grown without the addition of Mtz in MtzR lines, and only adherent trophozoites were used in exposure time courses. Prior to TSA exposure, nonadhered trophozoites were discarded along with the old media; fresh media added containing either TSA in DMSO or DMSO only (control) was added. Adherence was monitored throughout exposure time courses, with detachment indicating decreasing viability. Trophozoites from exposure experiments had protein extracted and analyzed using anti-KAc as detailed previously, with 10μg of protein run on the gels.

## Availability of supporting data

Proteomic datasets including raw files, mascot search files, and TMT protein ratios can be accessed for free at the European Bioinformatics PRIDE database via ProteomeXchange with identifier PXD007183. Supporting data are also available via the *GigaScience* repository GigaDB [100].

## Additional file

Supplementary Data S1: Protein and peptide identifications, TMT reporter ion ratios and protein quantitation and *P*-value significance. Supplementary for TMT1 (WB MtzS vs MtzR) is presented on the first tab, TMT2 (106 MtzS vs MtzR) is presented on the second tab and TMT3 (713 MtzS vs MtzR) is presented on the third tab. These include GL50803_ identifiers, converted “Entrez Gene IDs” for DAVID bioinformatic functional analyses, the geometric mean for fold change and the T-rest *P*-value of significance. A significant *P*-value (<0.05) has been highlighted in yellow. Proteins above the upregulated fold change cutoff are highlighted in red, while proteins below the fold change for downregulation are highlighted in green.

Supplementary Data S2: Functional annotation for differentially expressed proteins in MtzR lines. Supplementary for TMT1 (WB MtzS vs MtzR) is presented on the first tab, TMT2 (106 MtzS vs MtzR) is presented on the second tab and TMT3 (713 MtzS vs MtzR) is presented on the third tab. Each supplementary tab shows the functional annotation, including for GO, Interpro and SignalP for each of the DEPs which met *P*-value and fold change cutoffs for differential expression in each MtzR lines. A significant *P*-value (<0.05) has been highlighted in yellow. Proteins above the upregulated fold change cutoff are highlighted in red, while proteins below the fold change for downregulation are highlighted in green.

Supplementary Data S3: DAVID functional annotation enrichment and clusters. The six enriched functional clusters are shown, including the MtzR lines in which enrichment was observed, and the GO annotation and/or Interpro domains around which the functional clusters were selected. The *Giardia* identifiers of each of the DEPs involved with the designated Interpro and GO annotations are listed within each of the three *Giardia* MtzR lines.

Supplementary Figure S1: (A) Volcano plots illustrating the dual criteria for differentially expressed proteins. The x-axis represents log fold change with the vertical blue lines indicating 1.3 and 0.77 ratio, while the -log *P* value is plotted on the y-axis with proteins above the red horizontal line indicating significance ≤0.05. Each data point represents a single identified protein. Proteins within the upper and outer quadrants meet both the fold change and *P*-value cut-off, and are therefore considered as differentially expressed. (B) PCA plots of principal component scores plot in the space of the first 3 principal components generated for the whole dataset of log_2_ ratios to the pooled control (label 131) with each distribution for sample groups highlighted. All channels relevant to MtzR and MtzS ratio calculated are highlighted in the plots, with triplicate channels for MtzS (DMSO Control) lines are shown in each of the three plots are shown in green, while the channels containing the MtzR replicates are highlighted in purple (WB-MtzR), red (106-MtzR) and blue (713-MtzR) (C) *P*-value histograms showing the distribution of *P*-values from the paired t-tests comparing the MtzS samples respectively to MtzR. The *P*-value histograms have a peak corresponding to a larger number of low *P*-values, which is indicative of a real underlying effect; a random or noisy dataset is expected to generate a uniform distribution of *P*-values and hence a flat histogram.

Supplementary Figure S2: Protein-RNA log^2^ fold change correlations plots by genotype for all proteins quantified by TMT label ratios. Fold change was derived from ratios of MtzR replicated over their MtzS line, and then log^2^ transformed. The corresponding RNA log^2^ fold change was derived from transcript expression data from Ansell *et al* [18]. Correlation between protein and RNA abundance fold changes were calculated at r^2^ = 0.154 for WB, r^2^ = 0.105 for WB and r^2^ = 0.187 for WB (*P* < 0.01) for genes identified in both datasets.

Supplementary Figure S3: Proportional Venn diagrams showing the number of differentially expressed proteins relative to protein identifications across the three TMT experiments to compare MtzS and MtzR isolates. Proportional Venn diagrams demonstrate that the low overlap between differentially expressed proteins between lines (Fig. 1), is not due to discrepancies in identifications between experiments.

Supplementary Figure S4: Protein interactome network for DEPs in WB MtzR (Top), 106 MtzR (Middle) and 713 MtzR (Bottom). Using the STRING software, proteins are represented with nodes and the interactions with continuous lines to represent direct interactions (physical), while indirect ones (functional) are presented by interrupted lines. Thickness of the line is to represent the strength of the data support of interaction evidence between network nodes. Colouring is performed according to MCL clustering. The coloured background and overarching functional annotations have been added to highlight protein-protein interaction networks of multiple nodes with direct interactions among DEPs in the MtzR lines.

Supplementary Figure S5: Total protein lysate from trophozoites (10μg) from WB, 713 and 106 wild type lines was probed with antibodies against the H3 variant (top) and H4 variant (bottom), as well as probed with antibodies again acetylated lysine (KAc) and mono-methylated lysine (K-MMe). Blots have been cropped to 25–10kDa. The H3 variant (∼17kDa) and the H4 variant (∼11kDa) bands correspond to the position of the prominent modified protein bands on KAc and KMme blots, mostly likely the acetylation and methylation histone marks on the H3 and H4 variants.

Supplementary Figure S6: Dose-response curves for MtzS lines (dark colors, solid line) and MtzR lines (light colors, dotted lines) for chemical inhibitors of protein post-translational modification networks. Experiments performed in biological triplicate. Error bars represent ± 1 standard deviation. Table below dose response curves show the IC_50_ concentration for each line, and the resistance factor is calculated for MtzR lines relative to their MtzS parent. Trichostatin A (grey fill) had a significantly increased IC_50_ in all MtzR lines relative to their MtzS parents. Due to the high IC_50_ in Chaetocin, and the limits of solubility, it was difficult to plot the dose response curve for this compound and the IC_50_ was approximately estimated in some lines.

Supplementary Figure S7: Total protein lysate (10μg) from trophozoites exposed for 8 hours to 1, 2 and 4μM TSA, with control flasks exposed to the same volume DMSO as used as a vehicle for TSA. Protein from WB, 713 and 106 MtzS and MtzR lines probed with antibodies again acetylated lysine (KAc) (below). Protein loading was verified after transfer using Ponceau S staining (upper). At 8 hours, MtzS isolates are beginning to show hyper-acetylation of H3 and H4 variants, which is not observed in MtzR lines. Based on this optimization experiment, 2μM of TSA was chosen as a concentration for a longer TSA exposure time course in MtzR and MtzS lines.

Supplementary Figure S8: Light microscope images of trophozoite in MtzS and MtzR lines adhered to the flat-sided 10 mL tubes at 72hrs confluence prior to passage. Wild-type MtzS lines have reached complete confluence, while MyzR lines have varying degrees of reduced confluence and adherence as compared to MtzS parents.

Supplementary Figure S9: Growth improvements in MtzR lines during *in vitro* culture upon discontinuation of drug selection. Isolates were subcultured twice weekly to ensure consistent culture conditions, media availability and time in culture between the 2 lines (106-MtzR, 713-MtzR). Left axis shows the fold increase relative to MtzS parents in seed volume required for MtzR lines to compensates for lower growth rates/growth defects, while the right axis reflects the % confluence of adhered trophozoites averaged across the two subculture passages for that week. During Mtz selection in week 0, lower confluence and higher seed values were required. Upon discontinuation of drug selection, confluence and growth improved, and lower seed volumes required.

## Abbreviations

BCA: Bicinchoninic acid; DEP, differentially expressed protein; DMSO: dimethylsulfoxide; EGF: Extracellular growth factor; gFAELO, fatty acid elongase 1; gLCFACL, long chain fatty acid CoA ligase; GO, gene ontology; gPI4P5K, phosphatidylinositol-4-phosphate 5-kinase; gPLTATPase IIB, phospholipid-transporting ATPase IIB; gPSS, phosphatidylserine synthase; GSEA, gene set enrichment analysis; HAT, histone acetyltransferase; HCMP, high cysteine membrane protein; HDAC, histone deacetylase; IEC, intestinal epithelial cell; KAc, acetylated lysine; KAT, lysine acetyltransferase; KDa, kilodalton; KDAC, lysine deacetylase; K-MMe, mono-methylated lysine; Mtz, metronidazole; NAD: Nicotinamide adenine dinucleotide; NADPH: Nicotinamide dinucleotide phosphate; nanoLC-MS/MS, nanoflow liquid chromatography tandem mass spectrometry; NR, nitroreductase; PCA, principal component analysis; PFOR, pyruvate ferrodoxin oxidoreductase; PITPα, PI transfer protein alpha isoform; PP2A, protein phosphatase type 2A; pY, phosphorylated tyrosine; SDS: sodium dodecyl sulfate; SPE, solid phase extraction; STRING: Search Tool for the Retrieval of Interacting Genes; TMT, tandem mass tags; TSA, trichostatin A; VSP, variant-specific surface protein.

## Competing interests

The authors declare that they have no competing interests.

## Funding

This work, including the efforts of A.J. and M.M., was funded by Australian Research Council (ARC) (LP120200122). S.E., B.A., L.B., and A.J. are supported by the Victorian State Government Operational Infrastructure Support and Australian Government National Health and Medical Research Council Independent Research Institute Infrastructure Support Scheme. A.J. is also supported by a NHMRC Career Development Fellowship (APP1126395). S.E. and this research are also supported by a Jack Brockhoff Foundation Early Career Grant (ID 4184). The funders had no role in study design, data collection and interpretation, or the decision to submit the work for publication.

## Author contributions

B.A., A.J., and L.B. designed the experiment. B.A. and L.B. generated the samples. S.E. and M.M. processed the samples for proteomics and performed the MS. S.E. analyzed the data. S.E., B.A., A.J., M.M., P.H., M.J.M., and S.S wrote the manuscript.

## Supplementary data

Supplementary data are available at *GIGSCI* online.

GIGA-D-17-00213.pdf

GIGA-D-17-00213_R1.pdf

Response_to_Reviewer_Comments_Original_Submission.pdf

Reviewer_1_Report_(Original_Submission) -- Carmen Faso 05 Sep 2017 Reviewed

Reviewer_1_Report_(Revision_1) -- Carmen Faso 22 Jan 2018 Reviewed

Reviewer_2_Report_(Original_Submission) -- Andrea Rópolo 14 Sep 2017 Reviewed

Reviewer_2_Report_(Revision_1) -- Andrea Rópolo 05 Feb 2018 Reviewed

Reviewer_3_Report_(Original_Submission) -- Guadalupe Ortega-Pierres 02 Oct 2017 Reviewed

Reviewer_3_Report_(Revision_1) -- Guadalupe Ortega-Pierres 06 Feb 2018 Reviewed

Supplemental material
