# Supplementary material for: Differential protein expression and post-translational modifications in metronidazole-resistant Giardia duodenalis
Source: Gigascience. 2018 Mar 13;7(4):giy024. doi: 10.1093/gigascience/giy024 (PMC5913674; doi:10.1093/gigascience/giy024)
Supplement: GIGA-D-17-00213_R1.pdf [file giy024_giga-d-17-00213_r1.pdf]

GigaScience

Differential protein expression and post-translational modifications in Metronidazole-resistant Giardia duodenalis

--Manuscript Draft--

**Manuscript Number:**	GIGA-D-17-00213R1
**Full Title:**	Differential protein expression and post-translational modifications in Metronidazole-resistant Giardia duodenalis
**Article Type:**	Research
**Funding Information:**	Australian Research Council (LP120200122) — Prof Malcom John McConville, A/Prof Aaron Richard Jex<br>National Health and Medical Research Council (APP1126395) — A/Prof Aaron Richard Jex<br>Jack Brockhoff Foundation Early Career Grant (4184) — Dr Samantha Jane Emery
**Abstract:**	Background: Metronidazole (Mtz) is the frontline drug treatment for multiple anaerobic pathogens, including the gastrointestinal protist, Giardia duodenalis. However, treatment failure is common and linked to in vivo drug resistance. In Giardia, in vitro drug-resistant lines allow controlled experimental interrogation of resistance mechanisms in isogenic cultures. However, resistance-associated changes are inconsistent between lines, phenotypic data are incomplete, and resistance is rarely genetically fixed, highlighted by reversion to sensitivity after drug selection ceases, or via passage through the life cycle. Comprehensive quantitative approaches are required to resolve isolate variability, fully define Mtz resistance phenotypes, and explore the role of post-translational modifications (PTMs) therein.<br>Findings: We performed quantitative proteomics to describe differentially expressed proteins (DEPs) in three seminal Mtz resistant (MtzR) lines compared to their isogenic, Mtz-susceptible, parental line. We also probed changes in PTMs including protein acetylation, methylation, ubiquitination and phosphorylation via immunoblotting. We quantified over 1000 proteins in each genotype, recording substantial genotypic variation in DEPs between isotypes. Our data confirms substantial changes in the antioxidant network, glycolysis and electron transport, and indicates links between protein acetylation and Mtz resistance, including cross-resistance to deacetylase inhibitor Trichostatin A (TSA) in Mtz resistant lines. Finally, we performed the first controlled, longitudinal study of Mtz resistance stability, monitoring lines after cessation of drug selection, revealing isolate-dependent phenotypic plasticity.<br>Conclusions: Our data demonstrate understanding of Mtz resistance must be broadened to post-transcriptional and post-translational responses, and that Mtz resistance is polygenic, driven by isolate-dependent variation, and is correlated with changes in protein acetylation networks.
**Corresponding Author:**	Samantha Emery<br><br>AUSTRALIA
**Corresponding Author Secondary Information:**	
**Corresponding Author's Institution:**	
**Corresponding Author's Secondary Institution:**	
**First Author:**	Samantha Jane Emery, PhD
**First Author Secondary Information:**	
**Order of Authors:**	Samantha Jane Emery, PhD
	Louise Baker, PhD

Powered by Editorial Manager® and ProduXion Manager® from Aries Systems Corporation

	Brendan Robert Edward Ansell, PhD
	Mehdi Mirzaei, PhD
	Paul Andrew Haynes, PhD
	Malcom John McConville, PhD
	Staffan Gunnar Svärd, PhD
	Aaron Richard Jex, PhD
**Order of Authors Secondary Information:**	
**Response to Reviewers:**	Nicole Nigoy, Ph.D.<br>Gigascience<br><br>Dear Nicole,<br>We thank the reviewers for their insightful comments and suggestions regarding our manuscript. We have incorporated these comments and additional experiments where possible for us to do so, and we believe the manuscript is greatly improved. Specifically, we have added additional experiments using broad-spectrum PTM inhibitors to further characterize the role of PTMs in Mtz resistance, which strengthened our results regarding the relationship between acetylation and Mtz resistance. We have also added five new supplementary figures, including DEPs as protein-protein interaction networks, and western blots of Anti-H3 and Anti-H4 to confirm the identities of protein bands on Anti-KAc and Anti-MMe blots. Responses to specific reviewer comments can be found below.<br><br>We hope that these additional comments and experiments address our reviewer's concerns and thank you for your further consideration.<br><br>On behalf of all co-authors<br><br>Dr Samantha Emery<br>Walter and Eliza Hall Institute<br><br><br>Specific reviewer comments<br><br>Reviewer #1:<br>R1-Q1: Validating PTM-related data using chemical inhibitors of protein acetylation, methylation and phosphorylation: We thank the reviewer for their suggestion, which we have attempted to address in our revision. The PTM networks of Giardia are poorly studied, and inhibitors of these networks have not been widely screened such that the targeted and specific chemical inhibition of Giardia PTM enzymes is not currently possible. We have focused our additional work on four broad inhibitors, Trichostatin A (TSA: acetylation), Chaetocin (Methylation) and Staurosporine and Calyculin A (phosphorylation), of which all but Chaetocin have known activity in Giardia.<br>To test our findings, we determined IC50 dose response curves in MtzS and MtzR lines of all 3 isolates, finding that each MtzR line has significant cross-resistance to Trichostatin A, a broad-spectrum deacetylase inhibitor. This further supports that lysine acetylation has strong links to Mtz resistance, as per our paper's conclusions. We then exposed all 6 lines to 1, 2 and 4uM TSA for 8 hours for optimization, and selected 2uM TSA for a subsequent 18 hour exposure. These were immunoblotted with anti-KAc. MTZs lines showed hyperacetylaton of histones 8 hours and increased KAc of multiple other protein features following TSA exposure for 16 hours. However, MtzR lines showed decreases in KAc, which points to widespread changes in activity of KAc network enzymes in MtzR lines, not just deacetylases.<br><br>The manuscript has been amended to include these experiments:<br>The methods and results sections each now include the section "Chemical Inhibitors of post-translation protein modification networks". We have added an additional figure to the manuscript (Figure 6), as well as two new Supplementary Figures (Supplementary Figure S6, Supplementary Figure S7). There have also be changes to the abstracts, discussion and results to incorporate these additional experiments into the pre-existing sections of the manuscript.

Powered by Editorial Manager® and ProduXion Manager® from Aries Systems Corporation

R1-Q2: PTM protein band identification. We intend to identify the PTM substrates that change their modification state with Mtz exposure and resistance through IAP experiments in a subsequent study. However, as none of these networks have been investigated in Giardia, they warrant separate and dedicated analysis. IAP pulldowns of modified peptides is a significant undertaking, involving considerable costs and time, and, in our opinion, it is beyond the scope of this experiment to perform pull downs and quantitative mass spectrometry for four networks in six lines. Our primary finding with regards to PTMs is, at present, a general finding, namely that there is clear remodeling of PTM networks (demonstrated by western blot) and that there is a link between lysine acetylation and MtzR, confirmed here based on Trichostatin exposures.

Reviewer #2:
R#2-Q1: Presentation of our proteomic analysis. Our manuscript has been written to highlight that while the DEPs in each line are different, there are overarching similarities in the type of functions of these proteins (e.g., ABC transporters, oxidoreductases/electron transport proteins, membrane proteins, cell signalling), which points to overlapping mechanisms underpinning MtzR. While we respect the reviewer's opinion, in our opinion, this is the best way to present this data.

R#2-Q2 – VSPs - turnover. Although VSPs do spontaneously change during growth in Giardia, certain VSPs confer higher tolerance to bile, or have been hypothesized to increase tolerance to oxidative stress (Ma'ayeh et al, 2015, Int J Parasitol). Previous transcriptomics of Mtz resistance (Ansell et al, 2017, Front. Microbiol.; Muller et al, 2007, J Antimicrob Chemother) have reported drastic changes to VSP expression associated with resistance, including specific variants. Given that VSPs are post-transcriptionally regulated (Prucca et al, 2008, Nature) and are functionally enriched as a cluster in all three lines, our protein expression data is an important complement to transcriptomic data and as such it has been discussed. That we do not see convergence of different MtzR lines to similar variants suggests either that these VSP changes are stochastic turn-over (as per the reviewer's suggestion) or that certain variants confer greater MtzR tolerance. We feel recording this observation is important, but have modified its description in the manuscript to reflect the reviewer's concerns (please see R#2-Q4 below).

R#2-Q3 –Cloning lines for VSP turnover analysis: Given the historical precedence of these lines in Mtz resistance literature, we are not able to clone these isolates out and diverge them from observations in previously-generated datasets.

R#2-Q4 - In the discussion section the lines 1-10 of page 19 are absolutely speculative, considering that the authors do not analyze the VSP turnover in the MtzR lines. We have removed this section from the discussion, and we have amended the section of the discussion regarding VSP turnover and differential expression.

R#2-Q5 - PTM analysis is very preliminary and needs more experiments. Please see R1-Q1 response and additional experimental listed above.

R#2-Q6 - Ponceau vs an internal control, like anti-tubulin, to validate each blot. We have used Ponceau because (1) it is difficult to source verified antibodies for Giardia to use a loading control and (2) with six lines with very different proteomic profiles it is even more difficult to identify a consistent loading control with equal expression across the six lines. Tubulin, for example, would not be an appropriate choice as it is very close to the DE cutoffs in several line). Therefore, we felt Ponceau, which has been used previously for Westerns in Giardia (Sonda et al, 2010, Mol Microbiol), was a reliable alternative.

R#2-Q7 - This experiment should be complemented with IPP using antibodies and mass spectrometry. Characterising protein substrates of PTM networks, including in context to Mtz resistance is indeed the next step in our work, however we feel that these currently uncharacterized networks warrant separate, dedicated analysis. Please see R1-Q2 in regard to identification of PTM modified proteins and IAP experiments.

R#2-Q8: Observations on acetylated or methylated histones. We have now performed additional western blots using Anti-H3 and Anti-H4 side by side with Anti-KAc and Anti-

Powered by Editorial Manager® and ProduXion Manager® from Aries Systems Corporation

MMe to show that anti-H3/H4 align with the modified bands on the corresponding blots in the wild type isolates (WB, 106 and 713). These have been added in Supplementary Figure S5 and the manuscript altered to reflect the addition of the figure as follows: "Both KAc and K-MMe modification detection for histone variants is consistent with previously detected histone modification states via Western blot in Giardia [54], and subsequent immunoblotting with Anti-H3 and Anti-H4 showed that the modified protein bands at ~17 and ~11KDa in KAc and KMMe blots aligned with antibodies for these histone variants (Supplementary Figure 5)."

R#2-Q9 – Analyzing the expression of HAT, HMT, phosphatases using qRT-PCR. Complete transcriptomic analyses have already been conducted and analysed from the same cell cultures (from total RNA co-purified from the same source cells as all proteins evaluated here for mass spec proteomics) and published earlier this year (Ansell et al, 2017, Front Microbiol). There is good agreement between RNA and protein data (Supplementary Figure S2), and as such the expression of KATs and KDACS were discussed on pg21. The RNAseq data provided in Ansell et al (2017), was internally controlled at publication by qRT-PCR and does not need to be repeated here as we are working from the same cell pellets. We have revised the manuscript to make this clear.

R#2-Q10 – Eliminate discussion section lane 1-22 of page 21. More experiments related with the analysis of the expression of KAT and K/HDAC should be performed. Given we have reasonable correlation between Protein-RNA log2 fold change in the two experiments for genes identified in both datasets (Supplementary Figure S2), we believe it is valid to intersect the protein dataset with the transcript data from these lines. Lines 1-22 reflect a robust discussion of transcript and protein results of KAc network enzymes, not just KAT expression in the transcript data, and we believe it is important to keep this discussion of the wider KAc network in the manuscript.

R#2-Q11 – Why is the PTM pattern at P0 is different from the one in MtzR lines (figure5) if they were growth in the presence of Mtz? There were not changes, but due to increases in the intensity of the histone variant bands (H3 and H4 at ~17 and ~11kDa) and the prominent band at ~30kDa in MMe profiles we had to lower chemiluminescence exposure time to prevent oversaturation of the major bands thus allowing comparisons of profiles between passage timepoints. This resulted in the loss of some low intensity bands. This information on exposure times has been added to the Figure legend for Figure 7 (formerly figure 6) and the methods.

R#2-Q12 - Results found using WB are speculative, and in some cases, like in figure 6 i and ii, there is an increase in the general K-MMe pattern at P8 in 106 that then decrease. Again, it is necessary the use of an anti-tubulin antibody. It would be interesting to include the WB at P24. The increase at P8 and decrease at P16 fits the alternative profile of loss of Mtz resistance at P8 and then gain at P16 in resistance, which is different to the resistance profile and WB profile in 716 which has decreases in resistance at P8 and P16.

R#2-Q13 - All the results related to histone modifications should be avoided. Histone modifications have been addressed above (see R#2-Q6) with the additional Anti-H3 and Anti-H4 Western blots in Supplementary Figure S5.

R#2-Q14 - The conclusions in page 13 line 58 and page 14 (lines 1-4) are speculative. The statement the reviewer is referring to proposed as a hypothesis preceded by the statement "it is possible…". We believe that the observation of lower abundance of ventral disc proteins and lower adherence in these lines is interesting (further reinforced in the new supplementary figure S8, which shows reduced adherence in WB and 713 MtzR lines). We have adjusted our revised manuscript to make more clearly that we are proposing this as a potential hypothesis, not a conclusion. We agree that our data can't test this hypothesis at present.

R#2-Q15 - Sir2 10708 is a hypothetical protein that should be named: putative Sir2. While the 10708 is a hypothetical protein in GiardiaDB, its sequence homology and function was investigated as a Sir2 gene late last year (Carranza et al, 2016 Int J Biochem Cell Biol) and our nomenclature reflects this.

Powered by Editorial Manager® and ProduXion Manager® from Aries Systems Corporation

	Reviewer 3:<br>R#3-Q1: Analyze the interactome (the differential protein-protein interaction networks) using DEPs. In order to analyses protein-protein interactions, DEPs from the three lines were submitted to STRING (Search Tool for the Retrieval of Interacting Genes) software (v10.5) (http://string.db.org) . The results of this have been added as Supplementary Figure 4 and networks interactions largely complement the functional enrichment analyses as investigated using DAVID in the manuscript. The methods have been amended to include the reference to STRING, and the supplementary figure has been integrated into the results of the text.<br><br>R#3-Q2 – qPCRs to assess comparisons (figure FS2) of protein expression and RNA transcript levels of the respective genes from the same strains (Ansell et al., 2017). Please see R#2-Q7 for our response regarding qPCR-based testing of our RNA-seq data. For our proteomic datasets, we have followed statistical techniques previously employed for TMT proteomics in Giardia (Emery et al, 2016, Sci Reps) and recognized in the broader discipline of isobaric labelling (Mahoney et al, 2011; Pascovici et al, Proteomics, 2016). We have used robust statistical cutoffs to analyse these data, including 1) setting both fold change and p-value cutoffs for differential expression, 2) using unsupervised multivariate Principal Component Analysis (PCA) and 3) analysing the p-value distribution using the paired t-tests between triplicates of MtzS and MtzR fold changes. All of these are represented in Supplementary Figure 1. We and others have shown (Pascovici et al, Proteomics, 2016) that this approach is more reliable than targeting a small subset of proteins by western blot.<br><br>R#3-Q3 – Identify acetylated, methylated or phosphorylated substrates in MtzR lines using specific antibody pulldowns. This is indeed the next step in the work, however, our primary finding in relation to PTMs, is that lysine acetylation is closely associated with MtzR phenotype. This hypothesis is further supported by inhibitor studies provided in our revised submission (please see R#1-Q1). Regarding direct IAP pulldowns, please see our response to R#1-27.<br><br>R#3/-Q4 - Explain the isolate variation in Mtz tolerance in the absence of drug. We believe the author is referring to the different IC50 profiles of 713 and 106 through P8, P16 and P24 after cessation of drug selection. In regard to this, we have provided some hypotheses as to why these isolates tolerate Mtz differently after drug selection, in particular the possibility of stable resistance traits in unresolved chromosomal aberrations and/or nonsense mutations in key Mtz resistance genes (on pg 22 of the manuscript discussion). However this is a novel and unexpected result, and will require more thorough and extensive investigation in follow-up experiments beyond the scope of this study.
**Additional Information:**	
**Question**	**Response**
Are you submitting this manuscript to a special series or article collection?	No
**Experimental design and statistics**<br><br>Full details of the experimental design and statistical methods used should be given in the Methods section, as detailed in our Minimum Standards Reporting Checklist. Information essential to interpreting the data presented should be made available in the figure legends.<br><br>Have you included all the information requested in your manuscript?	Yes

Powered by Editorial Manager® and ProduXion Manager® from Aries Systems Corporation

**Resources**<br><br>A description of all resources used, including antibodies, cell lines, animals and software tools, with enough information to allow them to be uniquely identified, should be included in the Methods section. Authors are strongly encouraged to cite Research Resource Identifiers (RRIDs) for antibodies, model organisms and tools, where possible.<br><br>Have you included the information requested as detailed in our Minimum Standards Reporting Checklist?	Yes
**Availability of data and materials**<br><br>All datasets and code on which the conclusions of the paper rely must be either included in your submission or deposited in publicly available repositories (where available and ethically appropriate), referencing such data using a unique identifier in the references and in the “Availability of Data and Materials” section of your manuscript.<br><br>Have you have met the above requirement as detailed in our Minimum Standards Reporting Checklist?	Yes

Manuscript Click here to download Manuscript Emery6-3-18.docx

Differential protein expression and post-translational modifications in Metronidazole-resistant *Giardia duodenalis*

Samantha J. Emery[1], Louise Baker[2], Brendan R.E. Ansell[2], Mehdi Mirzaei[3,4], Paul A. Haynes[3], Malcom J. McConville[5], Staffan G. Svärd[6], Aaron R. Jex[1,2]

[1] Population Health and Immunity Division, Walter and Eliza Hall Institute of Medical Research, Melbourne, VIC, Australia
[2] Faculty of Veterinary and Agricultural Sciences, The University of Melbourne, Melbourne, VIC, Australia
[3] Chemistry and Biomolecular Sciences, Faculty of Science, Macquarie University, North Ryde, NSW, Australia
[3] Australian Proteome Analysis Facility, Macquarie University, North Ryde, NSW, Australia
[5] Bio21 Molecular Science and Biotechnology Institute, The University of Melbourne, Melbourne, VIC, Australia
[6] Department of Cell and Molecular Biology, Uppsala University, Uppsala, Sweden

Author Emails:

SJE: emery.s@wehi.edu.au
LB: baker.l@wehi.edu.au
BREA: ansell.b@wehi.edu.au
MM: mehdi.miraei@mq.edu.au
PAH: paul.haynes@mq.edu.au
MJM: Malcomm@unimelb.edu.au
SGS: staffan.svard@icm.uu.se
ARJ: jex.a@wehi.edu.au

Corresponding Author:

Dr Samantha Jane Emery
Population Health and Immunity Division, Walter and Eliza Hall Institute of Medical Research
Parkville, Victoria, 3052
Australia
Email: emery.s@wehi.edu.au
Phone: +61-3-9345-2656

PRIDE Login Details:
Dataset Identifier: PXD007183
Username: reviewer37286@ebi.ac.uk
Password: cpXlsOIK

Abstract:

Background: Metronidazole (Mtz) is the frontline drug treatment for multiple anaerobic pathogens, including the gastrointestinal protist, *Giardia duodenalis*. However, treatment failure is common and linked to *in vivo* drug resistance. In *Giardia*, *in vitro* drug-resistant lines allow controlled experimental interrogation of resistance mechanisms in isogenic cultures. However, resistance-associated changes are inconsistent between lines, phenotypic data are incomplete, and resistance is rarely genetically fixed, highlighted by reversion to sensitivity after drug selection ceases, or via passage through the life cycle. Comprehensive quantitative approaches are required to resolve isolate variability, fully define Mtz resistance phenotypes, and explore the role of post-translational modifications (PTMs) therein.

Findings: We performed quantitative proteomics to describe differentially expressed proteins (DEPs) in three seminal Mtz resistant (MtzR) lines compared to their isogenic, Mtz-susceptible, parental line. We also probed changes in PTMs including protein acetylation, methylation, ubiquitination and phosphorylation via immunoblotting. We quantified over 1000 proteins in each genotype, recording substantial genotypic variation in DEPs between isotypes. Our data confirms substantial changes in the antioxidant network, glycolysis and electron transport, and indicates links between protein acetylation and Mtz resistance, including cross-resistance to deacetylase inhibitor Trichostatin A (TSA) in Mtz resistant lines. Finally, we performed the first controlled, longitudinal study of Mtz resistance stability, monitoring lines after cessation of drug selection, revealing isolate-dependent phenotypic plasticity.

Conclusions: Our data demonstrate understanding of Mtz resistance must be broadened to post-transcriptional and post-translational responses, and that Mtz resistance is polygenic, driven by isolate-dependent variation, and is correlated with changes in protein acetylation networks.

Keywords: *Giardia duodenalis;* quantitative proteomics; Metronidazole; drug resistance; protein posttranslational modifications

Background:

Nitroheterocyclics (e.g metronidazole (Mtz), nitazoxanide and furazolidone) include 'redox-active' pro-drugs which cross the cell membrane via passive diffusion and are enzymatically reduced to cytotoxic intermediates that oxidise biomolecules. This occurs specifically within highly reducing intracellular environments of microaerophilic protists (*Giardia duodenalis*, *Trichomononas vaginalis* and *Entameoba histolytica*) as well as anaerobic bacteria (*Helicobacter pylori*, *Clostridium difficile* and *Bacteroides fragilis*) [1, 2]. By contrast, Mtz has low toxicity in aerobic cells, where dO_2 re-oxidizes reduced Mtz to the pro-drug form, termed futile cycling. The specificity of nitroheterocyclic toxicity for low dissolved oxygen biochemistry makes this drug class the chemotherapeutic backbone against multiple bacterial and protozoan pathogens, however, drug resistance within this compound class is collectively widespread across species [2-4].

G. duodenalis (syn. *G. lamblia, G. intestinalis*) is a parasitic protist responsible for between 200-300 million cases of diarrheal disease (giardiasis) annually [5]. This microaerophile exhibits fermentative glycolysis coupled to an antioxidant system that maintains an electron-rich (i.e., highly reduced) intracellular environment. Chemotherapeutic treatments for giardiasis are limited, but remain the primary treatment option targeting the trophozoite; the infective life-stage that attaches to the gastro-epithelial lining of the proximal small intestine [6]. Nitroheterocyclics, in particular Mtz, remain the predominant class against these parasites. However the efficacy of frontline Mtz treatment ranges between 73–100% in *Giardia* [7], with clinical resistance confirmed [7, 8] and increasing in incidence [9].

Mtz interacts with oxidoreductase enzymes in *Giardia*, which include glycolytic and antioxidant enzymes, but is also influenced by enzymes contributing to the reduction potentials through electron transport, cofactor abundance and flavin metabolism. Changed activity or expression of these enzymes correlate with resistance, with changes leading to decreased activation or increased detoxification of Mtz known as passive and active resistance mechanisms, respectively [2]. In *Giardia*, the down-regulation or reduced activity of pyruvate:ferredoxin oxidoreductase (PFOR) is a centrally recognised passive resistance mechanism [10-14]. However, transcriptional studies suggest that exposure to Mtz elicits genome-wide changes in *Giardia* [15], including within wider glycolytic and redox systems. Notably, down-regulation of thioredoxin reductase [10], which links thiol metabolism to thioredoxins and peroxiredoxins in the antioxidant system, is a passive resistance mechanism

that can limit activation of Mtz, albeit at presumed costs to collateral antioxidant systems. Furthermore, the role of the two nitroreductases (NR) in *Giardia* have been implicated in Mtz resistance, with NR-1 and NR-2 activating and detoxifying MtzR, respectively, and are active (NR-2) and passive (NR-1) resistance mechanisms. NR-1 transcript levels are reduced in Mtz resistant lines [16-18] and the enzyme is increasingly recognized as a PFOR-independent mechanism of passive resistance. Drug resistant lines also exhibit differential transcription protein chaperones, thiol-cycling and stress response genes [16], as well as DNA repair mechanisms transcriptional regulators [19, 20].

Collectively, evidence suggests that Mtz resistance is a complex polygenic phenotype (reviewed by [2]). Namely, divergent changes in transcript abundance between genetically similar Mtz-resistant *Giardia* [10, 19] and laboratory lines [15, 18] suggest multiple Mtz-resistant molecular phenotypes. Further, the interaction of transcriptional expression, enzyme activity and, recently, non-synonymous mutations [18] remain to be understood in key enzymes. Phenotypic aspects including infectivity and fitness also differ in lines of different genetic background selected for Mtz-resistance *in vivo* and *in vitro* [14]. Plasticity in the resistance phenotype during encystation [19] or when drug selection is discontinued [21] further suggests reversible or inducible transcriptional regulation. Transcriptional plasticity has been linked to Sir2 NAD-dependent protein deacetylases (sirtuins) [2, 19] and may indicate a role for reversible protein modifications in resistance phenotypes. RNA transcription and control of gene expression in *Giardia* [22-24] suggest an important role for post-transcriptional and post-translation regulation, and global description of protein expression is a key, missing link in Mtz resistance research. In light of this, we undertook detailed, quantitative proteomic analyses in Mtz-resistant and –susceptible *Giardia* lines to identify differentially expressed proteins. This marks, to our knowledge, the first such analysis of Mtz resistance in any parasitic pathogen. This work was conducted in three genetically distinct *Giardia* cell culture isolates that each have been heavily characterized in the literature [25-27] and have shaped the foundational understanding of Mtz resistance in the genus [10, 14, 28]. Moreover, we examine dynamic changes in a wide range of post-translational protein modifications in Mtz-resistant and -susceptible and isogenic isolates, and in the latter after several months of drug free passage.

Data Description:

Giardia duodenalis Mtz resistant (MtzR) and Mtz susceptible (MtzS) lines were previously generated at the Queensland Institute of Medical Research (QIMR) vis long-term sublethal exposure to Mtz in *in vitro* culture. All lines are the Assemblage A genotype, and include the genome reference genotype WB (ATCC 50803), and have been extensively characterised in the literature in the context of Mtz resistance (reviewed in [2]. *In vitro* culture for the three genotypes and drug selection for their resistant, isotype lines (Table 1) was continued in this study, and protein was extracted from adherent, viable trophozoites. Protein was prepared for quantitative proteomics via Tandem Mass Tag (TMT) isobaric labelling to establish fold change between each MtzR lines compared to their drug-susceptible parent lines. A total of three TMT experiments were performed, one for each genotype and its respective isotype and biological triplicates, and each TMT experiment and its fractions was analysed using high-resolution mass spectrometry on a Q-Exactive mass spectrometer (Thermo), and ratios of TMT labels detected in each Mtz resistant to Mtz susceptible replicates was calculated using Proteome Discoverer software v1.3 (Thermo). The mass spectrometry raw data files, database search results and TMT ratios have all been deposited and can be accessed for free via the ProteomeXchange Consortium [29] via the PRIDE partner repository with the dataset identifier PXD007183.

Divergence between differentially expressed proteins in MtzR isotypes led us to consider the potential of genotypic variation, transcriptional plasticity and reversible protein modifications in Mtz resistance. Immunoblotting was performed for acetylation, methylation, ubiquitination and phosphorylation for the three genotypes and isotype lines, as well as for isotype lines monitored after cessation of drug selection every four weeks for up to twelve weeks. Isotype lines maintained without Mtz were also monitored for reversion to Mtz sensitivity, and IC_{50} calculated at 4, 8 and 12 weeks after cessation of drug selection to observe resistance phenotype stability. Chemical inhibitors of protein acetylation, methylation and phosphorylation were also used to further probe post-translational responses, with the IC_{50} calculated for PTM inhibitors in the six lines, which was further complemented by immunoblotting performed of protein extracted from trophozoites exposed to chemical inhibitors.

Results:

Cell Culture of MtzR lines: All three MtzR lines were generated at similar times at the Queensland Institute of Medical Research (QIMR) via long-term exposure to sub-lethal Mtz [30, 31], with intermittent drug treatment and ultraviolet radiation (WB and 713) also used to further induce resistance [31]. WB-M3, BRIS/83/HEPU/106-2ID10 and BRIS/83/HEPU/713-M3 were further explored in subsequent studies [2, 10-12, 14, 16, 32, 33]. The resistant lines used in the current study were selected in the presence of 30 µM Mtz [19], and exhibited significantly higher Mtz IC_{50} values than their respective parent lines, indicating of increased drug tolerance (Table 1).

Quantitative Proteomics: The complete datasets for each isogenic pair, including protein identification and quantitation results as well as label ratios and statistical test results can be found in Supplementary Information S1. Peptide-to-spectrum matching was performed for all three isolates using the A1 sub-assemblage genome (WB C6, ATCC 50803). This reference has few single nucleotide polymorphisms (SNPs) relative to other sequences thus far [34], and has been previously utilised as a database for proteomic analysis of all three isolates with no significant differences in peptide identifications [35]. A non-redundant total of 1571 proteins was identified across all the TMT 10plexes analysed, with 1220, 1126 and 1060 proteins identified in 10plex 1-3, respectively (Table 2).

To quantify protein abundance between drug resistant (MtzR) and susceptible (MtzS) isolates, nine ratios were calculated with each MtzR replicate over all three MtzS replicates, and the geometric mean calculated as a measure of fold change. Reporter ion intensity for each protein was calculated using the pooled control as a common denominator for each TMT channel for normalisation, and was analysed statistically via a one-sample t-test between treatments. Differential expression was contingent on proteins meeting both fold change and p-value cutoffs, as previously described for isobaric label quantitation [36, 37] and as depicted in Supplementary Figure 1, Panel A. PCA analysis of each of the three 10plexes indicated good separation between MtzS parent isolates and their isogenic MtzR lines (Supplementary Figure 1, Panel B), with control MtzS replicates clustering together tightly in all three isolates. Clustering of MtzR lines in the PCA indicated that all three MtzR lines were more variable between replicates than MtzS isogenic parents. Nonetheless, analysis of p-value distribution revealed a an inverse exponential distribution (Supplementary Figure 1, Panel C), consistent with the existence of an underlying signal of differential expression between MtzS and MtzR populations in all three isolates [38].

Differentially Expressed Proteins in MtzR lines: A non-redundant total of 443 proteins met both fold change and p-value thresholds for differential expression in the three MtzR isolate lines. Correlation between protein and RNA fold changes [18] from same cell-pellet material in MtzR lines compared to MtzS were calculated at r^2= 0.154 for WB, r^2= 0.105 for WB and r^2= 0.187 for WB ($p < 0.01$) for genes identified in both datasets (Supplementary Figure 2). The largest number of differentially expressed proteins (DEPs) were identified in WB-MtzR with 264 DEPs, followed by 106-MtzR (171 DEPs) and then 713-MtzR (76 DEPs). Proportion of up-regulated and down-regulated proteins were approximately equal in each resistant line (Table 2). Of the non-redundant 443 DEPs, only seven (1.6%) were common between all three MtzR lines (Figure 1, Panel A). A further 55 DEPs (12.5%) were variously detected in two of the three lines. Overall, the majority of DEPs (86.4%) were unique to each line. This distinction between DEPs was not due to discrepancies in protein identifications between TMT 10plex experiments, as in each of the three 10plexes a total of 741 proteins were common identifications, constituting between 60.7-69.9% of proteins identified in each, and 47.2% of the non-redundant total (Figure 1, Panel A). Furthermore, the majority of DEPs in each MtzR line were identified in the other experiments (Supplementary Figure 3).

Of the seven DEPs common between MtzR lines there were six functionally annotated proteins, including two variant-specific surface protein (VSPs) (137620, 37093), one membrane-associated cysteine rich endopeptidase (14225), one EGF-like transmembrane protein, and two proteins with oxidoreductase activity, glutamate synthase (7195) and NR-1 (6175), the latter of which was significantly down-regulated in all MtzR lines, and is consistent with its role in Mtz activation and resistance [16, 19, 39].

Gene set enrichment analysis for differentially expressed proteins: The 264, 126 and 76 total DEPs in WB-MtzR, 106-MtzR, and 713-MtzR (relative to their isogenic susceptible parental control) respectively were DAVID for functional clustering analysis. A non-redundant total of six functional clusters (Supplementary Table S3) were identified as enriched among DEPs across the three MtzR isolates (Figure 1, Panel B), including 'AAA ATPase', two 'Electron Carrier Activity' clusters, 'EGF-like', 'Kinase' and 'Ribosome' clusters, with the 'EGF-like' cluster the most consistent in terms of gene families and numbers between MtzR lines. Overall, although DEP identifications and their directionality diverged between MtzR isolates, there was some convergence between MtzR lines at the level of protein function, with different members of the same gene or functional families detected among differentially regulated clusters of DEPs for each isolate. Functional annotation including GO and Interpro

annotations for DEPs can be found in Supplementary Table S2. Some of the clusters identified in the GSE were mirrored in results from STRING (Search Tool for the Retrieval of Interacting Genes) software, in particular protein-protein interactions surrounding ribosomal function, ABC transporters, antioxidant and glycolysis, and phosphorylation (Supplementary Figure 4).

The ‘Ribosome’ term assigned to ribosome structural constituents and related proteins was specifically enriched within WB-MtzR and reflected in its STRING interaction network (Supplementary Figure 4), with 29 DEPs compared to 5 and 3 in 106-MtzR and 713-MtzR respectively. A total of 24 structural constituents of ribosomes were down-regulated in WB-MtzR, which coincides with transcriptional data, which also observed transcripts of multiple ribosomal and ribosome-associated proteins as down-regulated in WB-MtzR [18]. A set of P-loop containing nucleotide hydrolases (IPR027417), particularly AAA+/AAA-type ATPase domains (IPR003593/IPR003959), were enriched among DEPs in three isolates, albeit with differences in directionality of expression, with 17, 11 and 7 DEPS in WB-MtzR, 106-MtzR and 713-MtzR, respectively. This gene set featured two main classes of proteins, including DNA/nucleic acid binding proteins, and transmembrane ABC transporters, which are frequently associated with the membrane translocation of toxic compounds, including drug compounds [40]. These ABC transporters were also detected as interaction partners through STRING interaction networks of DEPs in the three isolates (Supplementary Figure 4). In WB-MtzR three ABC transporters were up-regulated (113876, 28379), including one also up-regulated in 713-MtzR (16592), which is specifically involved in lipid transport (GO:0006869). However, in 106-MtzR the three differentially expressed ABC transporters were down-regulated (17132, 38104), including one also down-regulated in 713-MtzR (115052). Several proteins within this set were functionally related to transcriptional regulation, three of which were up-regulated in WB-MtzR (89112, 8228, 2098), and differentially expressed in 106-MtzR (112978, 8228). Further, the MAD-2 mitotic regulator was down-regulated in 713-MtzR, compared to universal up-regulation between isolates at the transcript level [18].

EGF-Like Proteins and VSPs: The ‘EGF-like’ gene set was enriched amongst DEPs all three MtzR isolates (Figure 2, Panel A). This set included an abundance of VSPs, with HCMPs as the second most pronounced group, particularly in 106-MtzR. The gene set also included EGF-like tenascin/notch-like proteins in WB-MtzR and 106 Mtz, which may be involved in signalling, and have been observed to increase during *in vitro* host-parasite interactions [41, 42]. Several other EGF-liked proteins were also present, including membrane-associated

cysteine-rich endopeptidases in 106-MtzR and 713-MtzR. Although EGF-like proteins, particularly cysteine-rich families, were consistently differentially expressed in all MtzR isolates, only a few DEPs were common to the three MtzR lines (Figure 2, Panel B) with WB-MtzR and 713-MtzR most separated.

Overall, a non-redundant total of 36 VSPs (defined according to Adam *et al* [43]) were identified in all three tenplexes, with fold change quantified in 23, 25 and 18 variants between MtzR and MtzS lines of WB, 106 and 713, respectively. The VSP gene family possessed largest proportion of DEPs amongst *Giardia* gene families, with 47.8%, 36.0% and 77.8% of VSPs differentially expressed in WB, 106 and 713-MtzR lines, respectively. Furthermore, the majority of differentially expressed VSPs were in the top 10 up- or down-regulated proteins in terms of fold change, specifically 9 of 11 VSPs in WB-MtzR were among the top DEGs, as were 7 of 9 in 106-MtzR and 9 of 13 in 713-MtzR. Although MtzR lines showed these similar trends in overall VSP differential expression, common specific VSP variants were limited between MtzR lines (Figure 2, Panel B), with only two common differentially expressed genes: VSP-123 (up-regulated in all MtzR lines), and VSP-25 (up-regulated in 106 and 713-MtzR, and down-regulated in WB-MtzR). Furthermore, MtzR lines also varied in directionality of differential expression (Figure 2, Panel C), with the majority of VSPs down-regulated in WB-MtzR, up-regulated in 713-MtzR, and dispersed between up- and down-regulated in 106-MtzR. Overall, although the expressed VSP complement of MtzR lines differed to their parent MtzS lines, cluster analysis also revealed divergences between MtzR lines as well (Figure 2, Panel C).

Oxidoreductase Enzymes, PFOR and pyruvate catabolism: The GO terms ‘electron carrier activity’ (GO:0009055) and ‘oxidation-reduction’ (GO:0055114) were enriched among DEPs in all three MtzR lines. In WB-MtzR, ‘iron-sulfur cluster’ (GO:0051536) and ‘iron ion binding’ (GO:0005506) annotation terms were also enriched. The overall expression of enzymes with oxidoreductase activity is shown in Figure 3, depicting enzymes specifically implicated in the *Giardia* antioxidant network (Figure 3, Panel A) [2, 44], and others involved in electron transport and cofactor abundance (Figure 3, Panel B). Overall, although oxidoreductases were significantly enriched within differentially expressed proteins in all three MtzR lines, MtzR lines had largely divergent expression profiles, indicative of alternative mechanisms and pathways for either reduced activation of Mtz or mitigation of oxidative damage. Among all three lines, NR-1 (22766) was universally significantly down-regulated.

WB-MtzR displayed the most prominent down-regulation trend of proteins in the antioxidant network (Figure 3, Panel A), with both PFOR proteins (114609, 17063) down-regulated, as well as PDI5 (8064), thioredoxin reducatase (9827), and a putative thioredoxin (3910). This indicates strong down-regulation Mtz-activating genes in WB-MtzR, and contrasts with up-regulation of the transcripts encoding these proteins in 713-MtzR [18]. In contrast, 713-MtzR down-regulated both a putative thioredoxin protein (3910) and a putative quinone reductase (17150). Although transcriptomics previously indicated down-regulation of quinone reductase as a universal mechanism of Mtz activation, the protein was not among DEPs in 106- and WB-MtzR. In 106-MtzR NR-1 (22766) was the only down-regulated oxidoreductase enzymes. By contrast, thioredoxin reductase and glutamate dehydrogenase (21942) were up-regulated in 106-MtzR, both of which have been previously implicated in Mtz resistance, but usually as being down-regulated [2, 10, 18]. No antioxidant-related proteins were up-regulated in 713-MtzR, while a Sir2 homologue (10708) implicated in redox-mediated epigenetic regulation of transcription [2, 18, 45] was up-regulated in WB-MtzR.

The extended oxidoreductase network was examined, specifically enzymes involved in cofactor abundance and electron transport, which showed trends towards up-regulation in 106-MtzR (Figure 3, Panel 2). The magnitude of differential expression in these enzymes was lower overall than DEPs in the antioxidant network, and again no common expression profiles emerged between MtzR lines. While glutamate synthase was differentially expressed in all three lines, it was up-regulated in 106- and 713-Mtz, and down-regulated in WB-MtzR. Recent models suggest this enzyme may be more structurally similar to trimethylamine (TMA) dehydrogenase of bacteria [44], which might indicate a role beyond amino acid metabolism (Figure 4). Many of the changes related to enzymes consuming NADPH or NADH are better contextualised relative electron transport via ferredoxin-containing enzymes (Figure 4). Thus 106-MtzR up-regulates multiple enzymes that may increase electron transport and the reducing potential available to antioxidant enzymes which activate Mtz. Lower transcript levels of glutamate dehydrogenase in all three lines [18], hypothesised to similarly conserve NADPH, was not observed in protein expression, with 106-MtzR in fact displaying higher abundance than its MtzS parents. In agreement with exisiting transcriptomics, threonine dehydratase was down-regulated in WB- and 713-MtzR, perhaps indicating a preference for pyruvate over alpha-ketobutyrate as a PFOR substrate, as well as its downstream metabolite acetyl-CoA.

Signalling and Kinases: Proteins involved in signalling, predominantly kinases, were particularly enriched in WB-MtzR (30 DEPs), which were also detected in protein-protein

interaction networks (Supplementary Figure 4). A total of 12 such DEPs were detected in 106-MtzR, of which five were shared with WB (6700, 113456, 17069, 17622, 3957), and one was shared between 106 and 713-MtzR (3677). The majority of differentially expressed kinases belonged to the uniquely expanded NEK kinase family in *Giardia*, with 16, 10 and 2 NEK kinases in WB, 106 and 713-MtzR lines, respectively. Multiple members of the NEK kinase family in *Giardia* are missing key catalytic amino acid residues, and are predicted not to have catalytic activity [46]. Seven of the 16 DEP NEKs in WB-MtzR are predicted to lack activity, as are 5/10 in 106-MtzR and 1/2 in 713-MtzR. Interestingly, 4 of the 5 most up-regulated NEK kinases in WB-MtzR were considered catalytically inert, as well as the most up-regulated NEK in 106-MtzR.

A further 14, 5 and 1 non-NEK kinases as well as 3, 5 and 1 phosphatases were differentially expressed in WB-MtzR, 106-MtzR and 713-MtzR, respectively. In 106-MtzR three regulatory subunits of protein phosphatase type 2A (PP2A) activity were up-regulated (9058, 4079, 17538), while inositol 5-phosphatase 4 (9077), and another serine/threonine phosphatase (2053) were down-regulated. In 713-MtzR the single serine/threonine phosphatase (2053) observed as down-regulated was up-regulated in the WB-MtzR line. Similarly, a further two phosphatases in WB-MtzR were up-regulated, including the PP2Ac phosphatase (5010), known to regulate encystation [47], and a PP2C phosphatase. Of the 14 differentially expressed, non-NEK kinases in WB-MtzR, 12 were up-regulated including a putative ethanolamine/choline kinase, while the 5 additional non-NEK kinases detected in 106-MtzR were down-regulated, including a phosphatidylinositol-4-phosphate 5-kinase (gPI4P5K) (13606) involved in lipid-based signalling [48].

Lipid Metabolism and Membrane Proteins: Multiple proteins involved lipid metabolism in *Giardia* [48] were differentially expressed in at least one MtzR isolate. Among proteins involved in phospholipid metabolism, PI transfer protein alpha isoform (PITPα) (4197), a phospholipid-transporting ATPase IIB (gPLTATPase IIB), and a putative gPLTATPase IIB (38104) were down-regulated in 106-MtzR, while PS synthase (gPSS) (17427) and another putative phospholipid ATPase transporter (16592) were up-regulated in 713-MtzR, with the later also up-regulated in WB-MtzR along with another putative acyltransferase (15987). PI transfer protein alpha isoform (PITPα) was also down-regulated in 106-MtzR. Furthermore, the highest up-regulated kinase in WB-MtzR was the putative ethanolamine/choline kinase, implicated in phospholipid metabolism [48].

A range of proteins involved in fatty acid lipid metabolism were also among DEPs. These included three fatty acid acyltransferases in WB-MtzR, including significant down-regulation of 1-acyl-sn-glycerol-3-phosphate acyl transferase (12109) and up-regulation of the glycylpeptide N-tetradecanoyltransferase homologue (5772), involved in protein myristoylation modifications. The majority of the long chain fatty acid CoA ligases (gLCFACL) were unchanged in expression in MtzR lines, though 17170 was up-regulated in both WB-MtzR and 713-MtzR. Among proteins involved in neutral lipid metabolism, only one putative phospholipase-B like protein (115159) was identified, which was up-regulated in 106-MtzR. Multiple proteins related to inositol signalling lipids were detected among DEPs, although none observed in 713-MtzR. In WB-MtzR, up-regulation of inositol 5-phosphatase 4 (gI5Pase) (9077) and inositol-3-phosphate synthase (17579) proteins was observed, while 106-MtzR in contrast down-regulated gI5Pase along with a putative gPI4P5K.

Additionally, a range of transmembrane and plasma membrane associated proteins were differential expressed in all MtzR lines, which have been summarised and sorted into functional categories in Table 3. Of the groups not previously addressed in section 3.4, several peptidases were observed as differentially expressed throughout MtzR lines. Some of these were EFG-like, cysteine-rich endopeptidases, with one (14225) universally down-regulated in all three lines. Additionally, several different dipeptidyl-peptidases were differentially expressed, which have been previously implicated in regulation and signal transduction relating to encystation [49]. These dipeptidyl-peptidases, while potentially linked to amino acid metabolism, are also secreted proteins [50, 51] which may allow them to compete with host amino acid metabolism. Several members from the annexin-like alpha-giardins which interact with phospholipids [52] were exclusively observed only within down-regulated proteins across MtzR lines, although no specific alpha-giardins were common between lines. Beta-giardin (4812), which is a microtubule associated protein in the adhesive disc [53], was also down-regulated in MtzR lines.

Post translational modifications in MtzR lines: Western blotting was used to assess protein post-translational modifications (PTMs) in trophozoite lysates of each MtzS and MtzR isogenic line (Figure 5). Overall, these PTM blots (phosphorylation, acetylation, methylation and ubiquitination) changed in both the appearance of new modified protein features in MtzR lines, as well as increases in intensity (abundance) of modified protein features in MtzR lines compared to MtzS parents. Total acetylated lysine (KAc) increased in all three MtzR lines as compared to the MtzS parents. This included the appearance of multiple new KAc-modified

protein features in MtzR lines, particularly in the 713-MtzR line. Four common protein bands (three: ~70-100 KDa; one ~25-50 KDa) with detectable KAc increased in intensity in all MtzR lines; albeit the smallest of the four bands was more highly expressed in 106- and 713-MtzR. Another three bands (~25-50KDa) with detected KAc increased in 713-MtzR. Mono-methylated lysine (K-MMe) also increased overall in all three MtzR lines, but most changes were unique to one, or observed in two of the three lines. Presumptive histone proteins H3 (~17kDa) and H4 (~11kDa) were detected by anti-KAc antibodies at the same molecular weight and pattern as previously detected by anti-KAc in enriched fractions [54], while H3 protein was also detected by anti-K-MMe antibodies. Both KAc and K-MMe modification detection for histone variants is consistent with previously detected histone modification states via Western blot in *Giardia* [55], and subsequent immunoblotting with Anti-H3 and Anti-H4 showed that the modified protein bands at ~17 and ~11KDa in KAc and KMMe blots aligned with antibodies for these histone variants (Supplementary Figure 5).

In order to interrogate the role of phosphorylation in MtzR, blots were performed to assess changes between isogenic lines in total tyrosine phosphorylation (pY) or the 14-3-3 substrate network. Although there were limited common fluctuations in pY modifications between lines, there were changes in the phosphorylated 14-3-3 substrate network. Changes in 14-3-3 sites included an increase and mass shift between MtzS and MtzR at protein band at ~90kDa, as well an increase in a protein band at ~25kDA. These were accompanied by increases in 5 protein bands common between 106- and 713-MtzR lines. Of the 314 known 14-3-3 interacting substrates [56], 139 proteins were detected in all three isolates, and 52 proteins were detected in a single or two of the isolates, constituting 60.8% of known substrates. However, only 57 of these substrates (18.2%) were detected among DEPs, indicating the majority of potential 14-3-3 substrates did not have significant changes in abundance. Lastly, Western blots targeting ubiquitin also detected two large protein bands at ~75 and ~55kDa increased in all three MtzR lines, as well increases in free ubiquitin (~10kDa) in resistant lines.

There were also several notable differences occurring in the 106-MtzS line distinguishing it from the other two isolates. These include a unique KAc protein band in 106-MtzS at ~37kDa, as well as two unique, high-intensity protein bands with K-MMe at ~30 and ~25 kDa. Lastly 106-MtzS displayed a divergent pY profile as compared to WB- and 713 MtzS, although the 106-MtzR line displayed a more congruent pY profile compared to WB- and 713MtzR.

Chemical inhibitors of post-translational protein modification networks: Four broad-spectrum chemical inhibitors of PTM networks were compared in MtzS and MtzR lines: deacetylase inhibitor Trichostatin A (TSA), broad spectrum methyltransferase inhibitor Chaetocin, kinase inhibitor Staurosporine and phosphatase inhibitor Calyculin A. Besides Chaetocin, all other inhibitors have been previously verified for activity in *Giardia* [55, 57]. The dose response curves, IC_{50} and resistance factors in MtzR lines for the four inhibitors are shown in Supplementary Figure 6. Staurosporine, Calyculin A and TSA were all highly effective against *Giardia*, with wild-type parent IC_{50} >1µM. Chaetocin, however, was only slightly effective against *Giardia*. Of the four inhibitors, all MtzR lines had significant increases in IC_{50} to TSA compared to their MtzS parents (Figure 6, Panel A), indicating Mtz resistance correlated with cross-resistance to TSA[55, 57] (Figure 6, Panel A), with TSA resistance factors ranging from +4.8 to +7.5 in MtzR lines compared to MtzS parents.

To explore the effect of TSA on protein acetylation in trophozoites, TSA exposures were performed and protein lysate immunoblotted. Trophozoites exposed to TSA remained both viable and adhered until 18 hours. Trophozoites were detached but viable at 24 hours, but were completely non-viable by 36 hours (data not shown). Exposure of trophozoites to TSA at 1µM, 2µM and 4µM for 8 hours demonstrated hyperacetylaton of histone variants (17 and 11kDa) in parent, wild-type lines, but not MtzR lines (Supplementary S7). When trophozoites were exposed to 2µM TSA for 18 hours (Figure 6, Panel B), wild-type MtzS parent lines showed trends towards increased acetylation, as expected with the inhibition of deacetylases, however MtzR isolates showed decreases in overall KAc, which was also observed across multiple concentrations of TSA at 8 hours (Supplementary Figure 7). In contrast MtzR lines did not show significant increases in histone variant hyperacetylation after 8 or 16 hours of TSA exposure (Figure 6, Panel B; Supplementary Figure 7).

MtzR stability after discontinuation of drug selection: Drug exposure was discontinued in 713 and 106-MtzR lines to observe effects on Mtz resistance (this was not undertaken in the WB-MtzR line due to impracticalities owing to its slow growth rate and lower adherence and confluence (Supplementary Figure 8)). MtzR lines were recovered and cultured for a week with Mtz drug selection, followed by 12 weeks of routine twice weekly passage (24 passages in total) without drug selection (Supplementary Figure 9). Both MtzR isolates had lower confluence levels during original drug selection (Week 1, P0; ~45%), which improved upon

discontinuation of drug selection. In order to achieve higher confluence for routine passage twice a week, higher seed volumes were required between passages for both isolates compared to MtzS lines, an effect that diminished over time as gains in growth were observed (Supplementary Figure 9). These improvements for *in vitro* growth occurred within 2 weeks in 106-MtzR, while fluctuations in growth rate requiring higher seeding volumes at passage were observed for 713-MtzR for up to 4 weeks (P8). As both WB-MtzR and 713-MtzR have reduced abundance of beta-giardin and SALP-1 (4410) proteins of the ventral disc [58], it is possible both lines have lower attachment (Supplementary Figure 8) due to changes in the structure of the ventral disc, and therefore lower confluence and slower division.

Mtz IC_{50} was calculated at each consecutive 4 week passage (P8, P16, P24: Figure 7**,** Panel A). After 4 weeks without Mtz selection, MtzR-P8 trophozoites in both lines had significantly increased Mtz susceptibility compared to MtzR lines (Table 1), with a lower IC_{50} in 106-MtzR-P8 of the two isolates. In MtzR-P16 trophozoites, 713-MtzR-P16 had further increases in Mtz susceptibility, while 106-MtzR-P16 increased in Mtz resistance as compared to 106-MtzR-P8, to levels equivalent to its drug-selected parent (i.e., 106-MtzR at week 0; Table 1). After 12 weeks (P24) without drug selection, IC50 in both lines plateaued, with no significant change in Mtz sensitivity compared to week 8 (P16) trophozoites. Based on this, 106-MtzR-P16 and 106-MtzR-P24 trophozoites retained both drug resistance and improved growth rates (Supplementary Figure 4) compared with their drug-selected parents [18]. At their lowest IC_{50} throughout the 12 weeks neither MtzR line returned to levels of Mtz susceptibility as recorded in parent MtzS lines, with lowest fold changes (resistance factor) in IC_{50} throughout the 12 weeks at +2.8 and +2.9 in 106-MtzR-P8 and 713-MtzR-P24, respectively.

Post-translational network stability upon discontinuation of drug selection: Divergent IC_{50} profiles showed 713-MtzR had consecutive gains in drug susceptibility at week 4 (P8) and week 8 (P16) upon cessation of drug selection, while the 106-MtzR line displayed its lowest Mtz susceptibility at week 4, then Mtz resistance equivalent to parental MtzR lines at week 8. Given large changes in PTM proteins were detected between MtzS and MtzR isogenic lines (Figure 5), total protein lysate from MtzR lines at weeks 4 and 8 was probed for fluctuations in PTM networks upon cessation of drug selection. (Figure 7, Panel B). KAc, K-MMe and pY networks showed significant fluctuations between MtzR-P0 and MtzR-P8 and MtzR-P16, as well isolate-specific variations between 713- and 106-MtzR. There were changes in the intensity of modifications of H3 and H4 variants for both lines. In 106-MtzR, both histone variants at 4 weeks without Mtz had increased KAc and K-MMe, while by 8 weeks KAc and

K-MMe modification levels still remained higher than MtzR selected lines on the H3 variant. In 713-MtzR, H4 KAc levels decreased, while H3 modifications were stable at 4 and 8 weeks. Together these suggest that the cessation of Mtz exposure caused significant changes to epigenetically-linked modifications.

Beyond histone proteins, KAc and K-MMe modification networks showed further fluctuations. The ~25kDa band observed in MtzR isolates was lost upon discontinuation of drug exposure, as were several lower intensity bands between ~70-100kDa. Two prominent bands observed at ~60 and ~50kDa showed significantly different intensity profiles between 713-MtzR-P0 and 106-MtzR-P0, increasing linearly in 713-MtzR-P8 and again in 713-MtzR-P16, while increasing in 106-MtzR-P8, then decreasing in 106-MtzR-P16 to levels lower than 106-MtzR-P0 lines. Changes in the K-MMe modification networks increasingly diverged between 713-MtzR and 106-MtzR. Overall, 713-MtzR displayed a trend to increased K-MMe in 713-MtzR-P8 and 713-MtzR-P16, including appearance of the methylated protein band at ~25kDa previously observed exclusively in 106-MtzS and 106-MtzR isolates (Figure 5). As observed for KAc, K-MMe was highest in trophozoites at 106-Mtz-P8, and then decreased comparatively at 106-MtzR-P16.

Numerous protein features were detected with anti-pY antibodies indicating many tyrosine kinase substrates in trophozoites (Figure 5), which underwent widespread changes upon discontinuation of Mtz exposure. Although a unique pY modification profile was observed for 106-MtzS (Figure 5), neither 106-MtzR-P8 nor 106-MtzR-P16 showed the same profile observed in 106-MtzS. However, by the end of 12 weeks the 106-MtzR line still possessed significant levels of Mtz resistance compared to its MtzS parent. There were multiple protein bands with divergent profiles in MtzR lines between isolates in the absence of drug selection, including two bands at ~27 and ~30kDa as well as multiple bands clustered between ~60-70kDa, although one protein band at ~125kDa increased in intensity in both isolates. Overall, discontinuation of Mtz selection widely altered phosphorylation signalling in both isolates.

Discussion:

Given the limited options for treating microaerophilic parasites, Mtz resistance is a major obstacle in the control of giardiasis and a widespread issue for metabolically related pathogens treated with nitroheterocyclics. Our study provides the first quantitative proteomic data for any MtzR pathogen to our knowledge. Further we performed the first exploration of protein PTM networks in MtzR parasites, and identified substantial changes in four major protein

modification networks (Figure 5). This indicates that PTM and differential protein expression may both contribute to Mtz resistance phenotypes. Further, we demonstrated trends of increased protein acetylation in MtzR lines, as well as cross-resistance to the histone deacetylase inhibitor TSA (Figure 6, Panel A), which also produced divergent KAc profiles in exposed MtzS and MtzR lines (Figure 6, Panel B; Supplementary Figure 7). Further, and perhaps most significantly, this study documented changes in Mtz susceptibility in two MtzR lines upon cessation of drug selection, and observed IC_{50} variation between isolates and widespread fluctuations in three PTM networks (Figure 7). However, a key aim of this three-way, isogenic analysis of MtzR lines was to reconcile observed inconsistencies of passive and active MtzR traits and isolate-dependent variation [2]. Our results show that although there was some equivalency at the functional level, differentially expressed proteins diverged considerably between isolates (Figure 1, Panel A), within protein families (Figure 2, Panel B and C), networks (Figure 3) and pathways (Figure 4). When interrogating post-translational networks, again multiple isolate-specific features differentiated each MtzR line (Figure 5), including some pre-existing, unique features in parental MtzS lines. Lastly, isolate variation was evident in Mtz sensitivity and PTM profiles upon exposure to TSA (Figure 6, Panel B) and cessation of Mtz selection (Figure 7). Overall, we believe this study confirms that MtzR is polygenic, plastic and likely post-translationally regulated, with particularly strong links between Mtz resistance and protein acetylation. Our data therefore supports earlier hypotheses suggesting links between MtzR and epigenetic regulation [18, 19], expands previously limited data concerning post-translation modifications in MtzR [20], and highlights for the first time that changes in post-translational modifications in MtzR are widespread.

Oxidoreductases, electron transport and active/passive MtzR mechanisms: The unique glycolytic and antioxidant system in aerophiles has been a primary focal point in the study of Mtz resistance, including for *Giardia*. Our results indicate that NR-1 (22677) was down-regulated in all MtzR lines, which concurs with previous results [16, 17, 19], including transcriptomics results for the same lines [18]. Previously, recombinant NR-1 was shown to reduce (i.e., activate) Mtz using NADH as a donor [59]. Ansell *et al* [18] subsequently hypothesised that NR-1 could utilise electrons from ferrodoxin to reduce Mtz, which could functionally link NR-1 to pyruvate catabolism and, by extension, to the PFOR-ferredoxin electron transport chain (Figure 4). Taken together, down-regulation of NR-1 is the strongest candidate for a universal passive resistance mechanism. Down-regulation of PFOR expression was observed only in WB-MtzR in this study, which agrees with transcriptomic data for WB-

MtzR and other resistant clones in this genotype [18, 19, 39], although not all clones [14]. It is also possible that reduced PFOR enzyme activity, previously observed in 106 and WB Mtz resistant lines [10, 12, 28], might lower the rate of Mtz activation to be functionally analogous as decreased PFOR protein expression. As such, combined investigations of enzyme activity and protein expression, may be required to test the interaction of different regulatory mechanisms in Mtz resistance.

Thioredoxin reductase was also inconsistent as a passive resistance mechanism, up- and down-regulated in 106-MtzR and WB-MtzR, respectively, but unchanged in 713-MtzR. This may be due to expression-independent changes to enzyme activity [12], or alternatively that down-regulation of thioredoxin reductase negatively impacts downstream thiol antioxidant systems, outweighing benefits of decreased Mtz activation. While down-regulation of thioredoxin reductase in WB-MtzR was accompanied by decreases in thioredoxin, in 106-MtzR the enzyme substrate of the increased reducing power postulated to result from up-regulated thioredoxin reductase is unclear. The wider profile of the oxidoreductase network also indicates a multiplicity of isolate-dependent expression patterns (Figure 3). For example, changes in the expression of oxidoreductases contributing to cofactor abundance and electron transport (Figure 3), including up-regulation of multiple enzymes involved in NAD(P)H consumption and production, were observed in 106-MtzR. Despite the absence of shared differentially expressed oxidoreductases, all MtzR lines exhibited functional enrichment of electron carrier activity (Figure 2, Panel B), indicating that Mtz variably affects the reducing power in the antioxidant network, the electron acceptors within glycolysis, as well as the critical relationship between them.

Some isotype-specific passive resistance mechanisms were also observed at the functional level. WB-MtzR down-regulated 29 structural ribosomal proteins, which was similarly observed in transcript data [18], and suggests significant reduction in ribosomal levels, protein production and *in vitro* generation times. While 106-MtzR down-regulated only 5 structural ribosomal constituents, two of the four genomically encoded FtsJ domain-containing rRNA methyltransferases (6055, 16993) were up-regulated. Null mutations in FtsJ are linked to altered ribosomal structures and impaired growth rates [60, 61], and indicate a potential link between post-translational methylation, growth rate and ribosome function in MtzR lines.

Cysteine-rich and membrane-associated proteins and lipids: Increasing evidence suggests that oxidative damage to cell membranes and its constituents can disrupt downstream, lipid-

based signalling [62, 63]. A range of proteins associated with lipid metabolism or the membrane (Table 3) were differentially expressed in each MtzR line, along with kinase and non-kinase proteins linked to lipid-based signaling. Oxidative damage to the membrane, particularly via lipid peroxidation or mutually competitive modifications, is well-documented in other systems [62], but not in *Giardia*, nor specifically in the context of oxidative stress or Mtz resistance. As such, metabolic analyses of the lipid and oxylipid composition of MtzR and MtzS lines would offer insights into the relationship between oxidative stress, Mtz and resistance.

Phospholipid-transporting ATPases (gPLTATPase) and gPSS have been suggested to form crucial links in the import and metabolism of phospholipids [48], and were up-regulated in WB-MtzR along with multiple genes involved in fatty acid intake, synthesis, modification and metabolism. This suggests lipid metabolism may be increased in some MtzR isolates, possibly to ensure membrane integrity for signaling pathways. Further to this, phosphatidylinositol pathways, which are associated with regulating cell growth in *Giardia* [48], were up-regulated in WB-MtzR, which has impaired growth rates compared to its MtzS parent [18]. In WB-MtzR, the highest up-regulated kinase was a putative choline/ethanolamine kinase responsible for initiating synthesis of phosphatidylcholine and phosphatidylethanolamine via phosphorylation. In *P. falciparum*, inhibition of choline/ethanolamine kinases leads to a significant decrease of phospholipids and arrest of parasite growth [64], and suggests links between lipid composition, oxidative damage and growth in WB-MtzR. 106-MtzR, in contrast, down-regulated phospholipid import and metabolism genes, as well as phosphatidylinositol pathways. Additionally, membrane associated dipeptidyl peptidases, which are known to regulate crucial proteolytic events during encystation [49] were differentially expressed across all lines, suggesting the importance of membrane signal-transduction events in *Giardia* MtzR phenotypes. Further, differentially expressed alpha-giardin proteins, which are known to interact with phospholipids in the membrane, were exclusively down-regulated in MtzR lines. Membrane lipid composition, such as un/saturation of phospholipid fatty acyl components, is known to change the sensitivity of the membrane to oxidative stress and damage [65], and increased fatty acid unsaturation is dynamically regulated during encystation in *Giardia* [66]. Although not detected in the proteomic dataset, at the transcript level [18] fatty acid elongase 1 (gFAELO) (92729) was up-regulated in WB-MtzR and 713-MtzR, along with three gLCFACL long chain fatty acid CoA ligases, providing further evidence of fatty acid composition changes in the membranes of MtzR isolates.

VSP variants in MtzR lines displayed similar trends in the proportion and magnitude of differentially expressed VSPs, although specific VSP variants and the direction of differential expression diverged, and MtzR lines were substantially different to MtzS parents, and each other (Figure 2, Panel B and C). This divergence may be a product of spontaneous VSP-variant switching [72] and differences in both time in culture and generation time for the six lines. However, large changes in VSP expression and turnover have been observed in other oxidative stress experiments [73], and it remains unclear if specific variant may have functional contributions to limit oxidative damage. Both VSP and HCMP gene families are rich in cysteine, which is the major low-molecular weight thiol in *Giardia* [74], and their multiple CXC/CXXC motifs potentially contribute to thiol and redox chemistries [75, 76]. Proteomic methods are available that allow selective modification to identify and discriminate reversible and irreversible oxidation states in cysteine [77, 78], which would provide evidence of the states of the multiple cysteines in VSPs, including those known to be lipid-modified [69]. While further evidence is required to determine whether specific *Giardia* VSPs contribute to alleviating oxidative stress [73] including Mtz-induced oxidative stress [18, 39], collective VSP protein expression as a measure of antigenic switching rates may sensitively indicate significant re-structuring of populations following xenobiotic or physiological stress. Large and statistically significant changes in VSP expression have been previously detected during proteomic analyses of encystation [79] and *in vitro* host-parasite models [41] in shorter timeframes than reported for spontaneous antigenic-switching [80]. This indicated that VSPs may be one of the most dynamic and sensitive protein families within *Giardia*.

Post-translation modifications networks in MtzR lines: To date the role of protein modifications in MtzR has been inferred through differential expression of their modifying enzymes [15, 18, 19] rather than modified substrates. Our results indicate that multiple proteins within MtzR lines are differentially modified by ubiquitination, phosphorylation, methylation and acetylation on multiple protein substrates. These modification marks varied in intensity and detectability within MtzR lines (Figure 5). This is significant if increases in these modifications are in site occupancy unaccompanied by increased protein abundance, as it would indicate regulation independent of expression levels. Probes for ubiquitin revealed three common protein bands that increased in MtzR lines, including free ubiquitin, and a fourth band that increased in 713- and 106-MtzR lines only (Figure 5). Seven ubiquitination and proteasome related proteins were differentially expressed across MtzR lines, six of which were in WB-MtzR. This correlates in WB-MtzR with significant differential expression of both

ribosomal proteins as well as ribosomal transcripts [18] indicating a potential for heightened demand of protein production to compensate for turnover of damaged proteins. Ubiquitination blots were highly congruent between isolates and lines, although only major protein bands were detected and lower abundance proteins may not have been detected. As mono-ubiquitinated proteins are associated with proteasome-independent processes, including gene transcription, while poly-ubiquitinated proteins are more likely to be associated with proteolysis and proteasome-degradation [81], analysis of the states of ubiquitination in MtzR lines would allow further extrapolation of the role of this modification in resistance.

Giardia has a uniquely reduced core kinome and a significantly expanded NEK kinome consisting of 80 and 278 kinases, respectively [46]. Our results indicated that both core and NEK kinases were differentially expressed and significantly enriched during functional clustering within MtzR lines. To survey changes to the phosphoproteome we examined tyrosine phosphorylation, and serine/threonine phosphorylation in the conserved 14-3-3 binding motif [(R/K)XX(s/t)XP] via Western blot. *Giardia* has a single 14-3-3 homologue with 314 documented protein substrates [56]. Immuno-detection of the 14-3-3 motif revealed multiple novel and increased intensity protein features in MtzR lines relative to the isogenic parents (Figure 5). However, our protein expression data showed that although 60.8% of known 14-3-3 substrates were detected among identified proteins, only 18.2% were among differentially expressed proteins, implying that the stoichiometric balance between modified and unmodified protein isomers is more variable than substrate abundance. Western blots of pY reinforce observations of Manning *et al* [46], that despite the absence of canonical tyrosine kinases or tyrosine-kinase like (TKL) kinases in the *Giardia* genome, pY is readily detectable and abundant across the proteome (Figure 5; Figure 7, Panel B). MtzR lines were quite congruent between isolates for pY, and (excluding the unique pY profile of 106-MtzS) shared many features with their MtzS parents. With pY most likely to catalysed by dual-specificity serine-threonine kinases [46], of which a wide range of which were differentially expressed in MtzR lines, including within the NEK kinase family, more work is required to define pY kinases in *Giardia*.

Dose response curves for kinase inhibitor Staurosporine and phosphatase inhibitor Calyculin A (Supplementary Figure 6) were similar and overlapping in MtzR and MtzS lines, with only minor shifts in IC_{50} between lines. Staurosporine and Calyculin A have significant efficacy against *Giardia* with IC_{50} for both compounds >50nm (Supplementary Figure 6), and have also been shown previously to block entry into and prevent exit from mitosis, respectively, although

their enzyme specificity and targets are not known [57], there is nonetheless a need for more targeted inhibitors to functionally probe specific phosphorylation pathways or enzymes between Mtz resistant and susceptible lines [57]. Therefore, more work is required to annotate core kinases in *Giardia* into subfamily classifications, define their homology and more broadly screen chemical inhibitors of phosphorylation.

Acetylation (KAc) may be an important histone modification in MtzR, however our data reveal KAc modifications occurs and changes on a wide range of protein substrates (Figure 5; Figure 7, Panel B). These changes appear linked to Mtz resistance, with all three MtzR lines showing increases in total KAc across a range of non-histone proteins compared to their MtzS parents, and cross-resistance to deacetylase inhibitor TSA (Figure 6, Panel A). This implies significant changes in lysine acetylase (KAT) or deacetylase (KDAC/HDAC) activity, which is further evidenced by differential KAc profiles and a lack of histone hyperacetylation in MtzR lines during TSA exposure (Figure 6, Panel B.; Supplementary Figure 7). There are five lysine acetyltransferases (KATs) and six lysine deacetylases (KDACs) encoded by the *Giardia* WB genome [54], which include five nuclear (four sirtuin (Sir2) KDACs and one NAD^+-independent HDAC) and one cytostolic (a Sir2 KDAC) deacetylase in trophozoites [55]. Although TSA is considered a Class I and II but not Class III (sirtuin) HDAC inhibitor, some *Giardia* sirtuins have very low homology to mammalian families [55] and more work is required to demonstrate TSA specifically inhibits the single NAD^+-independent HDAC in *Giardia*. Furthermore, given changes in KAc in response to TSA was not confined to histone variants (Figure 6, Panel B; Supplementary Figure 7), HDAC substrates also require identification. In Ansell *et al*, [18], four of the five KATs were transcriptionally down-regulated in 713-MtzR, along with numerous N-acetyltransferases. Among K/HDACs, the cytosolic Sir2 (10708) is significantly up-regulated at the transcript [18] and protein level in WB-MtzR and the NAD^+-independent, deacetylase (HDAC) is up-regulated at the protein level in 106-MtzR. [55]Sir2 KDACs are linked to increased longevity, antioxidant gene expression and cell cycle regulation in yeast during oxidative stress [82], whilst sirtuins in high eukaryotes are known to ameliorate oxidative stress by deacetylating enzymes and increasing their antioxidant activity [83, 84], and similar diverse KAc regulation may function in *Giardia* in MtzR lines.

KAc derives from acetyl-CoA, a substrate for protein acetylation, and is thus closely linked to central metabolism. In *Giardia* acetyl-CoA production is downstream of PFOR and the ferredoxin-based electron transport metabolism [85]. The PFOR metabolic node is perturbed

by multiple mechanisms in MtzR lines (Figure 4) which may influence acetyl-CoA availability, and in turn KAc modifications [18]. As such, MtzR phenotypes with either increased production, or decreased metabolism, of acetyl-CoA may have more substrate available for higher rates of KAc in MtzR lines [86, 87]. Metabolite-linked increases in KAc rates and substrates have been observed in *Plasmodium falciparum*, where addition of acetate increased the intracellular acetyl-CoA pool and downstream protein acetylation in rates of site occupancy, including for transcription factors and on histone variants [88]. As such, our observed increase of KAc in MtzR lines could trace back to both redox-regulated enzymatic (Sir2) as well as altered metabolic (acetyl-CoA) sources. Measuring acetyl-CoA levels in MtzS and MtzR lines would provide insight into metabolic disruptions downstream of PFOR and pyruvate metabolism, however further information on KAc substrates is still required, as these may also occur on, and influence activity of, key redox proteins in MtzR.

The *Giardia* genome encodes a highly reduced methylation network, with six histone lysine methyltransferases but no canonical arginine methyltransferases or demethylases [54, 89], and three methylation states (mono-, di-, and tri-methylation) observed on *Giardia* histone variants [55]. This significant reduction of the methylation network may explain the high IC_{50} of the broad-spectrum histone lysine methyltransferase Chaetocin (Supplementary Figure 6), which is significantly more effective in mammalian systems [90]. Substrates of lysine methylation beyond histones are unknown, although our results have demonstrated K-MMe is an extensive modification network in *Giardia* trophozoites (Figure 5; Figure 7, Panel B). Protein methylation modifications do not neutralise the substrate amino acid charge, as in acetylation and phosphorylation, or produce a significant mass shift as in ubiquitination, however, methylation still influences accessibility of protein-protein interactions and binding, particularly in gene regulation, where multiple methylation states are observed on histones, transcription factors and DNA-modifying enzymes [91]. All three MtzR lines showed increases overall in total K-MMe (Figure 5), with many features unique to each isolate or line. Three and two of the *Giardia* histone methyltransferases, which were below detection in our proteomic dataset are differentially transcribed in 713- and WB-MtzR relative to MtzS parental lines [18]. Conversely, in 106-MtzR, these enzymes are unchanged at the transcriptional level, however at the protein level displays up-regulation of a putative S-adenosylmethionine-dependent methyltransferase and two FtSJ rRNA methyltransferases. Although we can confirm lysine methylation as a correlate of MtzR across the proteome, substrate identities are now required.

Fitness costs, isolate variation and MtzR stability across lines*: In vitro* acquisition of MtzR variably affects trophozoite fitness among MtzR lines relative to their MtzS parents, implying varying clinical *in vivo* relevance. The *G. duodenalis* WB-MtzR line exhibits markedly decreased growth rate with the development of MtzR compared to its susceptible parent [18], and lower rates of trophozoite adherence and confluence (Supplementry Figure 8). Reduced growth rate is also reported for other MtzR lines [19], some of which also failed to encyst *in vitro*. While *in vitro* growth appears less affected in 106-MtzR and 713-MtzR lines, the ability of trophozoites to attach *in vitro* to inert surfaces (culture tubes) and *in vivo* to gastric epithelium (suckling mice intestine), is significantly impaired [14]. Of the three lines explored here, only 106-MtzR retains infectivity in suckling mice [14].

Our results add to evidence demonstrating MtzR exacts significant metabolic costs to *G. duodenalis*, but also highlights variability in the magnitude and reversibility of MtzR changes between and within genotypes. Indeed, the impaired growth rate and lower confluence of WB-MtzR relative to other MtzR lines [18] (Supplementary Figure 8) meant it was not possible to compare passage and time from cessation of drug selection equivalently as for 713- and 106-MtzR lines. The growth rate of 106-MtzR recovered from slower growth and lower confluency more quickly than 713-MtzR upon discontinuation of drug exposure (Supplementary Figure 9). Removing Mtz selection corresponds to restored transcription and enzyme activity of drug-activating enzymes and increased drug susceptibility [14, 21], in as little as four months. Transcriptomic analyses of WB, 713 and 106 isogenic lines indicated qualitative differences between 106-MtzR and WB- and 713-MtzR lines [18]. Correlations with previous studies revealed the 106-MtzR transcriptome to be most similar to the the 'wildtype' WB-MtzS after exposure to sub-lethal Mtz [15]. The 106-MtzR molecular phenotype is also potentially the most clinically relevant, in that it grows relatively quickly, remains cytoadherent and infective in suckling mice, whilst also showing a relatively stable MtzR phenotype after discontinuation of Mtz selection [14].

The source of isolate variation in Mtz tolerance both in the presence and absence of drug, particularly in context to the 106-MtzR line, is not clear. There are unresolved chromosomal aberrations in all three MtzR isogenic lines [92-94] as well nonsense mutations in multiple transcripts, including in NR-1 in 106-MtzR [18]. Western blots of protein post-translational modifications showed pre-existing differences in 106-MtzS for KAc, K-MMe and pY networks (Figure 5). Epigenetic regulation of transcriptional plasticity is implicated in MtzR and its stability, as the encystation-excystation process involves extensive epigenetic remodelling [54]

and restores Mtz susceptibility in formerly MtzR lines [19]. The lack of a histone H1 linker in *Giardia* has been proposed to shift reliance to histone modifications for chromatin remodelling and gene regulation [95], with acetylation and methylation already demonstrated as regulators of chromatin state for key processes of antigen switching and encystation [55, 96]. Although widespread changes were detected for KAc and K-MMe modifications between MtzR and MtzS lines, many of these occurred on non-histone substrates (Figure 5). In contrast, cessation of Mtz selection produced significant changes particularly on H3 and H4 modifications in MtzR lines (Figure 7, Panel B) albeit is an isolate dependent manner. Although histone acetylation generally correlates with transcriptional activation, methylation occurs in mono-, di and tri-methyl moieties that differentially influence gene expression and DNA methylation [91, 97]. Together, this indicates a suite of epigenetically-regulated fluctuations are likely to be occurring in transcription in 106-MtzR at 4 and 8 weeks, and that MtzR may be regulated through different mechanisms in 713-MtzR. Given emerging understanding of the links between histone modifications in oxidative stress responses [98], differences in Mtz- and oxygen-induced oxidative stress loads postulated in these lines may differentially influence epigenetic transcriptional phenotypes via epigenetic induction.

Conclusion: This study used three well-characterised isogenic MtzS and MtzR lines to investigate correlates of resistance at the proteomic and post-translational level in a genetically controlled design. Substantial genotypic variation was found in DEPs and post-translational marks. Together, this data regarding post-translational modifications as well as quantitative proteomics of protein abundance represents the most comprehensive post-transcriptional analysis of any pathogen in the context of nitroheterocyclic resistance to date, and suggests Mtz resistance, at least in *Giardia*, is significantly more complex than previously thought. Our data confirms that Mtz induces significant changes within proteins in antioxidant, electron transport and pyruvate catabolism networks in *Giardia*, of which NR-1 down-regulation coincides with multiple observations at the transcript level as a universal feature amongst a multiplicity of isolate-specific expression profiles (Figure 3). Our data also provides the first evidence to credit hypotheses linking acetylation to Mtz resistance, with increased acetylation in MtzR lines (Figure 5) as well as substantial cross-resistance to deacetylase inhibitor TSA (Figure 6). Further, given the relationship between KAc, PFOR expression, and production of acetyl-CoA, we hypothesise links between Mtz resistance, metabolism and protein modifications.

Lastly, our study has also provided novel insights through longitudinal surveillance of MtzR after discontinuation of drug selection, highlighting the loss of plastic traits and the potential of stable resistance traits. Our results also add to data that suggests 106-MtzR is both a clinically relevant [14] and transcriptionally unique [18] isotype, avoiding major fitness costs and retaining its MtzR phenotype *in vivo* and *in vitro* at parental MtzR IC_{50} levels at 12 weeks (Figure 7, panel A). Among a range of changes observed at the level of post-translational modifications, 106-MtzR showed significant fluctuations in KAc and K-MMe modifications of H3 and H4 variants (Figure 7, Panel B), the first evidence implicating epigenetic modifications in the stability of Mtz resistance. The interrogation of specific acetylation and methylation marks, as performed for differentiation and antigenic switching [55], would be the next step in investigating the role of chromatin state and transcriptional plasticity in Mtz resistance. Furthermore, given cross-resistance of Mtz resistance lines to TSA, further screening and characterisation of specific and targeted epigenetic, as well as broader PTM, chemical inhibitors are required to further probe post-translational regulation of Mtz resistance.

Methods

Isogenic Isolate Cell Culture: Trophozoites from each isolate were maintained in flat-sided 10 mL tubes (Nunclon delta) filled with complete TYI-S33 medium [85] containing 6 mM glucose and sub-cultured twice weekly. Mtz-resistant lines were cultured in the presence of Mtz (Sigma Aldrich; 100 mM stock dissolved in DMSO) at a final concentration of 30 µM, while parental lines were maintained in 1% DMSO. The Mtz-sensitive lines used in this study included WB1B (WB-MtzS), BRIS/83/HEPU/106 (106-MtzS) and BRIS/87/HEPU/713 (713-MtzS). Their respective Mtz-resistant progeny lines WB1B-M3 (WB-MtzR), BRIS/83/HEPU/106-2ID10 (106-MtzR) and BRIS/83/HEPU/713-M3 (713-MtzR). IC_{50} for Mtz for resistant and susceptible isolates was previously determined as detailed in Ansell *et al* [18]. Isolate nomenclature and references for axenisation and induction of Mtz resistance induction are provided in Table 1.

Protein Extraction, Digestion and TMT labelling for Proteomics: Trophozoites cultures for protein extraction were generated as previously described [18]. Briefly, trophozoites were seeded at a number normalized to growth rate in order to achieve equivalent final cell numbers in t25 flasks (Falcon), followed by decanting of media and unattached and non-viable trophozoites. Adherent trophozoites were then harvested by chilling trophozoites in fresh,

complete TYI-S33 media on wet ice before collection by centrifugation. Total protein, RNA [18] and DNA was extracted using the TriPure reagent (Roche) from the same trophozoite pellet material according to the manufacturer's instructions.

Protein pellets were solubilised in 2% SDS in 50 mM Tris (pH 8.8) (Sigma Aldrich) before reduction in 5 mM dithiothreitol followed by alkylation in 10 mM iodoacetamide in the dark (with alkylation quenched with 5mM dithiothreitol). To removed interfering reagents proteins were precipitated via Methanol/Chloroform approach [99] and transferred to 8 M Urea in 50 mM Tris (pH 8.8) and protein concentration quantitated by BCA assay (Pierce). A two-stage digestion was performed with firstly Lys-C (Wako) overnight at 30°C (1 µg enzyme to 100 µg protein), followed with Trypsin (Promega) digestion 37°C (1 µg enzyme for 100 µg protein) for 6 hours. Samples were acidified with trifluoroacetic acid to 1% concentration acid and then desalted using solid phase extraction (SPE) with in-house tips packed with styrene divinyl benzene (3M Empore). Peptide extracts were dried by vacuum centrifuge, reconstituted in 200 mM HEPES (pH 8), followed by quantification via Micro BCA (Pierce).

For TMT labelling, a total of 35 mg of peptides per sample were used for each TMT label reaction. Samples were labelled across three TMT 10plex reactions (Thermo, San Jose, CA) using 0.14mg of each reagent, with each of the three TMT 10-plex experiments containing MtzS and MtzR replicates of each of the three isolates (WB, 106 and 713). Samples were incubated with labels for 1 hour at room temperature, and then quenched with 5% hydroxylamine (Sigma Aldrich). Each of the 10 labelled samples for each of the three TMT 10-plex experiments were combined, dried by vacuum centrifuge, reconstituted in 1% formic acid, and desalted on a 200 mg C18 SepPak (Waters, Massachusetts) prior to SCX fractionation as described previously [41]. A total of 10 pooled SCX fractions were desalted using SPE as before, dried down using a vacuum centrifuge and reconstituted in 1% formic acid for nanoflow liquid chromatography tandem mass spectrometry (NanoLC-MS/MS).

Nanoflow LC-MS/MS of TMT-labelled peptides: MS analysis was performed on a Q Exactive Orbitrap (Thermo Scientific) coupled to an EASY-nLC1000 (Thermo Scientific) as previously described [41]. Reversed-phase chromatographic separation was performed on a 75 µm id. × 100 mm, C18 HALO column, 2.7 µm bead size, 160 Å pore size. Samples were run on a linear gradient of 1-30% solvent B (99.9% ACN/0.1% FA) over 170 minutes, with the Q Exactive operating in the data-dependent mode to automatically switch between Orbitrap MS and ion trap MS/MS acquisition. Survey full scan MS spectra (from m/z 350 to 1850) were

acquired with a resolution of 70,000 at m/z 400 and an AGC (Automatic Gain Control) target value of 1×10^6 ions. The top ten most abundant ions were selected for higher energy collisional dissociation (HCD) fragmentation, with HCD normalised collision energy set to 35% and fragmentation ions detected in the Orbitrap at a resolution of 70 000. Dynamic exclusion of target ions selected for MS/MS was set to 90 seconds and the lock mass option was also enabled using the polydimethylcyclosiloxane ion (m/z 445.12003) as an internal calibrant.

Database Searching: Raw data files produced in Xcalibur (Thermo Scientific) were processed in Proteome Discoverer V1.3 (Thermo Scientific) and searched using Mascot against the WB C6 (ATCC 50803) V5.0 genome release obtained from GiardiaDB.org [50]. Parameters and modifications were as follows: MS tolerance was set to ±10 ppm, MS/MS tolerance to 0.1 Da, one missed cleavage was allowed; static modifications were set for carbamidomethylation of cysteines, while variable modifications were set to TMT 10plex modification of peptide N-termini and lysine residues, methionine oxidation, and deamidation of asparagine and glutamine. Search results only included peptides with a score > 15 and below the Mascot significance threshold filter of p = 0.05. FDR was set for 1% and protein grouping for homologous peptide identification was enabled such that protein identifications based of peptides with amino acid sequences equal to, or contained within the sequence of more than one protein, the two proteins were grouped together in a single protein group. The mass spectrometry raw data files, database search results and TMT ratios have all been deposited to the ProteomeXchange Consortium [29] via the PRIDE partner repository with the dataset identifier PXD007183**.**

Analysis of differentially expressed proteins: Relative quantitation of protein abundance in MtzR compared to MtzS isogenic lines were derived from the ratio of TMT label detected in each MtzR to MtzS replicates. As such, a total of nine ratios for each MtzR vs MtzS comparison, and the geometric mean was calculated to establish the fold change for each protein identified. Further to ratio-derived fold changes, protein abundance between MtzR and MtzS lines were evaluated statistically via a one-sample t-test using the tenth channel (pooled control) to normalise MtzR and MtzS replicate labels. Differential expression required proteins to meet both ratio fold change (< 1.3 or > 0.77) and a significant p-value (> 0.05) [36, 37]. Further statistical evaluation of the dataset was performed, with an unsupervised multivariate principal component analysis (PCA) performed on the entire dataset using the log-transformed ratios of samples over the pooled control (tenth channel), and an analysis of the p-value distribution using paired t-tests between triplicates of HSF/Control and CI/Control ratios. The

Pearson correlation between log-transformed fold change in transcript [18], and protein abundances, was calculated in R and visualized using the ggplot2 library. For brevity, the gene accession prefix, GL50803, is omitted during further discussions of individual genes.

Gene set enrichment analysis (GSEA): Functional annotation of proteins was performed using Uniprot to assign gene ontology (GO) function, subcellular localisation, Interpro protein domains and structure annotations where available. GSEA was performed on differentially expressed proteins using the DAVID bioinformatics resource [100]. GiardiaDB.org ORF identifiers from combined up- and down-regulated proteins in each MtzR isolate were converted to gene identifiers using the NCBI Batch Entrez tool (http://www.ncbi.nlm.nih.gov/sites/batchentrez). Converted gene identifier lists were submitted by isolate to DAVID for GSEA, with GO annotations and Interpro annotations submitted for testing. Gene sets with an EASE score ≤ 0.2 in at least one MtzR line were retained.

Protein-Protein interaction networks: Network analysis was performed by submitting DEPs ascensions to the STRING (Search Tool for the Retrieval of Interacting Genes) software (v10.5) (http://string.db.org) [101]. Interaction networks were visualised for proteins with medium confidence (0.4) with network edges based on confidence, with continuous lines for direct interactions and indirect interactions with interrupted lines. Clustering was based on a MCL inflation default parameter of 3.

MtzR Revertant Cell Culture: Trophozoites from the lines 106-MtzR and 713-MtzR were cultured in TYI-S33 in the presence of Mtz as above and designated passage 0 (P0). Subsequently, Mtz drug selection was discontinued, and isolates were sub-cultured twice weekly without Mtz. MtzR revertant cultures were preserved every 4 weeks. IC_{50} values were determined for cells at P8, P16 and P24 relative to susceptible parent isolates as detailed in Section 2.1 in Ansell *et al* [18].

Western Blotting of Post-translational protein modifications: Adhered trophozoites grown to confluence from all MtzR and MtzS isogenic isolates, as well as 106-MtzR and 713-MtzR P0, P8 and P16 revertant cultures, had protein extracted in 2.5% SDS in 100 mM Tris containing 5 mM Trichostatin A (BioAustralis) and HALT® protease and phosphatase inhibitor (Life Technologies). Protein concentration was determined via BCA assay (Pierce), and then samples were reduced with 15 mM dithiothreitol at 90˚C. A total of 15 ug of proteins were resolved on 4-12% Bis-Tris gradient gels (Invitrogen) in 1 ×3-(N-

morpholino)propanesulfonic acid running buffer (Invitrogen) and were transferred to nitrocellulose membranes (Sigma). All antibodies were obtained from Cell Signalling Technologies, and included antibodies directed to acetylated lysine (KAc), mono-methylated lysine (K-MMe), phosphorylated tyrosine (pY), the 14-3-3 binding motif (including phosphorylated serine) (14-3-3) and Ubiquitin (Ubi). Anti-H3 and Anti-H4 antibodies were obtained from Abcam. Consistent protein loading was verified after transfer using Ponceau S staining (Sigma). Protein-antibody interaction was detected with a HRP-conjugated IgG secondary antibody using enhanced chemiluminescent reagent (LumiGLO®, Cell Signalling Technologies) on a BioRad ChemiDoc MP imaging system with exposure times optimised for each antibody, and images collected up to 200 seconds and selected based on resolution and absence of oversaturation

Chemical Inhibitors of post-translation protein modification networks: Staurosporine, Chaetocin, TSA were obtained from BioAustralis and Calyculin A from Sigma. For each of the chemical inhibitors, IC_{50} was calculated as previously determined as detailed in section 2.1 and as in Ansell *et al* [18] in both MtzS and MtzR lines. All stocks were solubilised in DMSO

TSA exposure courses were optimised at 1, 2 and 4µM TSA for 8 hours after 48 hours growth prior to exposure, and then exposed to 2µM for 18 hours after 72 hours growth prior to exposure. For both exposure courses, trophozoites were grown without the addition of Mtz in MtzR lines, and only adherent trophozoites were used in exposure timecourses. Prior to TSA exposure, non-adhered trophozpoites were discarded along with the old media, with fresh media added containing either TSA in DMSO or DMSO only (control) added. Adherence was monitored throughout exposure timecourses, with detachment indicating decreasing viability. Trophozoites from exposure experiments had protein extracted and analysed using Anti-KAc as detailed previously, with 10µg of protein run on the gels.

Availability of Supporting Data: Proteomic datasets including raw files, mascot search files and TMT protein ratios can be accessed for free at the European Bioinformatics PRoteomics IDEntifications (PRIDE) database via ProteomeXchange with identifier PXD007183. Supporting data are also available via the *GigaScience* repository GigaDB [108].

Abbreviations: DEPs, Differentially Expressed Proteins; gFAELO, Fatty Acid Elongase 1; GSEA, Gene Set Enrichment Analysis; GO, Gene Ontology; HCMP, High Cysteine Membrane Protein; HDAC, Histone Deacetylase; HAT, Histone Acetyltransferase; IECs, Intestinal Epithelial Cells; KAc, Acetylated Lysine; KDa, Kilodalton; KDAC, Lysine Deacetylase; KAT, Lysine Acetyltransferase; K-MMe, Mono-methylated lysine; gLCFACL, Long Chain Fatty Acid CoA ligases; MtZ, Metronidazole; NanoLC-MS/MS, nanoflow liquid chromatography tandem mass spectrometry; NR, Nitroreductase; PCA, Principal Component Analysis; PFOR, Pyruvate Ferrodoxin Oxidoreductase; gPI4P5K, Phosphatidylinositol-4-phosphate 5-kinase; gPLTATPase IIB, Phospholipid-transporting ATPase IIB; PITPα, PI transfer protein alpha isoform; PP2A, Protein Phosphatase type 2A; gPSS, Phosphatidylserine synthase; pY, Phosphorylated Tyrosine; SNPs, Single Nucleotide Polymorphisms; SPE, Solid Phase Extraction; TSA, Trichostatin A; TMT, Tandem Mass Tags; Ubi, Ubiquitin; VSP, Variant-specific Surface Protein

Acknowledgements: This work, including the efforts of AJ and MM, was funded by Australian Research Council (ARC) (LP120200122). SE, BA, LB and AJ are supported by the Victorian State Government Operational Infrastructure Support and Australian Government National Health and Medical Research Council Independent Research Institute Infrastructure Support Scheme. AJ is also supported by a NHMRC Career Development Fellowship (APP1126395). SE and this research is also supported by a Jack Brockhoff Foundation Early Career Grant (ID 4184). Proteomic analysis was performed at the Australia Proteomics Analysis Facility (APAF) at Macquarie University. The funders had no role in study design, data collection and interpretation, or the decision to submit the work for publication.

Conflict of Interest Statement: The authors declare that the research was conducted in the absence of any commercial or financial relationships that could be construed as a potential conflict of interest.

Author Contributions: BA, AJ and LB designed the experiment. BA and LB generated the samples. SE and MM processed the samples for proteomics, and performed the mass spectrometry. SE analysed the data. SE, BA, AJ, MM, PH, MJM, SS wrote the manuscript.

References

1. Sneader WE. Drug Discovery (The History). Wiley Online Library; 2005.

2. Ansell BR, McConville MJ, Ma'ayeh SY, Dagley MJ, Gasser RB, Svard SG, et al. Drug resistance in *Giardia duodenalis*. Biotechnol Adv. 2015;33 6 Pt 1:888-901. doi:10.1016/j.biotechadv.2015.04.009.

3. Townson SM, Boreham PF, Upcroft P and Upcroft JA. Resistance to the nitroheterocyclic drugs. Acta Trop. 1994;56 2-3:173-94.

4. Carter ER, Nabarro LE, Hedley L and Chiodini PL. Nitroimidazole-refractory giardiasis; a growing problem requiring rational solutions. Clin Microbiol Infect. 2017; doi:10.1016/j.cmi.2017.05.028.

5. Lane S and Lloyd D. Current trends in research into the waterborne parasite *Giardia*. Crit Rev Microbiol. 2002;28 2:123-47. doi:10.1080/1040-840291046713.

6. Ankarklev J, Jerlstrom-Hultqvist J, Ringqvist E, Troell K and Svard SG. Behind the smile: cell biology and disease mechanisms of *Giardia* species. Nat Rev Microbiol. 2010;8 6:413-22. doi:10.1038/nrmicro2317.

7. Solaymani-Mohammadi S, Genkinger JM, Loffredo CA and Singer SM. A meta-analysis of the effectiveness of albendazole compared with metronidazole as treatments for infections with *Giardia duodenalis*. PLoS Negl Trop Dis. 2010;4 5:e682. doi:10.1371/journal.pntd.0000682.

8. Gardner TB and Hill DR. Treatment of giardiasis. Clin Microbiol Rev. 2001;14 1:114-28. doi:10.1128/CMR.14.1.114-128.2001.

9. Nabarro LE, Lever RA, Armstrong M and Chiodini PL. Increased incidence of nitroimidazole-refractory giardiasis at the Hospital for Tropical Diseases, London: 2008-2013. Clin Microbiol Infect. 2015;21 8:791-6. doi:10.1016/j.cmi.2015.04.019.

10. Leitsch D, Burgess AG, Dunn LA, Krauer KG, Tan K, Duchene M, et al. Pyruvate:ferredoxin oxidoreductase and thioredoxin reductase are involved in 5-nitroimidazole activation while flavin metabolism is linked to 5-nitroimidazole

resistance in *Giardia lamblia*. J Antimicrob Chemother. 2011;66 8:1756-65. doi:10.1093/jac/dkr192.

11. Liu SM, Brown DM, O'Donoghue P, Upcroft P and Upcroft JA. Ferredoxin involvement in metronidazole resistance of *Giardia duodenalis*. Mol Biochem Parasitol. 2000;108 1:137-40.

12. Townson SM, Upcroft JA and Upcroft P. Characterisation and purification of pyruvate:ferredoxin oxidoreductase from *Giardia duodenalis*. Mol Biochem Parasitol. 1996;79 2:183-93.

13. Upcroft JA, Upcroft P and Boreham PF. Drug resistance in *Giardia intestinalis*. Int J Parasitol. 1990;20 4:489-96.

14. Tejman-Yarden N, Millman M, Lauwaet T, Davids BJ, Gillin FD, Dunn L, et al. Impaired parasite attachment as fitness cost of metronidazole resistance in *Giardia lamblia*. Antimicrob Agents Chemother. 2011;55 10:4643-51. doi:10.1128/AAC.00384-11.

15. Ansell BR, McConville MJ, Baker L, Korhonen PK, Emery SJ, Svard SG, et al. Divergent Transcriptional Responses to Physiological and Xenobiotic Stress in *Giardia duodenalis*. Antimicrob Agents Chemother. 2016;60 10:6034-45. doi:10.1128/AAC.00977-16.

16. Muller J, Schildknecht P and Muller N. Metabolism of nitro drugs metronidazole and nitazoxanide in *Giardia lamblia:* characterization of a novel nitroreductase (GlNR2). J Antimicrob Chemother. 2013;68 8:1781-9. doi:10.1093/jac/dkt106.

17. Muller J, Wastling J, Sanderson S, Muller N and Hemphill A. A novel *Giardia lamblia* nitroreductase, GlNR1, interacts with nitazoxanide and other thiazolides. Antimicrob Agents Chemother. 2007;51 6:1979-86. doi:10.1128/AAC.01548-06.

18. Ansell BR, Baker L, Emery SJ, McConville MJ, Svard SG, Gasser RB, et al. Transcriptomics Indicates Active and Passive Metronidazole Resistance Mechanisms in Three Seminal *Giardia* Lines. Front Microbiol. 2017;8:398. doi:10.3389/fmicb.2017.00398.

19. Muller J, Ley S, Felger I, Hemphill A and Muller N. Identification of differentially expressed genes in a *Giardia lamblia* WB C6 clone resistant to nitazoxanide and metronidazole. J Antimicrob Chemother. 2008;62 1:72-82. doi:10.1093/jac/dkn142.

20. Uzlikova M and Nohynkova E. The effect of metronidazole on the cell cycle and DNA in metronidazole-susceptible and -resistant *Giardia* cell lines. Mol Biochem Parasitol. 2014;198 2:75-81. doi:10.1016/j.molbiopara.2015.01.005.

21. Smith NC, Bryant C and Boreham PF. Possible roles for pyruvate:ferredoxin oxidoreductase and thiol-dependent peroxidase and reductase activities in resistance to nitroheterocyclic drugs in *Giardia intestinalis*. Int J Parasitol. 1988;18 7:991-7.

22. Teodorovic S, Walls CD and Elmendorf HG. Bidirectional transcription is an inherent feature of *Giardia lamblia* promoters and contributes to an abundance of sterile antisense transcripts throughout the genome. Nucleic Acids Res. 2007;35 8:2544-53. doi:10.1093/nar/gkm105.

23. Best AA, Morrison HG, McArthur AG, Sogin ML and Olsen GJ. Evolution of eukaryotic transcription: insights from the genome of *Giardia lamblia*. Genome Res. 2004;14 8:1537-47. doi:10.1101/gr.2256604.

24. Knodler LA, Svard SG, Silberman JD, Davids BJ and Gillin FD. Developmental gene regulation in *Giardia lamblia*: first evidence for an encystation-specific promoter and differential 5' mRNA processing. Mol Microbiol. 1999;34 2:327-40.

25. Boreham PF, Upcroft J, Upcroft P and Andrews R. Zoonotic *Giardia* - the debate goes on. Parasitol Today. 1988;4 11:322.

26. Capon AG, Upcroft JA, Boreham PF, Cottis LE and Bundesen PG. Similarities of *Giardia* antigens derived from human and animal sources. Int J Parasitol. 1989;19 1:91-8.

27. Upcroft JA, Boreham PF, Campbell RW, Shepherd RW and Upcroft P. Biological and genetic analysis of a longitudinal collection of *Giardia* samples derived from humans. Acta Trop. 1995;60 1:35-46.

28. Dunn LA, Burgess AG, Krauer KG, Eckmann L, Vanelle P, Crozet MD, et al. A new-generation 5-nitroimidazole can induce highly metronidazole-resistant *Giardia lamblia* in vitro. Int J Antimicrob Agents. 2010;36 1:37-42. doi:10.1016/j.ijantimicag.2010.03.004.

29. Vizcaino JA, Cote RG, Csordas A, Dianes JA, Fabregat A, Foster JM, et al. The PRoteomics IDEntifications (PRIDE) database and associated tools: status in 2013. Nucleic Acids Res. 2013;41 Database issue:D1063-9. doi:10.1093/nar/gks1262.

30. Boreham PF, Phillips RE and Shepherd RW. Altered uptake of metronidazole in vitro by stocks of *Giardia intestinalis* with different drug sensitivities. Trans R Soc Trop Med Hyg. 1988;82 1:104-6.

31. Townson SM, Laqua H, Upcroft P, Boreham PF and Upcroft JA. Induction of metronidazole and furazolidone resistance in *Giardia*. Trans R Soc Trop Med Hyg. 1992;86 5:521-2.

32. Upcroft JA, Campbell RW, Benakli K, Upcroft P and Vanelle P. Efficacy of new 5-nitroimidazoles against metronidazole-susceptible and -resistant *Giardia*, *Trichomonas*, and *Entamoeba* spp. Antimicrob Agents Chemother. 1999;43 1:73-6.

33. Upcroft JA, Dunn LA, Wright JM, Benakli K, Upcroft P and Vanelle P. 5-Nitroimidazole drugs effective against metronidazole-resistant *Trichomonas vaginalis* and *Giardia duodenalis*. Antimicrob Agents Chemother. 2006;50 1:344-7. doi:10.1128/AAC.50.1.344-347.2006.

34. Nageshan RK, Roy N, Hehl AB and Tatu U. Post-transcriptional repair of a split heat shock protein 90 gene by mRNA trans-splicing. J Biol Chem. 2011;286 9:7116-22. doi:10.1074/jbc.C110.208389.

35. Emery SJ, Lacey E and Haynes PA. Quantitative proteomic analysis of *Giardia duodenalis* assemblage A: A baseline for host, assemblage, and isolate variation. Proteomics. 2015;15 13:2281-5. doi:10.1002/pmic.201400434.

36. Mahoney DW, Therneau TM, Heppelmann CJ, Higgins L, Benson LM, Zenka RM, et al. Relative quantification: characterization of bias, variability and fold changes in

mass spectrometry data from iTRAQ-labeled peptides. J Proteome Res. 2011;10 9:4325-33. doi:10.1021/pr2001308.

37. Pascovici D, Handler DC, Wu JX and Haynes PA. Multiple testing corrections in quantitative proteomics: A useful but blunt tool. Proteomics. 2016;16 18:2448-53. doi:10.1002/pmic.201600044.

38. Pounds SB. Estimation and control of multiple testing error rates for microarray studies. Brief Bioinform. 2006;7 1:25-36.

39. Muller J, Sterk M, Hemphill A and Muller N. Characterization of *Giardia lamblia* WB C6 clones resistant to nitazoxanide and to metronidazole. J Antimicrob Chemother. 2007;60 2:280-7. doi:10.1093/jac/dkm205.

40. Glavinas H, Krajcsi P, Cserepes J and Sarkadi B. The role of ABC transporters in drug resistance, metabolism and toxicity. Curr Drug Deliv. 2004;1 1:27-42.

41. Emery SJ, Mirzaei M, Vuong D, Pascovici D, Chick JM, Lacey E, et al. Induction of virulence factors in *Giardia duodenalis* independent of host attachment. Sci Rep. 2016;6:20765. doi:10.1038/srep20765.

42. Ferella M, Davids BJ, Cipriano MJ, Birkeland SR, Palm D, Gillin FD, et al. Gene expression changes during *Giardia*-host cell interactions in serum-free medium. Mol Biochem Parasitol. 2014;197 1-2:21-3. doi:10.1016/j.molbiopara.2014.09.007.

43. Adam RD, Nigam A, Seshadri V, Martens CA, Farneth GA, Morrison HG, et al. The *Giardia lamblia* vsp gene repertoire: characteristics, genomic organization, and evolution. BMC genomics. 2010;11:424. doi:10.1186/1471-2164-11-424.

44. Ansell BR, McConville MJ, Baker L, Korhonen PK, Young ND, Hall RS, et al. Time-Dependent Transcriptional Changes in Axenic *Giardia duodenalis* Trophozoites. PLoS Negl Trop Dis. 2015;9 12:e0004261. doi:10.1371/journal.pntd.0004261.

45. Religa AA and Waters AP. Sirtuins of parasitic protozoa: in search of function(s). Mol Biochem Parasitol. 2012;185 2:71-88. doi:10.1016/j.molbiopara.2012.08.003.

46. Manning G, Reiner DS, Lauwaet T, Dacre M, Smith A, Zhai Y, et al. The minimal kinome of *Giardia lamblia* illuminates early kinase evolution and unique parasite biology. Genome Biol. 2011;12 7:R66. doi:10.1186/gb-2011-12-7-r66.

47. Lauwaet T, Davids BJ, Torres-Escobar A, Birkeland SR, Cipriano MJ, Preheim SP, et al. Protein phosphatase 2A plays a crucial role in *Giardia lamblia* differentiation. Mol Biochem Parasitol. 2007;152 1:80-9. doi:10.1016/j.molbiopara.2006.12.001.

48. Yichoy M, Duarte TT, De Chatterjee A, Mendez TL, Aguilera KY, Roy D, et al. Lipid metabolism in *Giardia*: a post-genomic perspective. Parasitology. 2011;138 3:267-78. doi:10.1017/S0031182010001277.

49. Touz MC, Nores MJ, Slavin I, Piacenza L, Acosta D, Carmona C, et al. Membrane-associated dipeptidyl peptidase IV is involved in encystation-specific gene expression during *Giardia* differentiation. Biochem J. 2002;364 Pt 3:703-10. doi:10.1042/BJ20020025.

50. Aurrecoechea C, Brestelli J, Brunk BP, Carlton JM, Dommer J, Fischer S, et al. GiardiaDB and TrichDB: integrated genomic resources for the eukaryotic protist pathogens *Giardia lamblia* and *Trichomonas vaginalis*. Nucleic Acids Res. 2009;37 Database issue:D526-30. doi:10.1093/nar/gkn631.

51. Ma'ayeh SY, Liu J, Peirasmaki D, Hornaeus K, Bergstrom Lind S, Grabherr M, et al. Characterization of the *Giardia intestinalis* secretome during interaction with human intestinal epithelial cells: The impact on host cells. PLoS Negl Trop Dis. 2017;11 12:e0006120. doi:10.1371/journal.pntd.0006120.

52. Weiland ME, McArthur AG, Morrison HG, Sogin ML and Svard SG. Annexin-like alpha giardins: a new cytoskeletal gene family in *Giardia lamblia*. Int J Parasitol. 2005;35 6:617-26. doi:10.1016/j.ijpara.2004.12.009.

53. Macarisin D, O'Brien C, Fayer R, Bauchan G and Jenkins M. Immunolocalization of beta- and delta-giardin within the ventral disk in trophozoites of *Giardia duodenalis* using multiplex laser scanning confocal microscopy. Parasitol Res. 2012;111 1:241-8. doi:10.1007/s00436-012-2825-x.

54. Sonda S, Morf L, Bottova I, Baetschmann H, Rehrauer H, Caflisch A, et al. Epigenetic mechanisms regulate stage differentiation in the minimized protozoan *Giardia lamblia*. Mol Microbiol. 2010;76 1:48-67. doi:10.1111/j.1365-2958.2010.07062.x.

55. Carranza PG, Gargantini PR, Prucca CG, Torri A, Saura A, Svard S, et al. Specific histone modifications play critical roles in the control of encystation and antigenic variation in the early-branching eukaryote *Giardia lamblia*. Int J Biochem Cell Biol. 2016;81 Pt A:32-43. doi:10.1016/j.biocel.2016.10.010.

56. Lalle M, Camerini S, Cecchetti S, Sayadi A, Crescenzi M and Pozio E. Interaction network of the 14-3-3 protein in the ancient protozoan parasite *Giardia duodenalis*. J Proteome Res. 2012;11 5:2666-83. doi:10.1021/pr3000199.

57. Gourguechon S, Holt LJ and Cande WZ. The Giardia cell cycle progresses independently of the anaphase-promoting complex. J Cell Sci. 2013;126 Pt 10:2246-55. doi:10.1242/jcs.121632.

58. Lourenco D, Andrade Ida S, Terra LL, Guimaraes PR, Zingali RB and de Souza W. Proteomic analysis of the ventral disc of *Giardia lamblia*. BMC Res Notes. 2012;5:41. doi:10.1186/1756-0500-5-41.

59. Muller J, Rout S, Leitsch D, Vaithilingam J, Hehl A and Muller N. Comparative characterisation of two nitroreductases from *Giardia lamblia* as potential activators of nitro compounds. Int J Parasitol Drugs Drug Resist. 2015;5 2:37-43. doi:10.1016/j.ijpddr.2015.03.001.

60. Tan J, Jakob U and Bardwell JC. Overexpression of two different GTPases rescues a null mutation in a heat-induced rRNA methyltransferase. J Bacteriol. 2002;184 10:2692-8.

61. Bugl H, Fauman EB, Staker BL, Zheng F, Kushner SR, Saper MA, et al. RNA methylation under heat shock control. Mol Cell. 2000;6 2:349-60.

62. Morris G, Walder K, Puri BK, Berk M and Maes M. The Deleterious Effects of Oxidative and Nitrosative Stress on Palmitoylation, Membrane Lipid Rafts and Lipid-

Based Cellular Signalling: New Drug Targets in Neuroimmune Disorders. Mol Neurobiol. 2015; doi:10.1007/s12035-015-9392-y.

63. Stark G. Functional consequences of oxidative membrane damage. J Membr Biol. 2005;205 1:1-16. doi:10.1007/s00232-005-0753-8.

64. Serran-Aguilera L, Denton H, Rubio-Ruiz B, Lopez-Gutierrez B, Entrena A, Izquierdo L, et al. *Plasmodium falciparum* Choline Kinase Inhibition Leads to a Major Decrease in Phosphatidylethanolamine Causing Parasite Death. Sci Rep. 2016;6:33189. doi:10.1038/srep33189.

65. Steels EL, Learmonth RP and Watson K. Stress tolerance and membrane lipid unsaturation in *Saccharomyces cerevisiae* grown aerobically or anaerobically. Microbiology. 1994;140 (Pt 3):569-76. doi:10.1099/00221287-140-3-569.

66. Ellis JE, Wyder MA, Jarroll EL and Kaneshiro ES. Changes in lipid composition during in vitro encystation and fatty acid desaturase activity of *Giardia lamblia*. Mol Biochem Parasitol. 1996;81 1:13-25.

67. Tom CT and Martin BR. Fat chance! Getting a grip on a slippery modification. ACS Chem Biol. 2013;8 1:46-57. doi:10.1021/cb300607e.

68. Burgoyne JR, Haeussler DJ, Kumar V, Ji Y, Pimental DR, Zee RS, et al. Oxidation of HRas cysteine thiols by metabolic stress prevents palmitoylation *in vivo* and contributes to endothelial cell apoptosis. FASEB J. 2012;26 2:832-41. doi:10.1096/fj.11-189415.

69. Touz MC, Conrad JT and Nash TE. A novel palmitoyl acyl transferase controls surface protein palmitoylation and cytotoxicity in *Giardia lamblia*. Mol Microbiol. 2005;58 4:999-1011. doi:10.1111/j.1365-2958.2005.04891.x.

70. Humen MA, Perez PF and Lievin-Le Moal V. Lipid raft-dependent adhesion of *Giardia intestinalis* trophozoites to a cultured human enterocyte-like Caco-2/TC7 cell monolayer leads to cytoskeleton-dependent functional injuries. Cell Microbiol. 2011;13 11:1683-702. doi:10.1111/j.1462-5822.2011.01647.x.

71. De Chatterjee A, Mendez TL, Roychowdhury S and Das S. The assembly of GM1 glycolipid- and cholesterol-enriched raft-like membrane microdomains is important for giardial encystation. Infect Immun. 2015;83 5:2030-42. doi:10.1128/IAI.03118-14.

72. Prucca CG and Lujan HD. Antigenic variation in *Giardia lamblia*. Cell Microbiol. 2009;11 12:1706-15. doi:10.1111/j.1462-5822.2009.01367.x.

73. Ma'ayeh SY, Knorr L and Svard SG. Transcriptional profiling of *Giardia intestinalis* in response to oxidative stress. Int J Parasitol. 2015;45 14:925-38. doi:10.1016/j.ijpara.2015.07.005.

74. Brown DM, Upcroft JA and Upcroft P. Cysteine is the major low-molecular weight thiol in *Giardia duodenalis*. Mol Biochem Parasitol. 1993;61 1:155-8.

75. Wouters MA, Fan SW and Haworth NL. Disulfides as redox switches: from molecular mechanisms to functional significance. Antioxid Redox Signal. 2010;12 1:53-91. doi:10.1089/ARS.2009.2510.

76. Woycechowsky KJ and Raines RT. The CXC motif: a functional mimic of protein disulfide isomerase. Biochemistry. 2003;42 18:5387-94. doi:10.1021/bi026993q.

77. Paulech J, Solis N and Cordwell SJ. Characterization of reaction conditions providing rapid and specific cysteine alkylation for peptide-based mass spectrometry. Biochim Biophys Acta. 2013;1834 1:372-9. doi:10.1016/j.bbapap.2012.08.002.

78. Paulech J, Solis N, Edwards AV, Puckeridge M, White MY and Cordwell SJ. Large-scale capture of peptides containing reversibly oxidized cysteines by thiol-disulfide exchange applied to the myocardial redox proteome. Anal Chem. 2013;85 7:3774-80. doi:10.1021/ac400166e.

79. Faso C, Bischof S and Hehl AB. The proteome landscape of *Giardia lamblia* encystation. PloS one. 2013;8 12:e83207. doi:10.1371/journal.pone.0083207.

80. Nash TE, Banks SM, Alling DW, Merritt JW, Jr. and Conrad JT. Frequency of variant antigens in *Giardia lamblia*. Exp Parasitol. 1990;71 4:415-21.

81. Nino CA, Chaparro J, Soffientini P, Polo S and Wasserman M. Ubiquitination dynamics in the early-branching eukaryote *Giardia intestinalis*. Microbiologyopen. 2013;2 3:525-39. doi:10.1002/mbo3.88.

82. Kang WK, Kim YH, Kim BS and Kim JY. Growth phase-dependent roles of Sir2 in oxidative stress resistance and chronological lifespan in yeast. J Microbiol. 2014;52 8:652-8. doi:10.1007/s12275-014-4173-2.

83. Qiu X, Brown K, Hirschey MD, Verdin E and Chen D. Calorie restriction reduces oxidative stress by SIRT3-mediated SOD2 activation. Cell Metab. 2010;12 6:662-7. doi:10.1016/j.cmet.2010.11.015.

84. Tao R, Coleman MC, Pennington JD, Ozden O, Park SH, Jiang H, et al. Sirt3-mediated deacetylation of evolutionarily conserved lysine 122 regulates MnSOD activity in response to stress. Mol Cell. 2010;40 6:893-904. doi:10.1016/j.molcel.2010.12.013.

85. Luján HD and Svärd S. *Giardia*: A model organism. Springer Science & Business Media; 2011.

86. Choudhary C, Kumar C, Gnad F, Nielsen ML, Rehman M, Walther TC, et al. Lysine acetylation targets protein complexes and co-regulates major cellular functions. Science. 2009;325 5942:834-40. doi:10.1126/science.1175371.

87. Choudhary C, Weinert BT, Nishida Y, Verdin E and Mann M. The growing landscape of lysine acetylation links metabolism and cell signalling. Nat Rev Mol Cell Biol. 2014;15 8:536-50. doi:10.1038/nrm3841.

88. Cobbold SA, Santos JM, Ochoa A, Perlman DH and Llinas M. Proteome-wide analysis reveals widespread lysine acetylation of major protein complexes in the malaria parasite. Sci Rep. 2016;6:19722. doi:10.1038/srep19722.

89. Fisk JC and Read LK. Protein arginine methylation in parasitic protozoa. Eukaryot Cell. 2011;10 8:1013-22. doi:10.1128/EC.05103-11.

90. Cherblanc FL, Chapman KL, Brown R and Fuchter MJ. Chaetocin is a nonspecific inhibitor of histone lysine methyltransferases. Nat Chem Biol. 2013;9 3:136-7. doi:10.1038/nchembio.1187.

91. Zhang X, Wen H and Shi X. Lysine methylation: beyond histones. Acta Biochim Biophys Sin (Shanghai). 2012;44 1:14-27. doi:10.1093/abbs/gmr100.

92. Chen N, Upcroft JA and Upcroft P. A *Giardia duodenalis* gene encoding a protein with multiple repeats of a toxin homologue. Parasitology. 1995;111 (Pt 4):423-31.

93. Upcroft JA, Healey A, Murray DG, Boreham PF and Upcroft P. A gene associated with cell division and drug resistance in *Giardia duodenalis*. Parasitology. 1992;104 (Pt 3):397-405.

94. Townson SM, Hanson GR, Upcroft JA and Upcroft P. A purified ferredoxin from *Giardia duodenalis*. Eur J Biochem. 1994;220 2:439-46.

95. Yee J, Tang A, Lau WL, Ritter H, Delport D, Page M, et al. Core histone genes of *Giardia intestinalis*: genomic organization, promoter structure, and expression. BMC Mol Biol. 2007;8:26. doi:10.1186/1471-2199-8-26.

96. Salusso A, Zlocowski N, Mayol GF, Zamponi N and Ropolo AS. Histone methyltransferase 1 regulates the encystation process in the parasite *Giardia lamblia*. FEBS J. 2017; doi:10.1111/febs.14131.

97. Martin C and Zhang Y. The diverse functions of histone lysine methylation. Nat Rev Mol Cell Biol. 2005;6 11:838-49. doi:10.1038/nrm1761.

98. Kim GH, Ryan JJ and Archer SL. The role of redox signaling in epigenetics and cardiovascular disease. Antioxid Redox Signal. 2013;18 15:1920-36. doi:10.1089/ars.2012.4926.

99. Wessel D and Flugge UI. A method for the quantitative recovery of protein in dilute solution in the presence of detergents and lipids. Anal Biochem. 1984;138 1:141-3.

100. Huang da W, Sherman BT and Lempicki RA. Systematic and integrative analysis of large gene lists using DAVID bioinformatics resources. Nature protocols. 2009;4 1:44-57. doi:10.1038/nprot.2008.211.

101. Szklarczyk D, Morris JH, Cook H, Kuhn M, Wyder S, Simonovic M, et al. The STRING database in 2017: quality-controlled protein-protein association networks, made broadly accessible. Nucleic Acids Res. 2017;45 D1:D362-D8. doi:10.1093/nar/gkw937.

102. Boreham PF, Phillips RE and Shepherd RW. The sensitivity of *Giardia intestinalis* to drugs *in vitro*. J Antimicrob Chemother. 1984;14 5:449-61.

103. Touz MC, Ropolo AS, Rivero MR, Vranych CV, Conrad JT, Svard SG, et al. Arginine deiminase has multiple regulatory roles in the biology of *Giardia lamblia*. J Cell Sci. 2008;121 Pt 17:2930-8. doi:10.1242/jcs.026963.

104. Weiland ME, Palm JE, Griffiths WJ, McCaffery JM and Svard SG. Characterisation of alpha-1 giardin: an immunodominant *Giardia lamblia* annexin with glycosaminoglycan-binding activity. Int J Parasitol. 2003;33 12:1341-51.

105. Pathuri P, Nguyen ET, Svard SG and Luecke H. Apo and calcium-bound crystal structures of Alpha-11 giardin, an unusual annexin from *Giardia lamblia*. J Mol Bio. 2007;368 2:493-508. doi:10.1016/j.jmb.2007.02.016.

106. Pathuri P, Nguyen ET, Ozorowski G, Svard SG and Luecke H. Apo and calcium-bound crystal structures of cytoskeletal protein alpha-14 giardin (annexin E1) from the intestinal protozoan parasite *Giardia lamblia*. J Mol Bio. 2009;385 4:1098-112. doi:10.1016/j.jmb.2008.11.012.

107. Saric M, Vahrmann A, Niebur D, Kluempers V, Hehl AB and Scholze H. Dual acylation accounts for the localization of {alpha}19-giardin in the ventral flagellum pair of *Giardia lamblia*. Eukaryot Cell. 2009;8 10:1567-74. doi:10.1128/EC.00136-09.

108. Emery SJ, Baker L, Ansell BR, Mirzaei M, Haynes PA, McConville MJ et al. Supporting data for 'Differential protein expression and post-translational

modifications in Metronidazole-resistant Giardia duodenalis'. *GigaScience* database 2018. http://dx.doi.org/10.5524/100416

Figure Legends:

Figure 1: **Protein identification, differential expression and functional enrichment in Mtz lines. A)** Proportional Venn diagrams showing unique and overlapping protein identifications in the three TMT 10plexes (left) and for differentially expressed proteins in each MtzR line compares to MtzS parents (right). **B)** The six functional clusters identified as enriched from differentially expressed proteins identified in MtzR isolates and their total protein number.

Figure 2: **EGF-like differentially expressed proteins and VSP subpopulations. A)** Distribution of differentially-expressed, EGF-like proteins within *Giardia* protein families by MtzR line. **B)** Proportional Venn diagrams showing overlapping identities of all differentially expressed EGF-like proteins in MtzR lines (above) and all differentially expressed VSP proteins (below). **C)** Heatmap showing fold change in expression of VSPs in MtzR lines compared to the MtzS parents, replicate details are shown on the bottom axis to represent biological variation within lines. Lower fold changes are represented by blue, while higher fold changes are represented in red.

Figure 3: **Protein expression in antioxidant and electron transport networks.** Gene annotations including accension numbers (prefix ‘GL50803_’) and average protein expression fold change between MtzR from MtzS parents in **A)** antioxidant proteins and **B)** electron transport proteins.

Figure 4: **Differential protein expression in glycolysis and pyruvate catabolism.** Pyruvate catabolism, with emphases on enzymes with electron transport in upstream glycolysis. Enzymes with a white background were not identified in the protein dataset but have been included for completeness of pathway. Metabolites are shown in black boxes. Direction of differential expression in proteins is indicated using arrows and colours as designated in the

top right corner. Fd, Ferredoxin; ADH LTC, alcohol dehydrogenase lateral transfer candidate; KB, Ketobutyrate; KG, Ketoglutarate.

Figure 5: **Western blots of post-translational protein modifications in MtzS and MtzR lines.** Total protein lysate from trophozoites (15µg) from WB, 713 and 106 MtzS and MtzR lines was probed with antibodies against acetylated lysine (KAc), mono-methylated lysine (K-MMe), ubiquitin (Ubi), phosphorylated tyrosine (pY), and the 14-3-3 binding motif (including phosphorylated serine) (14-3-3). Protein loading was verified after transfer using Ponceau S staining (first row, left). MtzS and MtzR lanes are designated by a 'S' and 'R' respectively. Altered protein features detected in 3/3 MtzR lines are designated with a solid red arrow, while protein features changed in 2/3 lines are designated with a broken red arrow.

Figure 6: **Results of Trichostatin A exposure in MtzS and MtzR lines. A)** Dose response curves for MtzR and MtzS lines of WB, 106 and 713 to deacetylase inhibitor TSA. Error bars represent ± 1 standard deviation and experiments were performed in triplicate. **B)** Western blots for total protein lysate from trophozoites (10µg) exposed to TSA from WB, 713 and 106 MtzS and MtzR lines probed with antibodies against acetylated lysine (KAc). Trophozoites were exposed for 18 hours to 2µM TSA, with control flasks exposed to the same volume DMSO as used as a vehicle for TSA exposure. Red arrows designate protein features with significant changes between TSA exposed and control trophozoites in the six lines.

Figure 7: **IC_{50} profiles and post-translational modifications upon discontinuation of Mtz selection. A)** Dose response curves for MtzR lines of 106 and 713 upon discontinuation of drug selection at 4 (P8), 8 (P16) and 12 (P24) weeks as compared to MtzS lines. MtzS lines were used and are designated 106_WT and 713_WT. Error bars represent ± 1 standard deviation and experiments were performed in triplicate. The table below records the calculated IC_{50} for each timepoint, with resistance factors calculated against the IC_{50} of the MtzS parent isolate. **B)** Western blots against lysate from trophozoites (15µg) from 713 and 106-MtzR (P0) and 4 (P8), 8 (P16) after discontinued drug selection was probed with antibodies against acetylated lysine (KAc), mono-methylated lysine (K-Mme) and phosphorylated tyrosine (pY). Altered features within 713 and 106 lines are designated on the right of the blot using a solid red arrow.

Exposure times have been reduced by 20% to prevent overexposure of major bands (e.g histone variants).

Tables:

Table 1: IC_{50} and resistance factor (RF) for metronidazole in the three isogenic isolates utilised in this study.

Isolate	Strain	Abbreviation	References	Mtz IC_{50}	RF
**WB**	WB1B	WB-MtzS	[26]	8.28μM	-
	WB1B-M3	WB-MtzR	[31]	22.79μM	2.8
**106**	106	106-MtzS	[102]	9.39μM	-
	106-2ID10	106-MtzR	[30]	23.99μM	2.6
**713**	713	713-MtzS	[26]	7.79μM	-
	713-M3	713-MtzR	[31]	42.33μM	5.4

Table 2: Summary of protein identification, differentially expressed proteins and protein quantitation FDR for the dataset. Proteins were considered differentially expressed if proteins were statistically significant (p-value ≤ 0.05) and met ratio fold change cutoffs for up-regulation (≥ 1.3) or down-regulation (≤0.77).

	**TMT1** *(WB-MTZR V MTZS)*	**TMT2** *(106-MTZR V MTZS)*	**TMT2** *(713-MTZR V MTZS)*
**# Protein IDs**	1220	1126	1060
**# Non-Redundant Peptides**	9684	8692	6349
**# Differentially Expressed Proteins**	264	171	76
**# Up-regulated Proteins**	128	87	39
**# Down-regulated Proteins**	137	84	37

Table 3: Differentially expressed membrane protein families in MtzR lines, including subgroups and protein and annotation features along with overall differential expression (DE) trends oin MtzR lines. ORFs listed in the last column were observed to be differentially expressed in at least 2/3 MtzR lines.

Group	Subgroup	Membrane Association			DE Trends	Key DE ORFs
		TMH[a]	Extra/Intracellular domains	Additional Features		
EGF-Like Proteins	VSP	Yes	CXXC-rich extracellular<br>CRGKA cytoplasmic tail	S-Palmitoyl necessary for lipid raft localization and lipid signaling [69]<br>Citrullination of arginine [103]	Large DE in terms of proportion and magnitude, little specific variant overlap and variable directionality.	GL50803_137620<br>GL50803_37093
	HCMP	Yes	CXC/CXXC extracellular	Possible organelle membrane localization.	Several highly up-/down-regulated variants, little specific variant overlap	GL50803_112673<br>GL50803_112633
	Tenascin/Notch-like	Yes	EGF-like conserved site (IPR013032); usually extracellular	Some possess IPR013111 (EGF-like domain, extracellular)	DE with varying directionality between lines.	GL50803_11420<br>GL50803_16322
Peptidases	Cysteine-rich	Yes*	Growth factor receptor cysteine-rich domain (IPR009030)	Separate from VSP/HCMP, do not contain IPR005127 or IPR006212 annotations.	Down-regulation	GL50803_14225<br>GL50803_101832
	Dipeptidyl-peptidases	Yes*	Serine-type (GO: 0008236), cysteine-type (GO:0008234) or dipeptidyl-peptidase activity (GO:0008239)	Can localize to plasma membrane in absence of TMH [49]<br>Possible role in signal transduction, particularly during encystation [49]	Dipeptidyl-peptidase III up-regulation (106).<br>Alanyl dipeptidyl peptidase down-regulation (713, WB)<br>Dipeptidyl-peptidase I down-regulation (WB).	GL50803_15574
ABC Transporters	Plasma Membrane	Yes	IPR017871 (ABC transporter, conserved site)	Features some lipid-transporting ATPases.	DE with varying directionality between lines.	GL50803_115052<br>GL50803_16592
Giardins	Alpha-Giardins	No	Calcium-depending phospholipid binding (GO:0005544)	Localize to membrane and cytoskeletal structures, including as flagellar [52, 104-106].<br>Evidence for dual acylation [107]	Down-regulation	
	Beta-Giardin	No	Cytoskeletal	Ventral disc localization [53]	Down-regulation	GL50803_4812

[a] Entries represented with a '*' indicates some, but not all members, possess annotated TMH.

Supplementary Information:

Supplementary Data S1: Protein and peptide identifications, TMT reporter ion ratios and protein quantitation and p-value significance. Supplementary for TMT1 (WB MtzS vs MtzR) is presented on the first tab, TMT2 (106 MtzS vs MtzR) is presented on the second tab and TMT3 (713 MtzS vs MtzR) is presented on the third tab. These include GL50803_ identifiers, converted 'Entrez Gene IDs' for DAVID bioinformatic functional analyses, the geometric mean for fold change and the T-rest p-value of significance. A significant p-value (< 0.05) has been highlighted in yellow. Proteins above the up-regulated fold change cutoff are highlighted in red, while proteins below the fold change for down-regulation are highlighted in green.

Supplementary Data S2: Functional annotation for differentially expressed proteins in MtzR lines. Supplementary for TMT1 (WB MtzS vs MtzR) is presented on the first tab, TMT2 (106 MtzS vs MtzR) is presented on the second tab and TMT3 (713 MtzS vs MtzR) is presented on the third tab. Each supplementary tab shows the functional annotation, including for GO, Interpro and SignalP for each of the DEPs which met p-value and fold change cutoffs for differential expression in each MtzR lines. A significant p-value (< 0.05) has been highlighted in yellow. Proteins above the up-regulated fold change cutoff are highlighted in red, while proteins below the fold change for down-regulation are highlighted in green.

Supplementary Data S3: DAVID functional annotation enrichment and clusters. The six enriched functional clusters are shown, including the MtzR lines in which enrichment was observed, and the GO annotation and/or Interpro domains around which the functional clusters were selected. The *Giardia* identifiers of each of the DEPs involved with the designated Interpro and GO annotations are listed within each of the three *Giardia* MtzR lines.

Supplementary Figure 1: A) Volcano plots illustrating the dual criteria for differentially expressed proteins. The x-axis represents log fold change with the vertical blue lines indicating 1.3 and 0.77 ratio, while the -log p value is plotted on the y-axis with proteins above the red horizontal line indicating significance ≤0.05. Each data point represents a single identified protein. Proteins within the upper and outer quadrants meet both the fold change and p-value cut-off, and are therefore considered as differentially expressed. **B)** PCA plots of principal component scores plot in the space

of the first three principal components generated for the whole dataset of log_2 ratios to the pooled control (label 131) with each distribution for sample groups highlighted. All channels relevant to MtzR and MtzS ratio calculated are highlighted in the plots, with triplicate channels for MtzS (DMSO Control) lines are shown in each of the three plots are shown in green, while the channels containing the MtzR replicates are highlighted in purple (WB-MtzR), red (106-MtzR) and blue (713-MtzR) **C)** P-value histograms showing the distribution of p-values from the paired t-tests comparing the MtzS samples respectively to MtzR.The p-value histograms have a peak corresponding to a larger number of low p-values, which is indicative of a real underlying effect; a random or noisy dataset is expected to generate a uniform distribution of p-values and hence a flat histogram.

Supplementary Figure 2: Protein-RNA log^2 fold change correlations plots by genotype for all proteins quantified by TMT label ratios. Fold change was dervied from ratios of MtzR replicated over their MtzS line, and then log^2 transformed. The corresponding RNA log^2 fold change was derived from transcript expression data from Ansell *et al* [18]. Correlation between protein and RNA abundance fold changes were calculated at r^2= 0.154 for WB, r^2= 0.105 for WB and r^2= 0.187 for WB ($p < 0.01$) for genes identified in both datasets.

Supplementary Figure 3: Proportional Venn diagrams showing the number of differentially expressed proteins relative to protein identifications across the three TMT experiments to compare MtzS and MtzR isolates. Proportional Venn diagrams demonstrate that the low overlap between differentially expressed proteins between lines (Figure 1), is not due to discrepancies in identifications between experiments.

Supplementary Figure 4: Protein interactome network for DEPs in WB MtzR (Top), 106 MtzR (Middle) and 713 MtzR (Bottom). Using the STRING software, proteins are represented with nodes and the interactions with continuous lines to represent direct interactions (physical), while indirect ones (functional) are presented by interrupted lines. Thickness of the line is to represent the strength of the data support of interaction evidence between network nodes. Colouring is performed according to MCL clustering. The coloured background and overarching functional annotations have been added to highlight protein-protein interaction networks of multiple nodes with direct interactions among DEPs in the MtzR lines.

Supplementary Figure 5: Total protein lysate from trophozoites (10µg) from WB, 713 and 106 wild type lines was probed with antibodies against the H3 variant (top) and H4 variant (bottom), as well as probed with antibodies again acetylated lysine (KAc) and mono-methylated lysine (K-MMe). Blots have been cropped to 25-10kDa. The H3 variant (~17kDa) and the H4 variant (~11kDa) bands correspond to the position of the prominent modified protein bands on KAc and KMme blots, mostly likely the acetylation and methylation histone marks on the H3 and H4 variants.

Supplementary Figure 6: Dose-response curves for MtzS lines (dark colors, solid line) and MtzR lines (light colors, dotted lines) for chemical inhibitors of protein post-translational modification networks. Experiments performed in biological triplicate. Error bars represent ± 1 standard deviation. Table below dose response curves show the IC_{50} concentration for each line, and the resistance factor is calculated for MtzR lines relative to their MtzS parent. Trichostatin A (grey fill) had a significantly increased IC_{50} in all MtzR lines relative to their MtzS parents. Due to the high IC_{50} in Chaetocin, and the limits of solubility, it was difficult to plot the dose response curve for this compound and the IC_{50} was approximately estimated in some lines.

Supplementary Figure 7: Total protein lysate (10µg) from trophozoites exposed for 8 hours to 1, 2 and 4µM TSA, with control flasks exposed to the same volume DMSO as used as a vehicle for TSA. Protein from WB, 713 and 106 MtzS and MtzR lines probed with antibodies again acetylated lysine (KAc) (below). Protein loading was verified after transfer using Ponceau S staining (upper). At 8 hours, MtzS isolates are beginning to show hyper-acetylation of H3 and H4 variants, which is not observed in MtzR lines. Based on this optimisation experiment, 2µM of TSA was chosen as a concentration for a longer TSA exposure timecourse in MtzR and MtzS lines.

Supplementary Figure 8: Light microscope images of trophozoite in MtzS and MtzR lines adhered to the flat-sided 10 mL tubes at 72hrs confluence prior to passage. Wild-type MtzS lines have reached complete confluence, while MyzR lines have varying degrees of reduced confluence and adherence as compared to MtzS parents.

Supplementary Figure 9: Growth improvements in MtzR lines during *in vitro* culture upon discontinuation of drug selection. Isolates were subcultured twice weekly to ensure consistent culture conditions, media availability and time in culture between the two lines (106-MtzR, 713-MtzR). Left axis shows the fold increase relative to MtzS parents in seed volume required for MtzR lines to compensates for lower growth rates/growth defects, while the right axis reflects the % confluence of adhered trophozoites averaged across the two subculture passages for that week. During Mtz selection in week 0, lower confluence and higher seed values were required. Upon discontinuation of drug selection, confluence and growth improved, and lower seed volumes required.

Figure 1

Click here to download Figure Emery et al, Figure 1.pdf

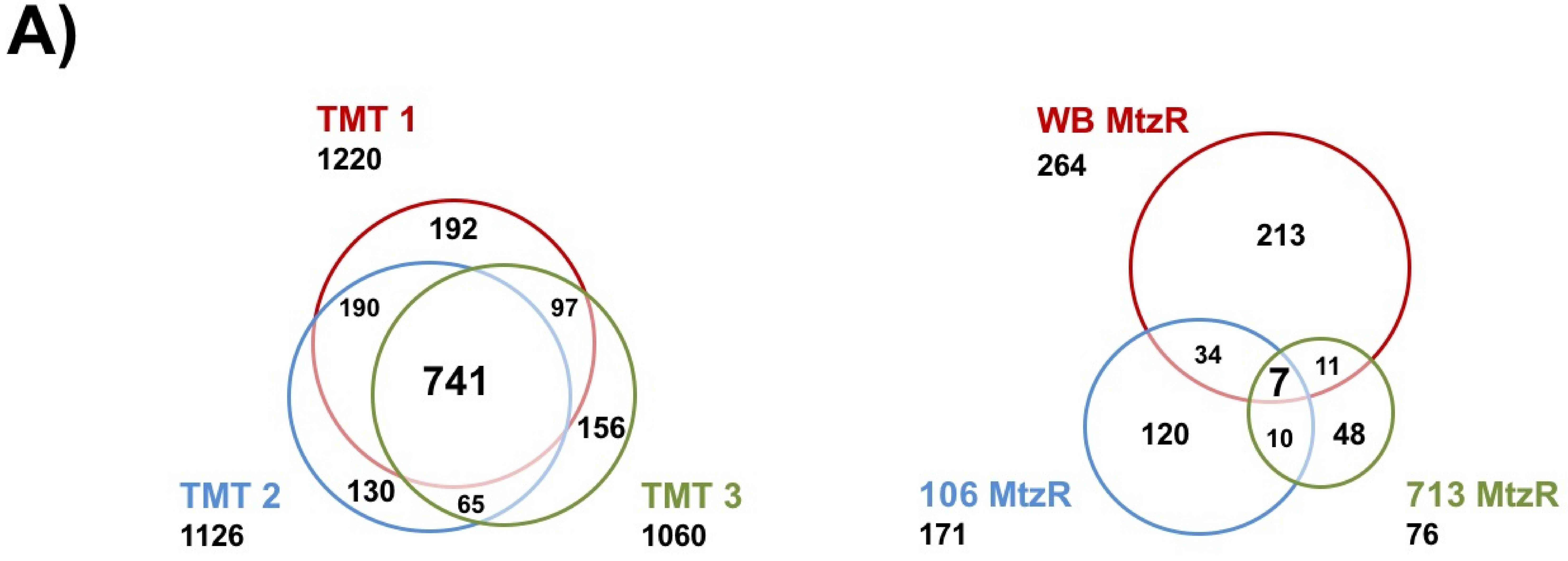

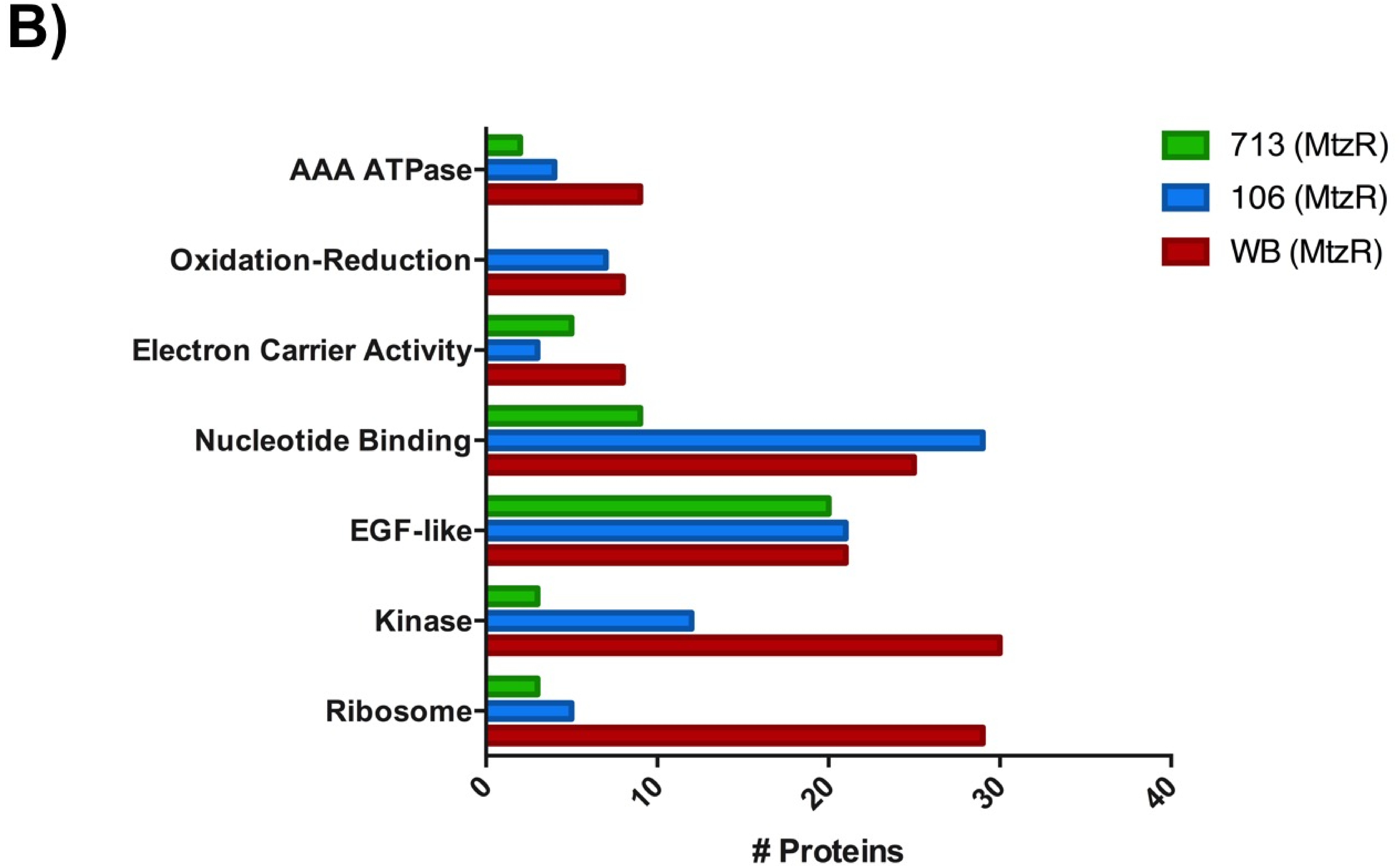

Figure 2
Click here to download Figure Emery et al, Figure 2.pdf

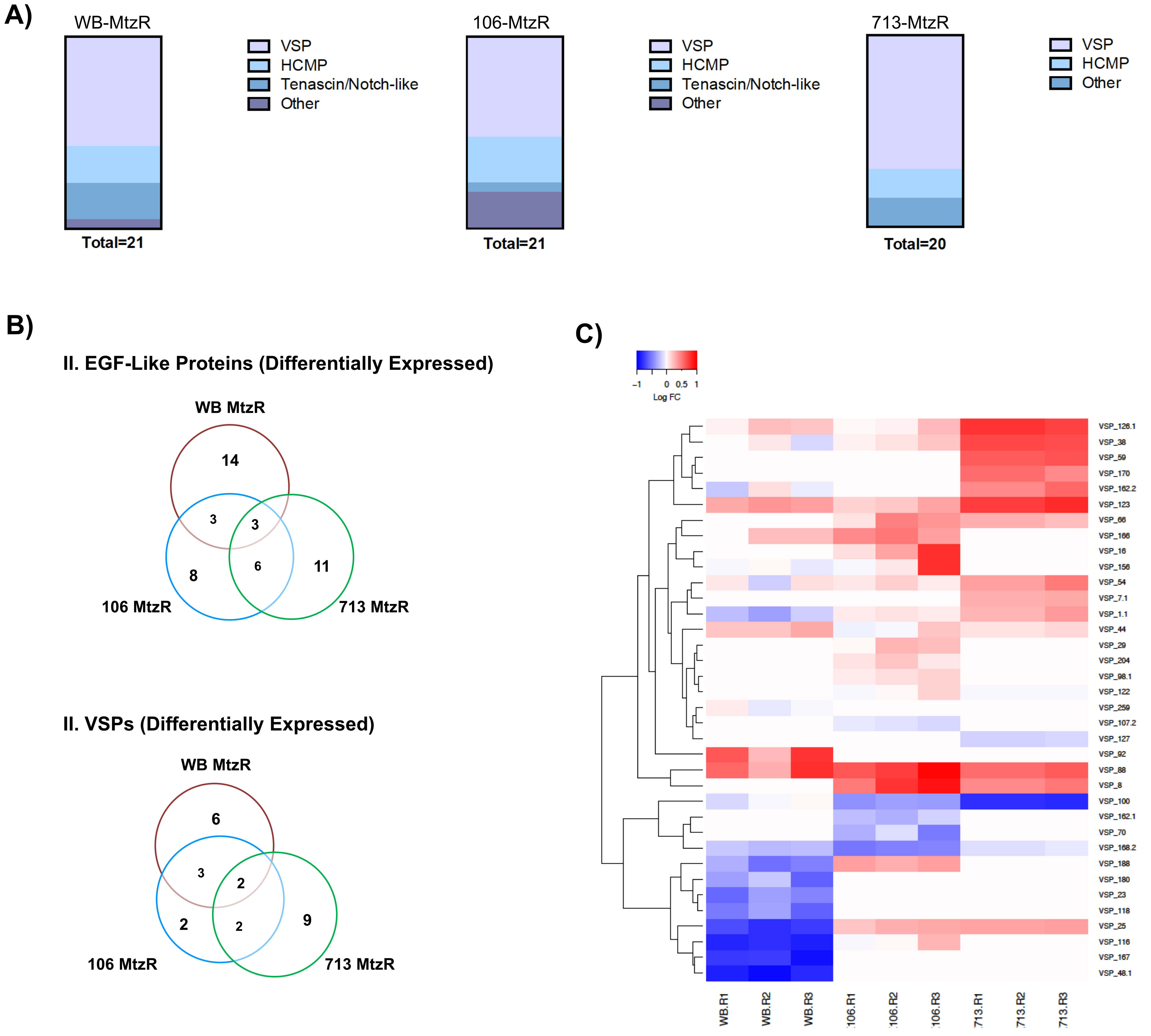

Click here to download Figure Emery et al, Figure 3.pdf

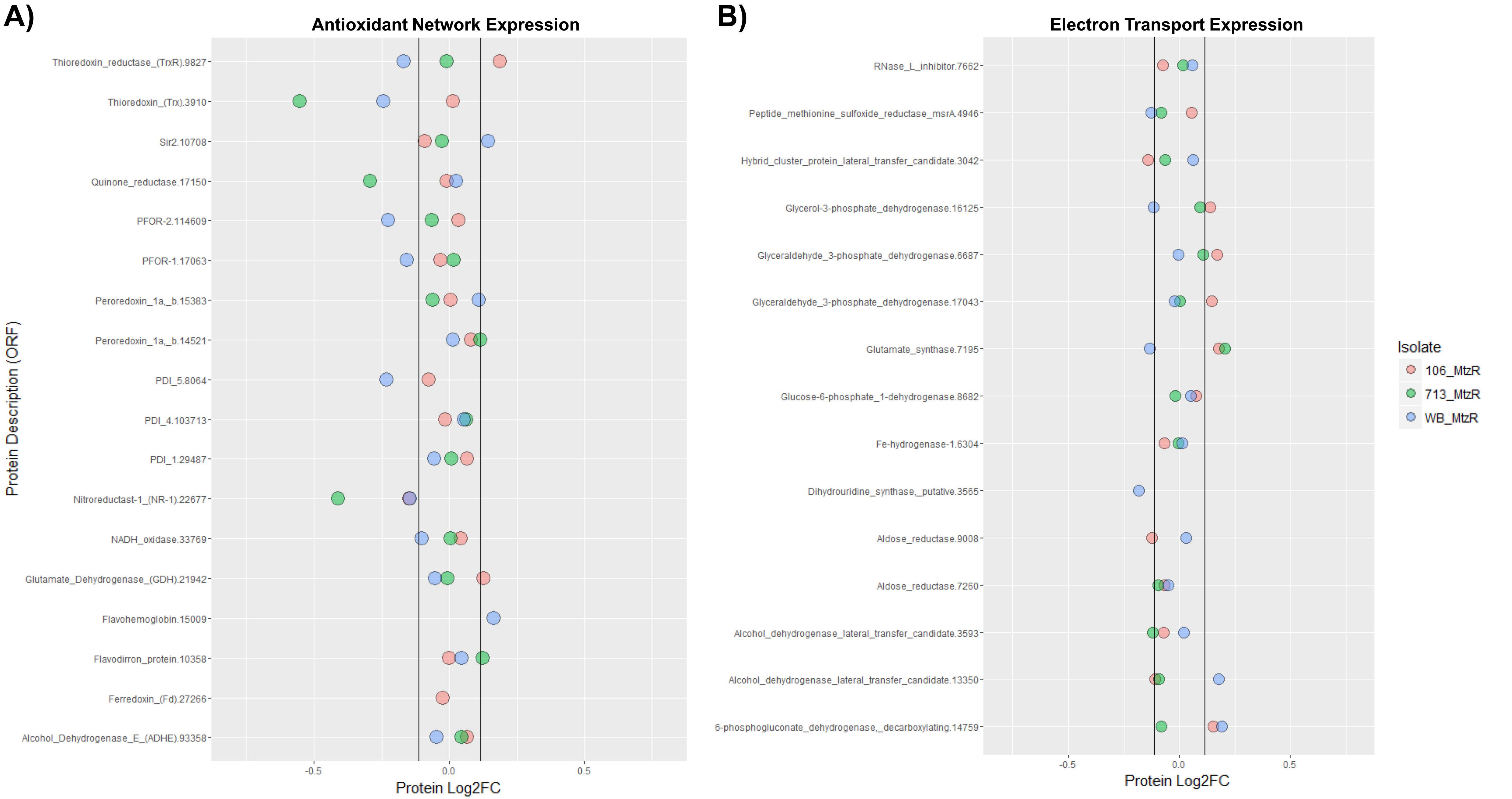

Figure 4

Click here to download Figure Emery et al. Figure 4.pdf

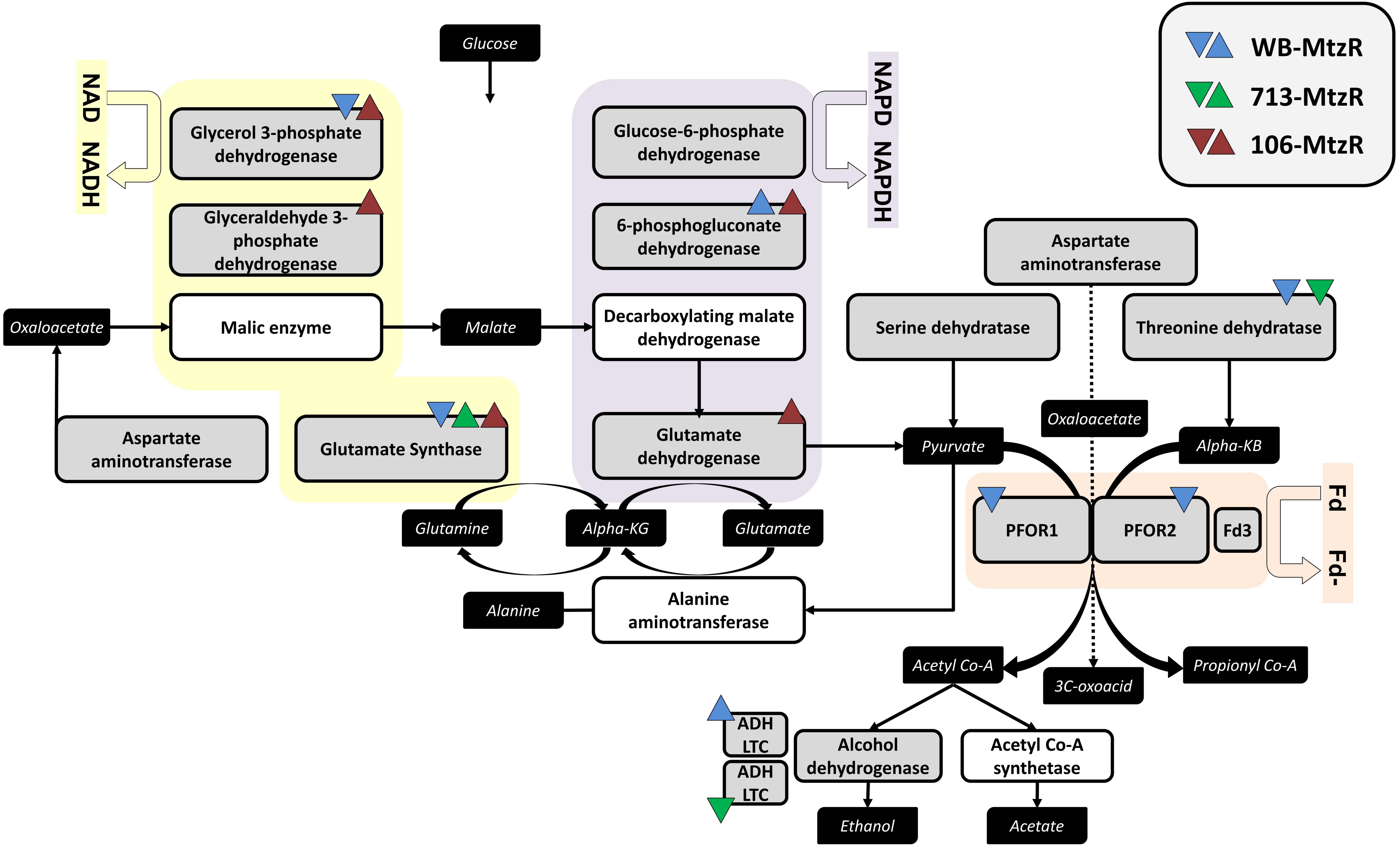

Click here to download Figure Emery et al, Figure 5.pdf

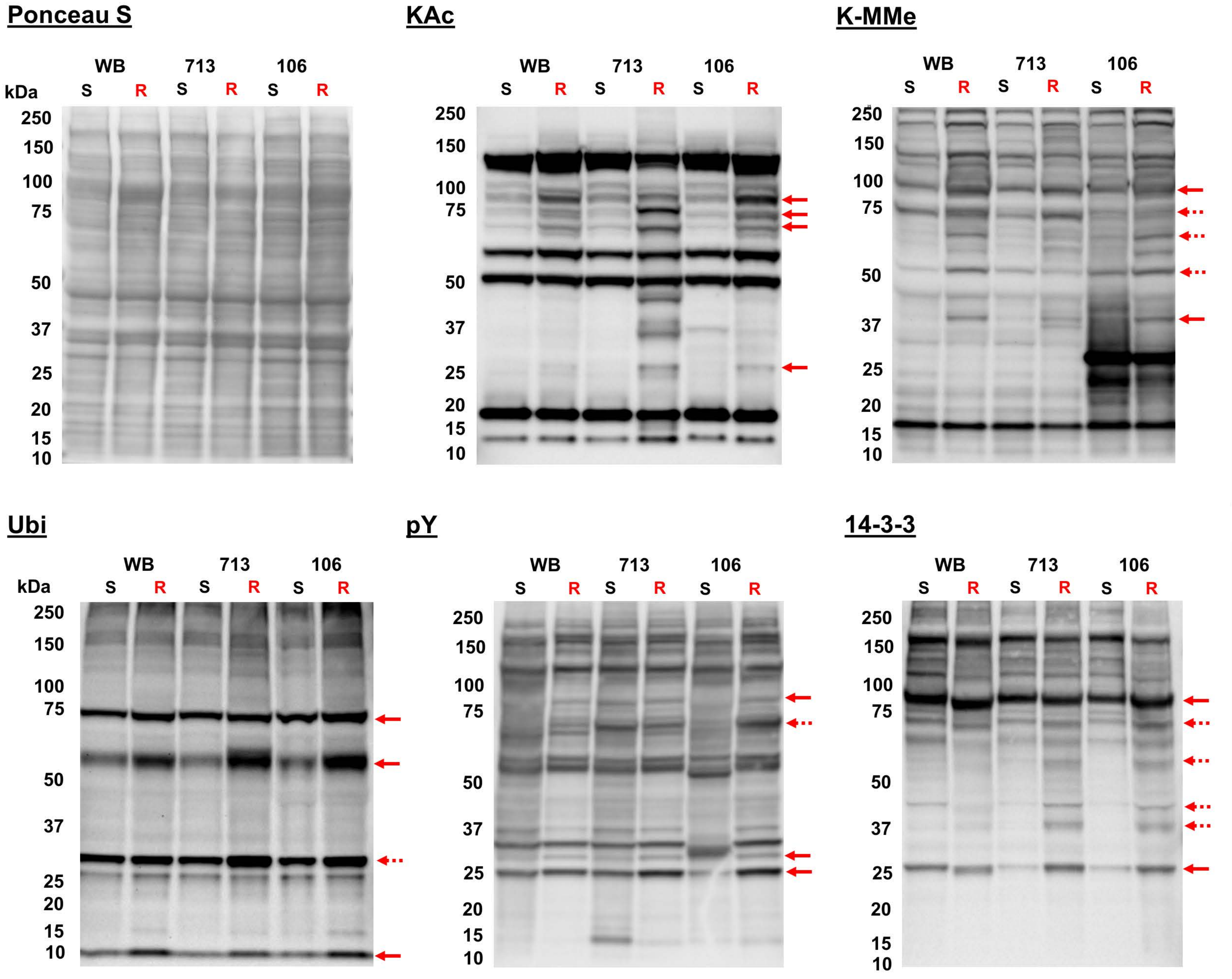

Figure 6

Click here to download Figure Emery et al, Figure 6.pdf

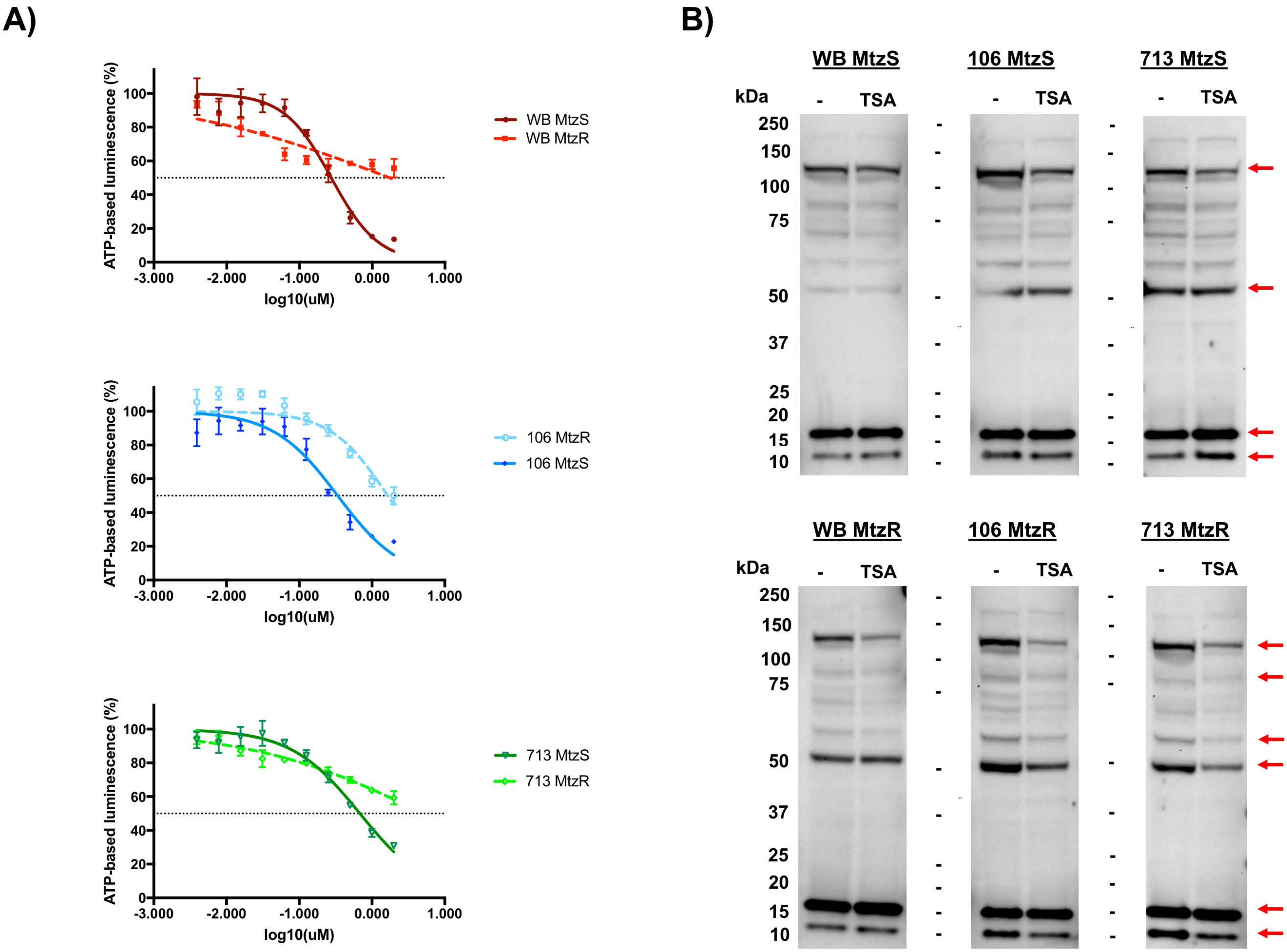

Figure 7
Click here to download Figure Emery et al, Figure 7.pdf

A)

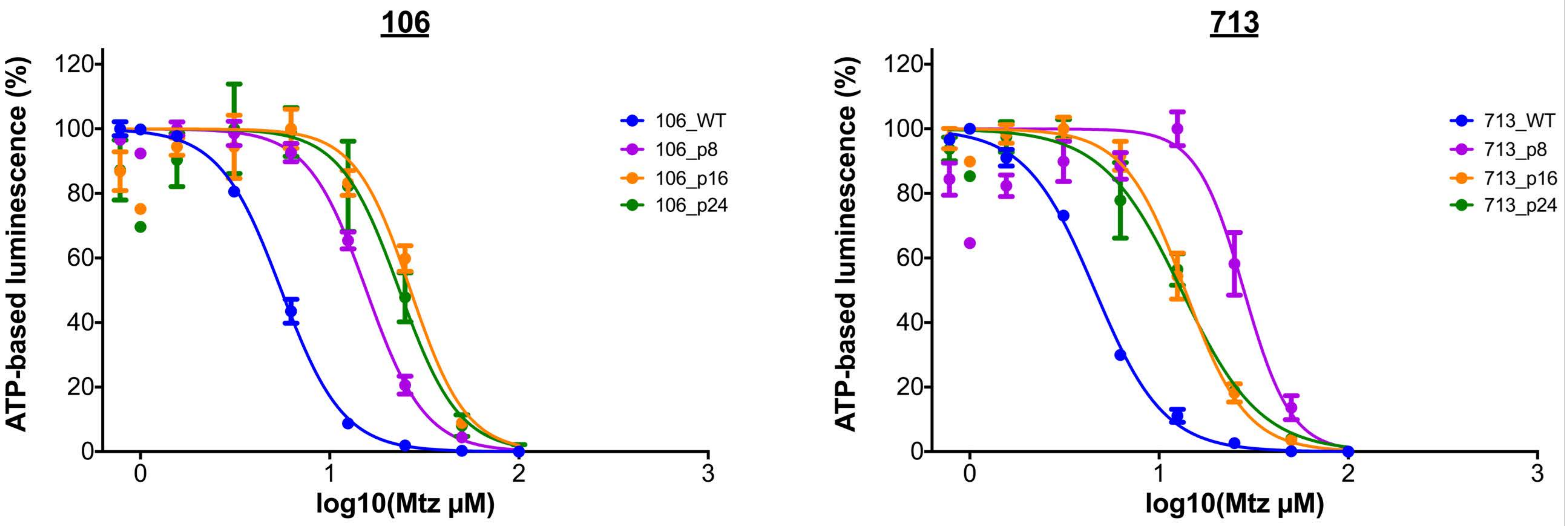

	WT	P8		P16		P24	
	Mtz IC_{50} (μM)	Mtz IC_{50} (μM)	RF	Mtz IC_{50} (μM)	RF	Mtz IC_{50} (μM)	RF
**106**	5.5	15.6	2.8	26.7	4.8	23.4	4.2
**713**	4.6	28.1	6.1	13.7	3.0	13.1	2.9

B)

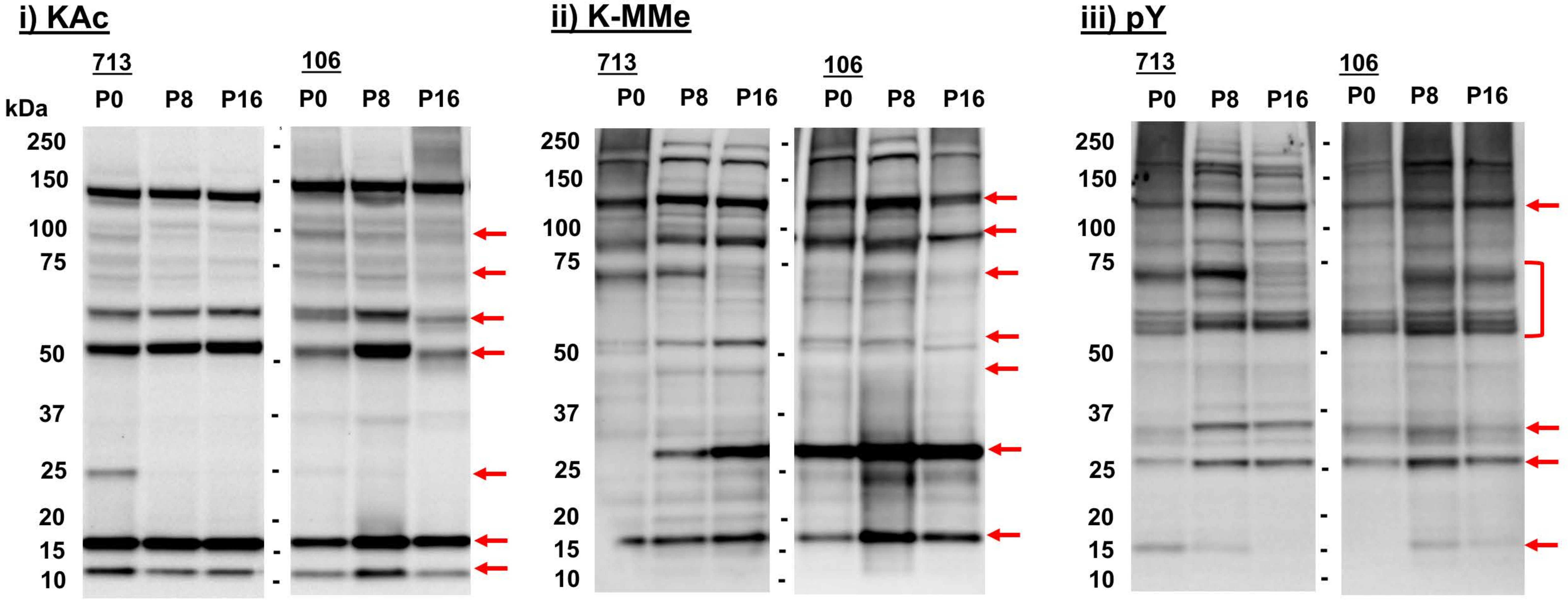

Supplementary Figure 1

Click here to access/download
Supplementary Material
Emery et al, Supplementary Figure 1.pdf

Supplementary Figure 2

Click here to access/download
Supplementary Material
Emery et al, Supplementary Figure 2.pdf

Supplementary Figure 3

Click here to access/download
Supplementary Material
Emery et al, Supplementary Figure 3.pdf

Supplementary Figure 4

Click here to access/download
Supplementary Material
Emery et al, Supplementary Figure 4.pdf

Supplementary Figure 5

Click here to access/download
Supplementary Material
Emery et al, Supplementary Figure 5.pdf

Supplementary Figure 6

Click here to access/download
Supplementary Material
Emery et al, Supplementary Figure 6.pdf

Supplementary Figure 7

Click here to access/download
Supplementary Material
Emery et al, Supplementary Figure 7.pdf

Supplementary Figure 8

Click here to access/download

Supplementary Material

Emery et al, Supplementary Figure 8.pdf

Supplementary Figure 9

Click here to access/download
Supplementary Material
Emery et al, Supplementary Figure 9.pdf

Click here to access/download
Supplementary Material
Emery et al, Supplementary S1 -- TMT Protein IDs and Quant.xlsx

Click here to access/download
Supplementary Material
Emery et al, Supplementary S2 -- Functional Annotation DEPs.xlsx

Click here to access/download
Supplementary Material
Emery et al, Supplementary S3 -- Functional Enrichment DEPs.xlsx
