# Supplementary material for: Differential protein expression and post-translational modifications in metronidazole-resistant Giardia duodenalis
Source: Gigascience. 2018 Mar 13;7(4):giy024. doi: 10.1093/gigascience/giy024 (PMC5913674; doi:10.1093/gigascience/giy024)
Supplement: Supplemental material [file giy024_supp.zip › Emery et al, Supplementary Figure 1.pdf]

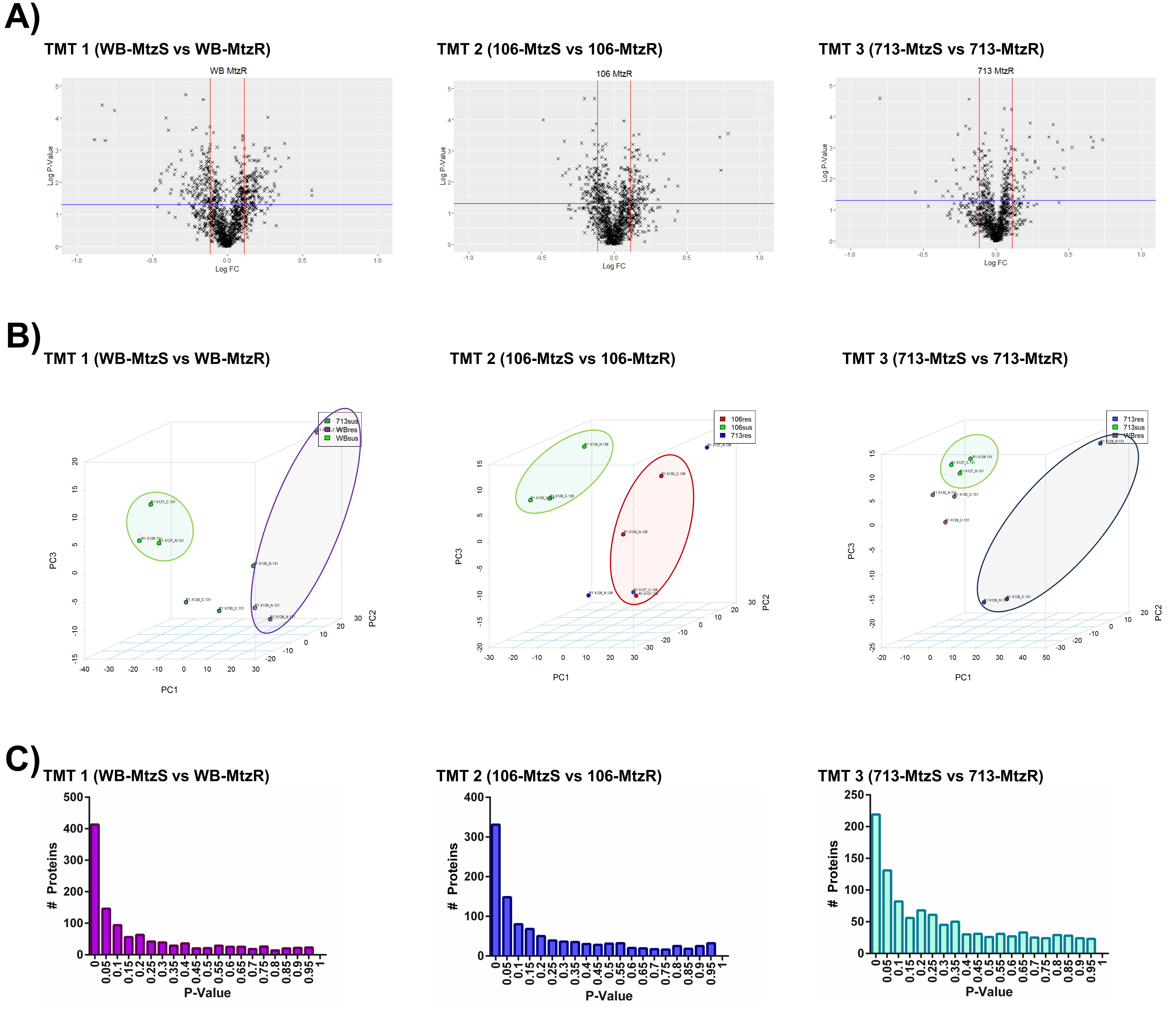
A)
TMT 1 (WB-MtzS vs WB-MtzR)
WB MtzR
Log P-Value
Log FC
TMT 2 (106-MtzS vs 106-MtzR)
106 MtzR
Log P-Value
Log FC
TMT 3 (713-MtzS vs 713-MtzR)
713 MtzR
Log P-Value
Log FC
B)
TMT 1 (WB-MtzS vs WB-MtzR)
713sus
WBres
WBsus
PC1
PC2
PC3
TMT 2 (106-MtzS vs 106-MtzR)
106res
106sus
713res
PC1
PC2
PC3
TMT 3 (713-MtzS vs 713-MtzR)
713res
713sus
WBres
PC1
PC2
PC3
C)
TMT 1 (WB-MtzS vs WB-MtzR)
Proteins
P-Value
TMT 2 (106-MtzS vs 106-MtzR)
Proteins
P-Value
TMT 3 (713-MtzS vs 713-MtzR)
Proteins
P-Value
