# Supplementary material for: Differential protein expression and post-translational modifications in metronidazole-resistant Giardia duodenalis
Source: Gigascience. 2018 Mar 13;7(4):giy024. doi: 10.1093/gigascience/giy024 (PMC5913674; doi:10.1093/gigascience/giy024)
Supplement: Supplemental material [file giy024_supp.zip › Emery et al, Supplementary Figure 6.pdf]

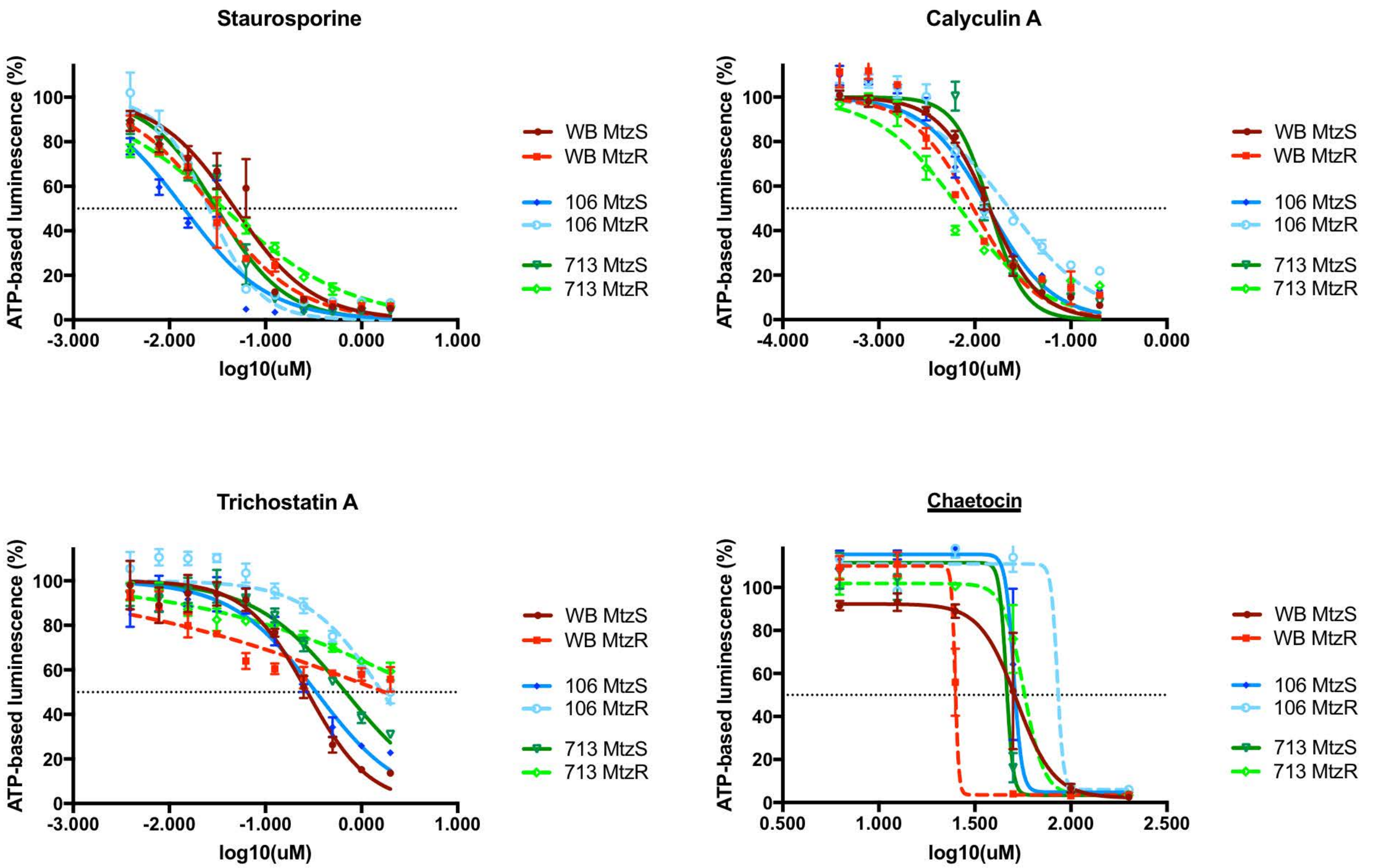

	WB MtzS	WB MtzR		106 MtzS	106 MtzR		713 MtzS	713 MtzR	
	Mtz IC_{50} (µM)	Mtz IC_{50} (µM)	RF	Mtz IC_{50} (µM)	Mtz IC_{50} (µM)	RF	Mtz IC_{50} (µM)	Mtz IC_{50} (µM)	RF
**Staurosporine**	0.05	0.03	0.6	0.01	0.03	1.8	0.03	0.04	1.2
**Calyculin A**	0.014	0.009	0.7	0.014	0.023	1.7	0.014	0.007	0.5
**Trichostatin A**	**0.27**	**1.74**	**6.4**	**0.34**	**1.64**	**4.8**	**0.68**	**5.10**	**7.5**
**Chaetocin**	52.31	~24.98	0.5	~50.37	~84.93	1.7	~46.06	56.76	1.2
