# Supplementary figures and images for: Differential protein expression and post-translational modifications in metronidazole-resistant Giardia duodenalis
Source: Gigascience. 2018 Mar 13;7(4):giy024. doi: 10.1093/gigascience/giy024 (PMC5913674; doi:10.1093/gigascience/giy024)

log2(FC) protein

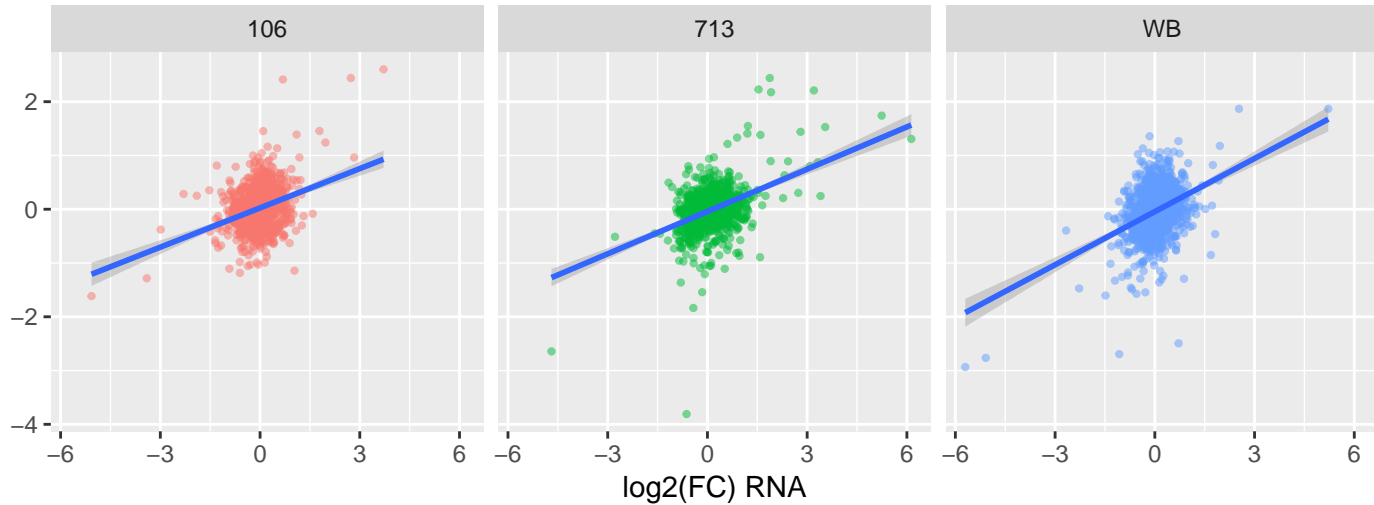

Supplement: Supplemental material [file giy024_supp.zip › Emery et al, Supplementary Figure 2.pdf]

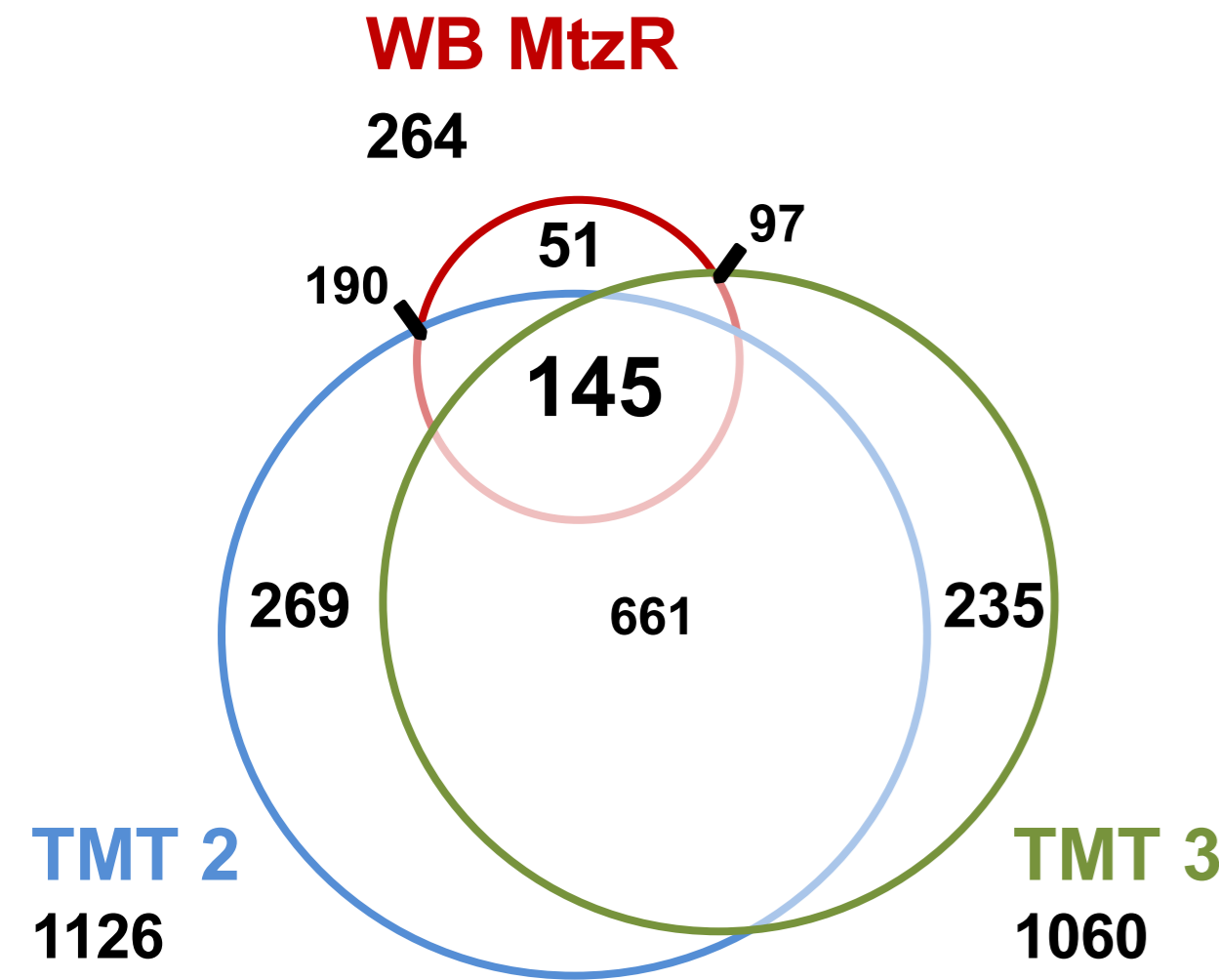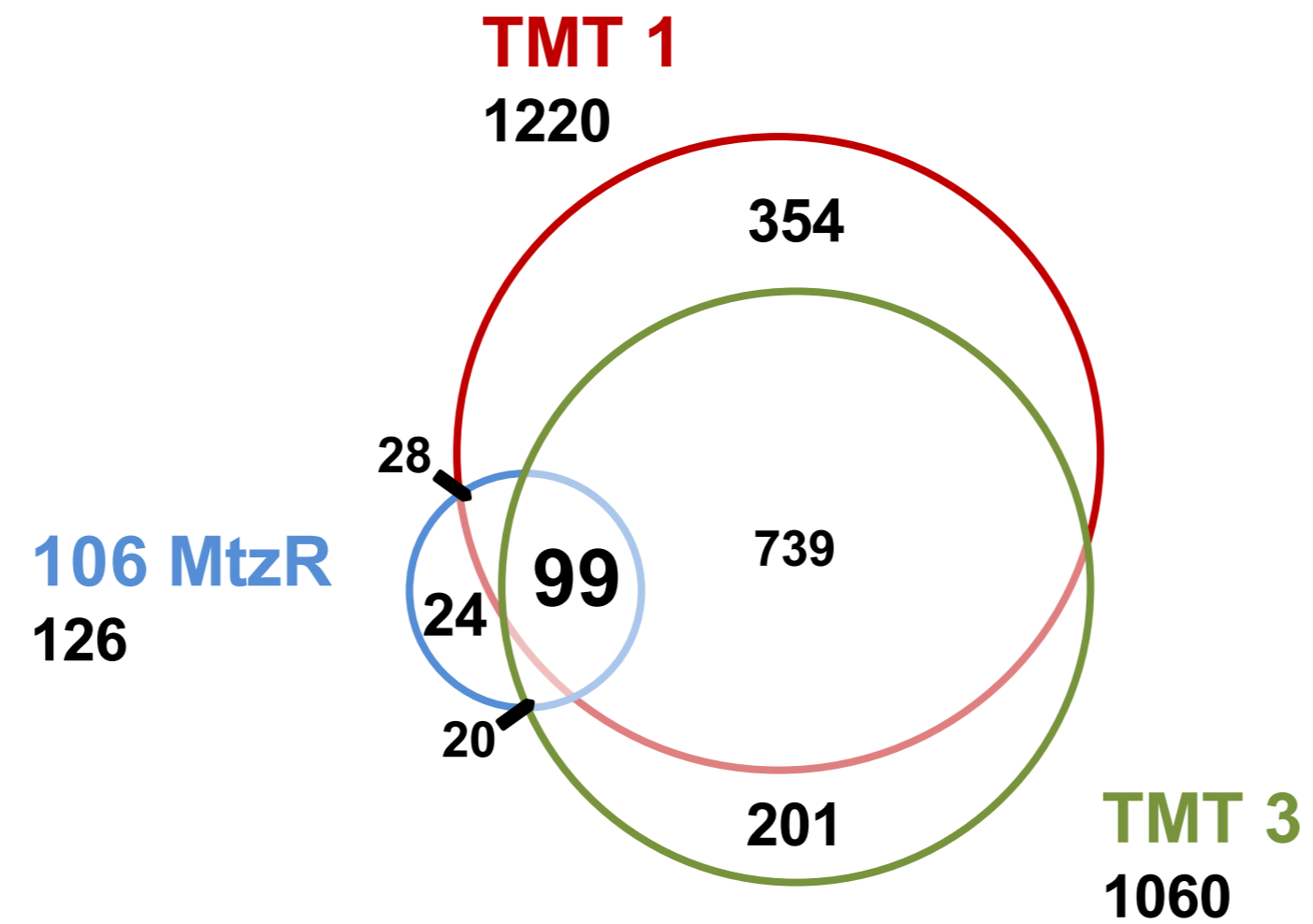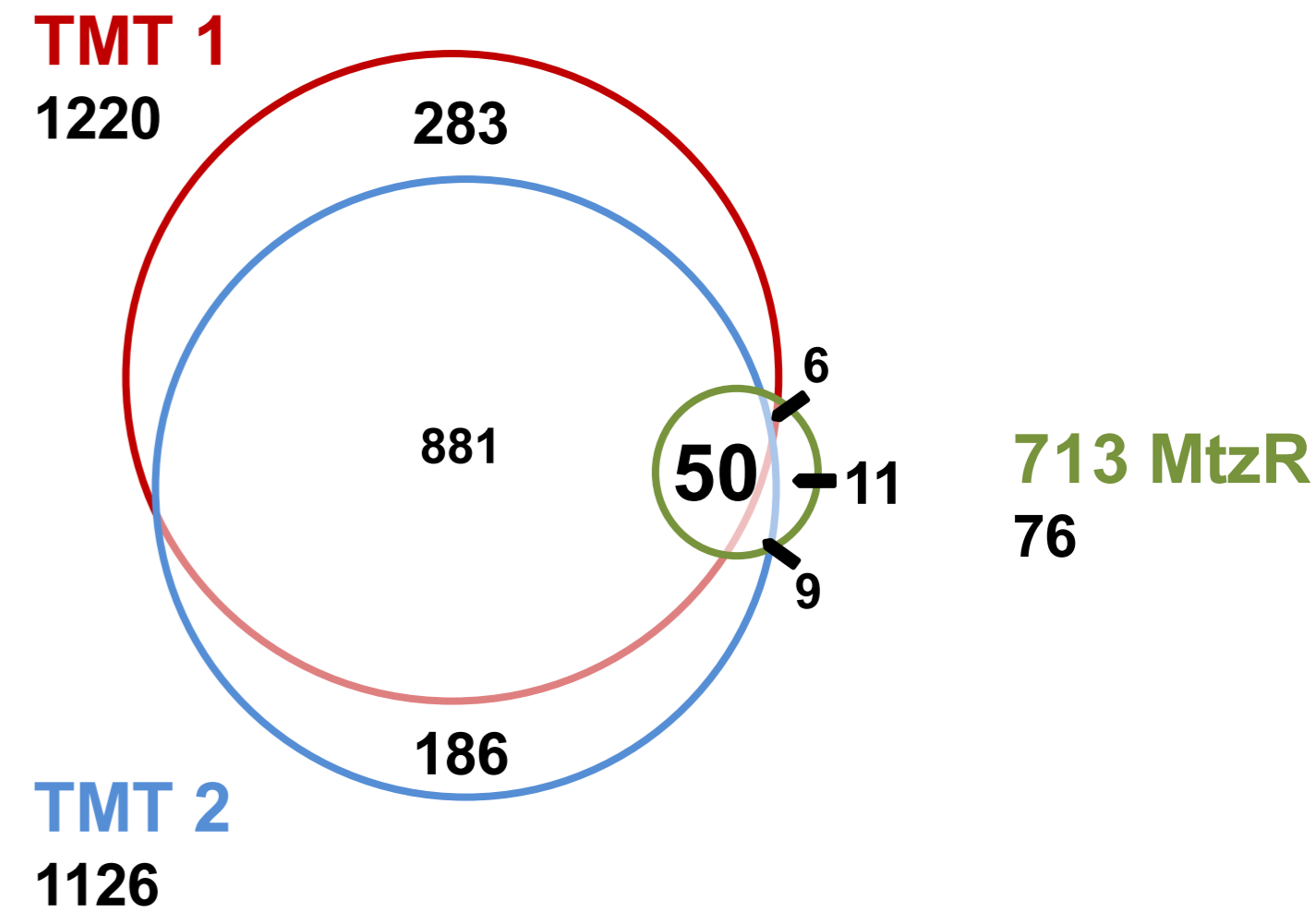

Supplement: Supplemental material [file giy024_supp.zip › Emery et al, Supplementary Figure 3.pdf]

WB MtzR

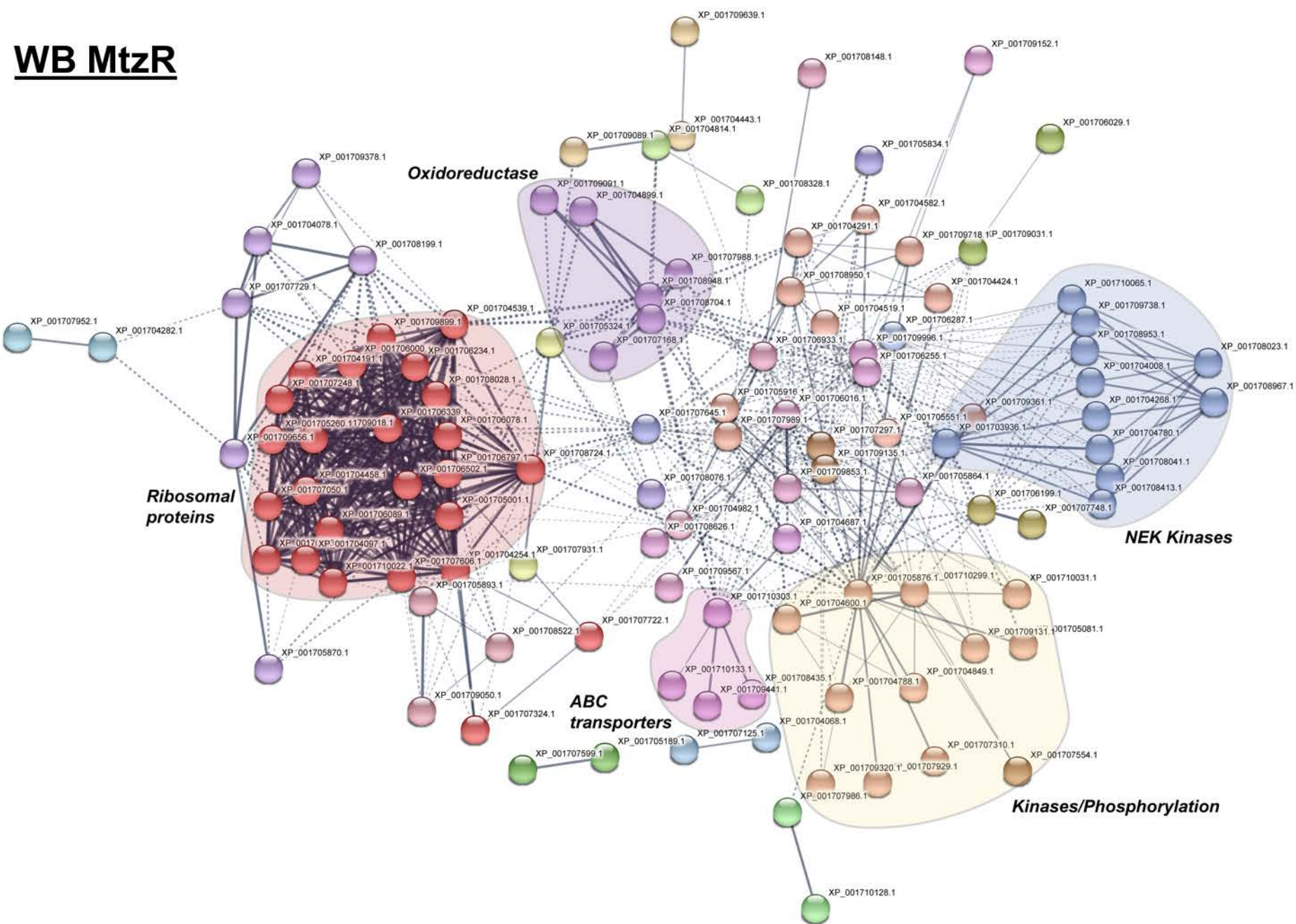

106-MtzR

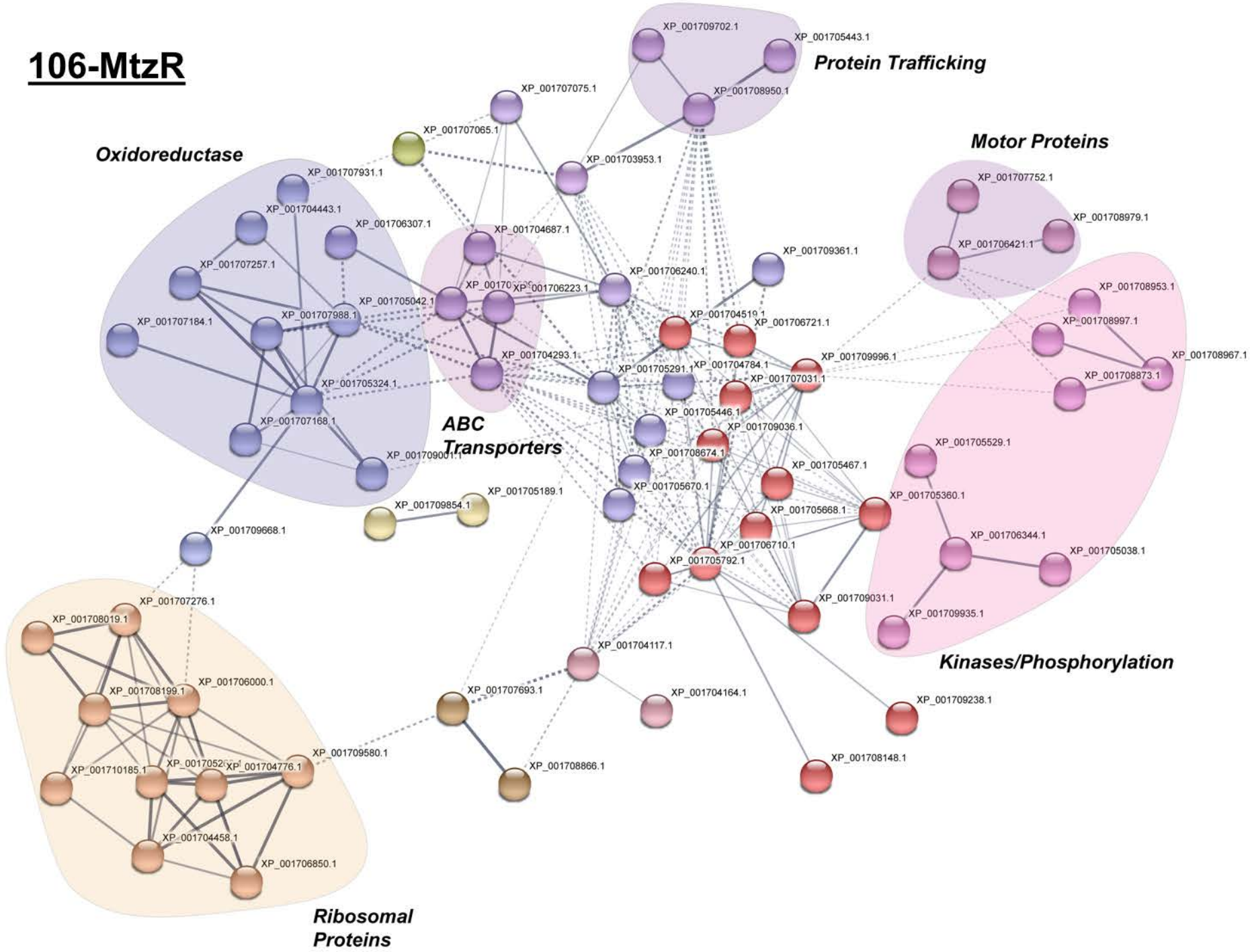

713 MtzR

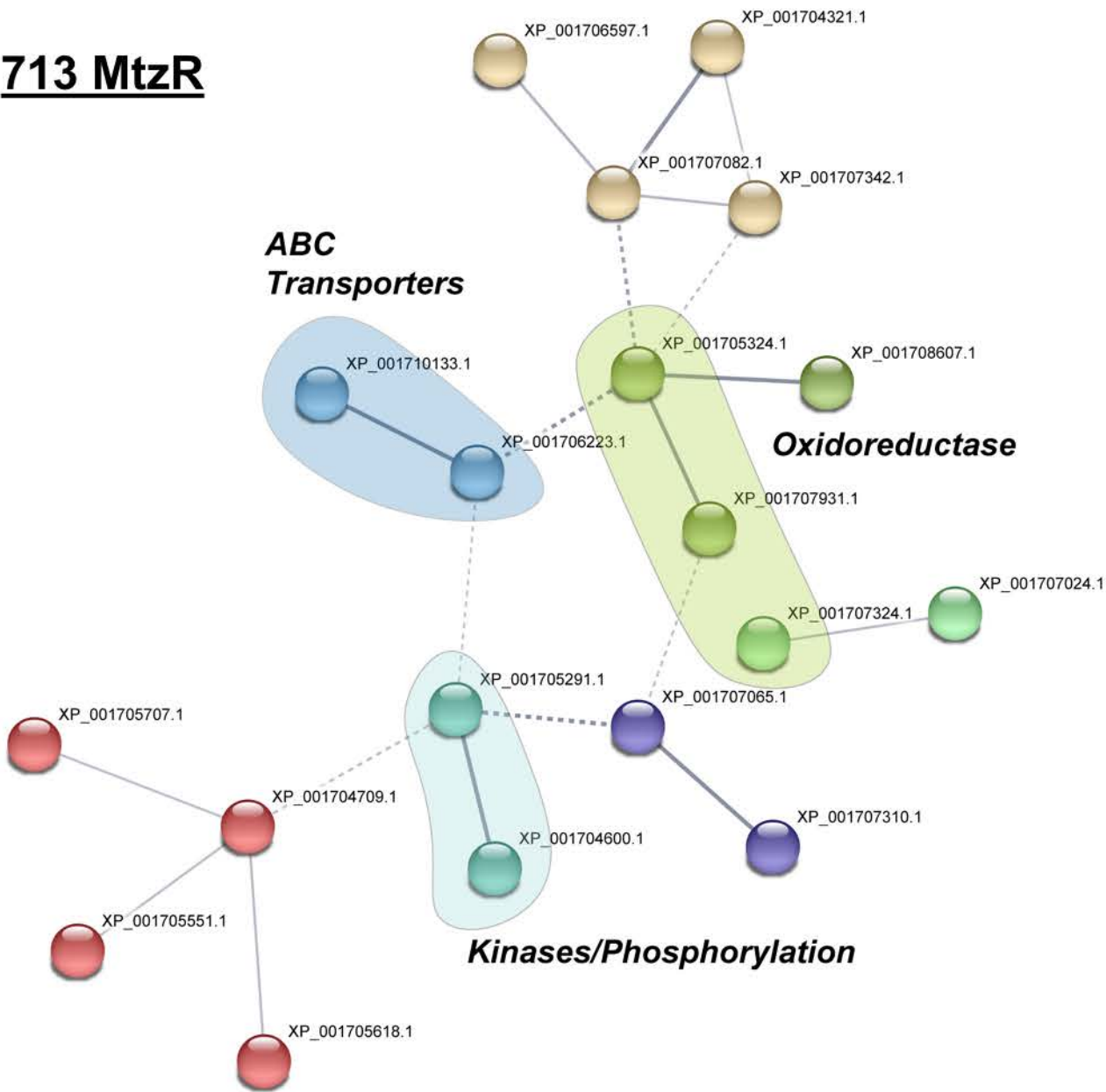

Supplement: Supplemental material [file giy024_supp.zip › Emery et al, Supplementary Figure 4.pdf]

### Anti-H3

kDa

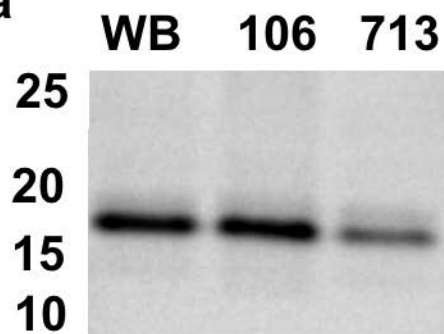

### KAc

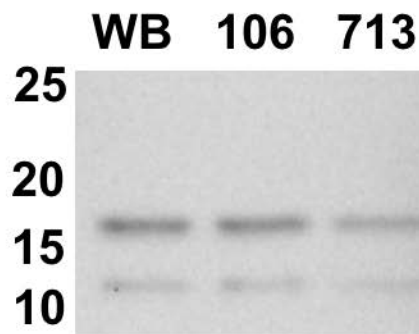

### KMme

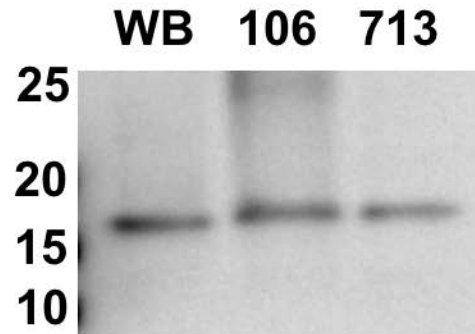

### Anti-H4

kDa

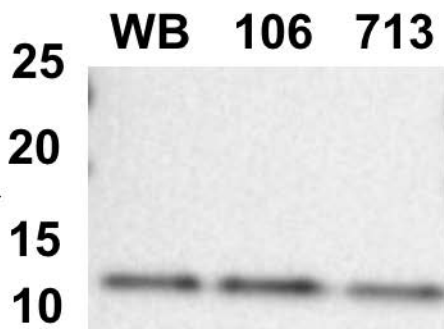

### KAc

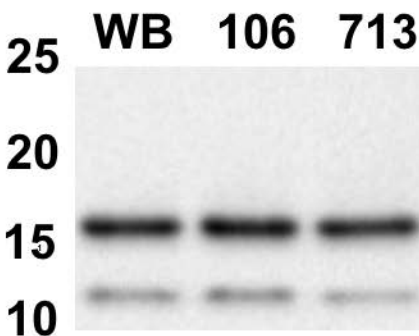

### KMme

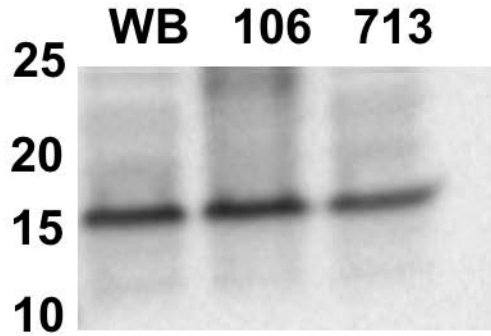

Supplement: Supplemental material [file giy024_supp.zip › Emery et al, Supplementary Figure 5.pdf]

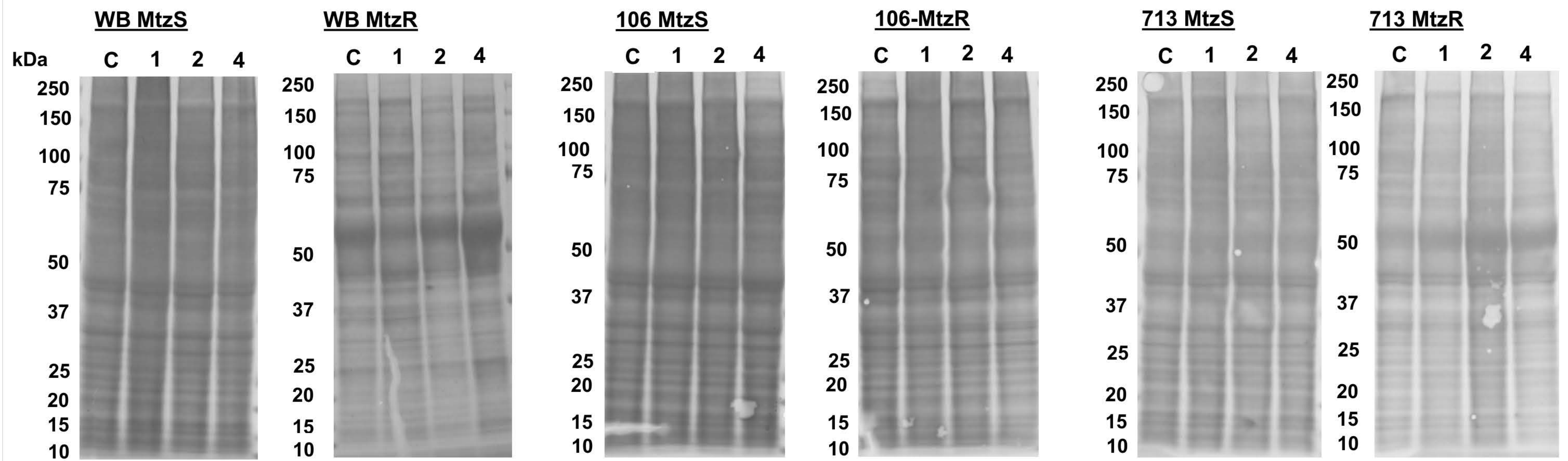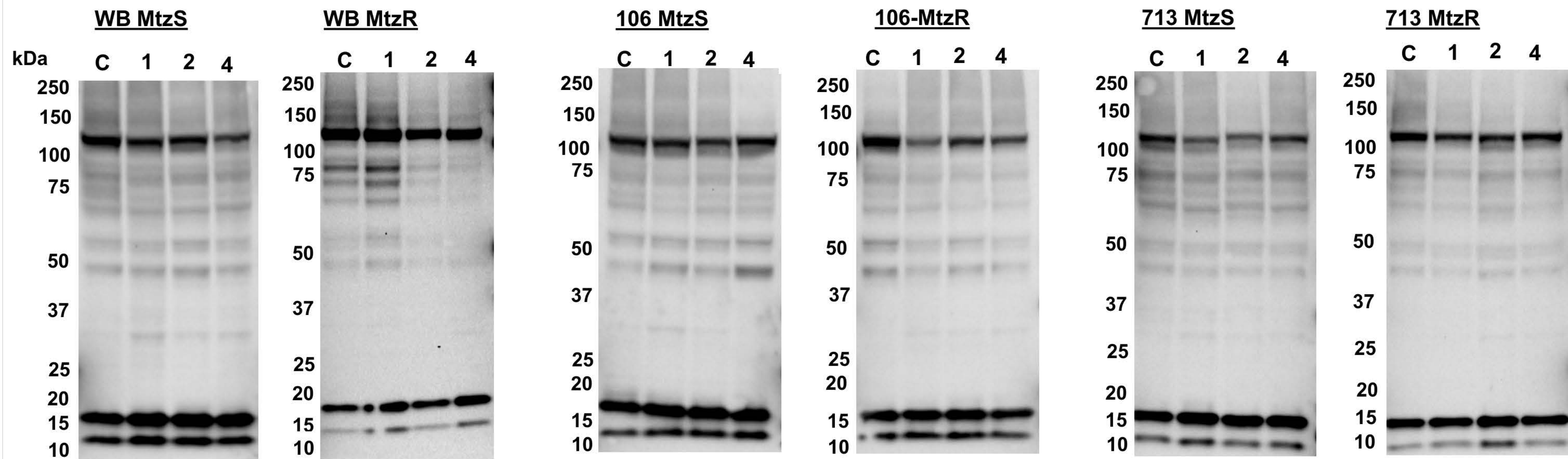

Supplement: Supplemental material [file giy024_supp.zip › Emery et al, Supplementary Figure 7.pdf]

**WB MtzS**

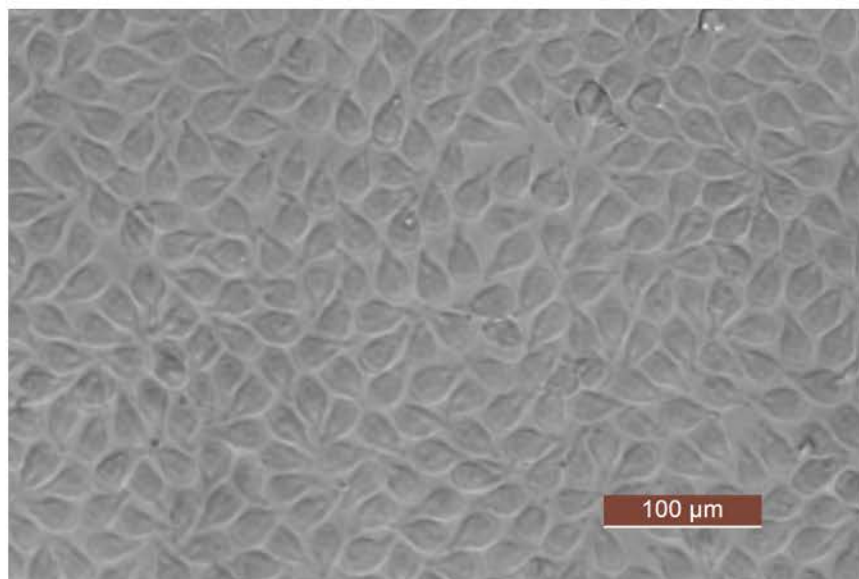

**WB MtzR**

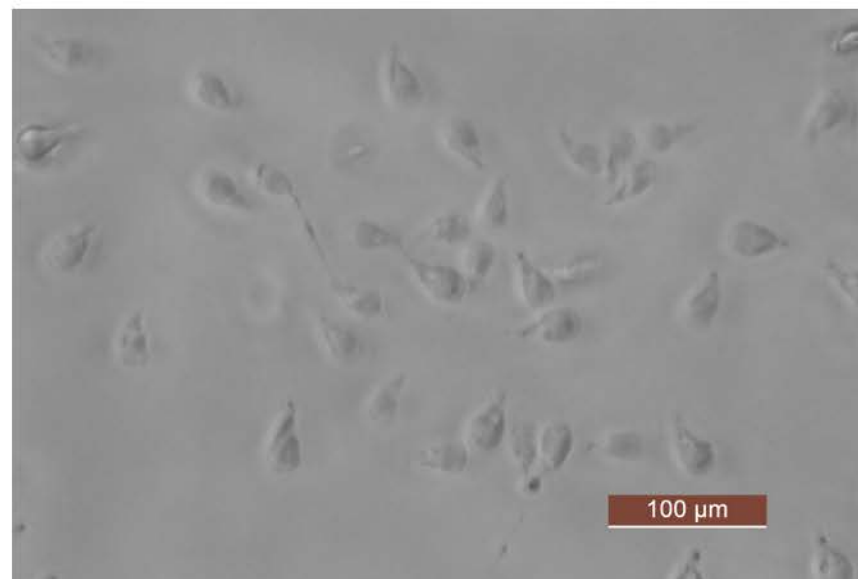

**106 MtzS**

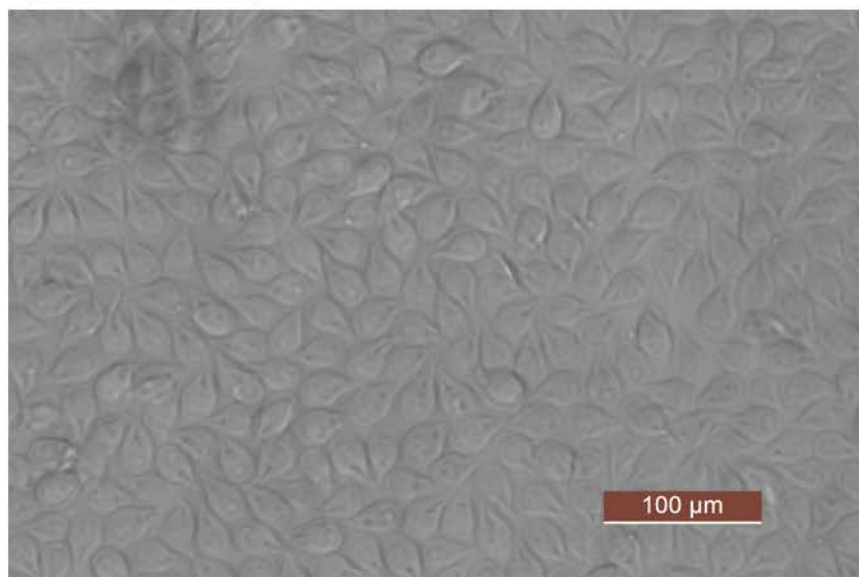

**106-MtzR**

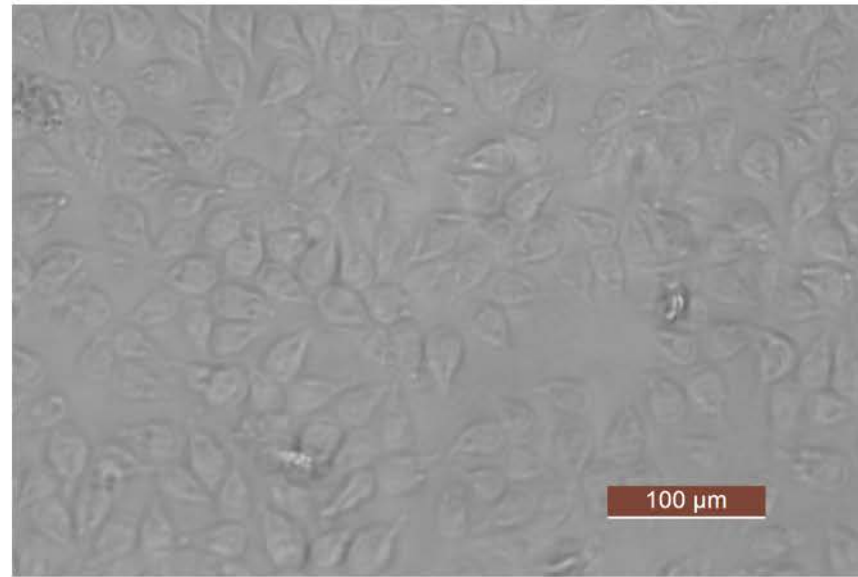

**713 MtzS**

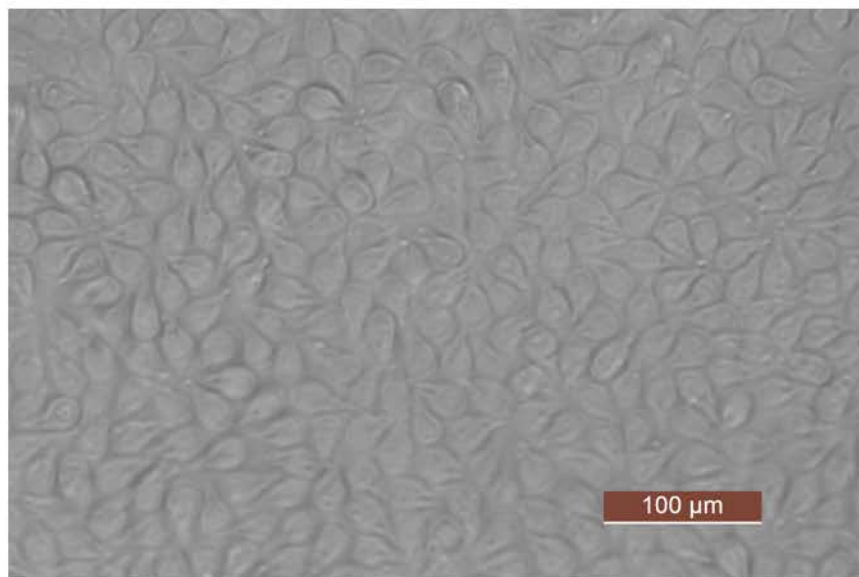

**713 MtzR**

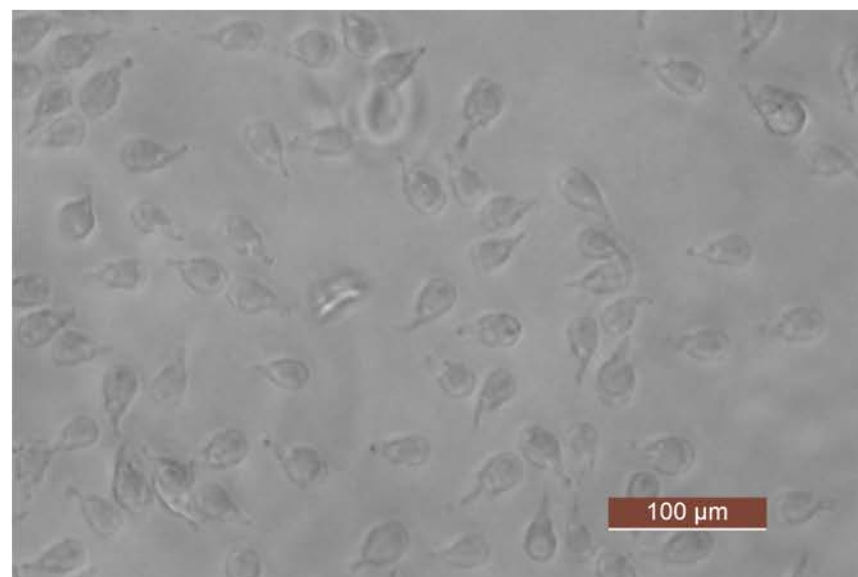

Supplement: Supplemental material [file giy024_supp.zip › Emery et al, Supplementary Figure 8.pdf]

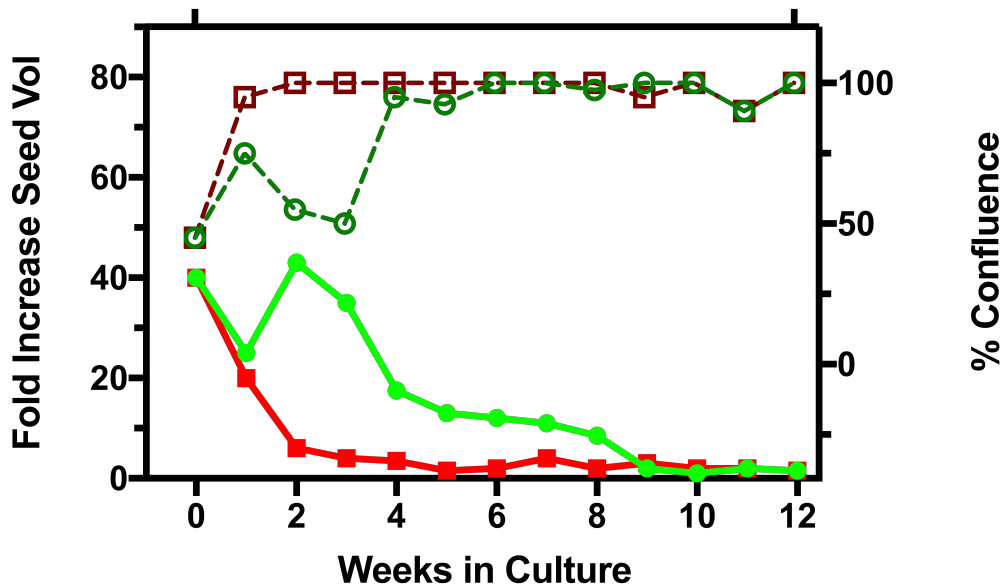

● 713 Seed Vol

○ 713 % Confluence

■ 106 Seed Vol

■ 106 % Confluence

Supplement: Supplemental material [file giy024_supp.zip › Emery et al, Supplementary Figure 9.pdf]
