# Supplementary material for: Differential protein expression and post-translational modifications in metronidazole-resistant Giardia duodenalis
Source: Gigascience. 2018 Mar 13;7(4):giy024. doi: 10.1093/gigascience/giy024 (PMC5913674; doi:10.1093/gigascience/giy024)
Supplement: Response_to_Reviewer_Comments_Original_Submission.pdf [file giy024_response_to_reviewer_comments_original_submission.pdf]

Author's Response To Reviewer Comments

Nicole Nigoy, Ph.D.
Gigascience

Dear Nicole,
We thank the reviewers for their insightful comments and suggestions regarding our manuscript. We have incorporated these comments and additional experiments where possible for us to do so, and we believe the manuscript is greatly improved. Specifically, we have added additional experiments using broad-spectrum PTM inhibitors to further characterize the role of PTMs in Mtz resistance, which strengthened our results regarding the relationship between acetylation and Mtz resistance. We have also added five new supplementary figures, including DEPs as protein-protein interaction networks, and western blots of Anti-H3 and Anti-H4 to confirm the identities of protein bands on Anti-KAc and Anti-MMe blots. Responses to specific reviewer comments can be found below.

We hope that these additional comments and experiments address our reviewer’s concerns and thank you for your further consideration.

On behalf of all co-authors

Dr Samantha Emery
Walter and Eliza Hall Institute

Specific reviewer comments

Reviewer #1:
R1-Q1: Validating PTM-related data using chemical inhibitors of protein acetylation, methylation and phosphorylation: We thank the reviewer for their suggestion, which we have attempted to address in our revision. The PTM networks of Giardia are poorly studied, and inhibitors of these networks have not been widely screened such that the targeted and specific chemical inhibition of Giardia PTM enzymes is not currently possible. We have focused our additional work on four broad inhibitors, Trichostatin A (TSA: acetylation), Chaetocin (Methylation) and Staurosporine and Calyculin A (phosphorylation), of which all but Chaetocin have known activity in Giardia.
To test our findings, we determined IC50 dose response curves in MtzS and MtzR lines of all 3 isolates, finding that each MtzR line has significant cross-resistance to Trichostatin A, a broad-spectrum deacetylase inhibitor. This further supports that lysine acetylation has strong links to Mtz resistance, as per our paper’s conclusions. We then exposed all 6 lines to 1, 2 and 4uM TSA for 8 hours for optimization, and selected 2uM TSA for a subsequent 18 hour exposure. These were immunoblotted with anti-KAc. MTZs lines showed hyperacetylaton of histones 8 hours and increased KAc of multiple other protein features following TSA exposure for 16 hours. However, MtzR lines showed decreases in KAc, which points to widespread changes in activity of KAc network enzymes in MtzR lines, not just deacetylases.

The manuscript has been amended to include these experiments:
The methods and results sections each now include the section "Chemical Inhibitors of post-translation protein modification networks". We have added an additional figure to the manuscript (Figure 6), as well as two new Supplementary Figures (Supplementary Figure S6, Supplementary Figure S7). There have also be changes to the abstracts, discussion and results to incorporate these additional experiments into the pre-existing sections of the manuscript.

R1-Q2: PTM protein band identification. We intend to identify the PTM substrates that change their modification state with Mtz exposure and resistance through IAP experiments in a subsequent study. However, as none of these networks have been investigated in Giardia, they warrant separate and dedicated analysis. IAP pulldowns of modified peptides is a significant undertaking, involving considerable costs and time, and, in our opinion, it is beyond the scope of this experiment to perform pull downs and quantitative mass spectrometry for four networks in six lines. Our primary finding with regards to PTMs is, at present, a general finding, namely that there is clear remodeling of PTM networks (demonstrated by western blot) and that there is a link between lysine acetylation and MtzR, confirmed here based on Trichostatin exposures.

Reviewer #2:
R#2-Q1: Presentation of our proteomic analysis. Our manuscript has been written to highlight that while the DEPs in each line are different, there are overarching similarities in the type of functions of these proteins (e.g., ABC transporters, oxidoreductases/electron transport proteins, membrane proteins, cell signalling), which points to overlapping mechanisms underpinning MtzR. While we respect the reviewer's opinion, in our opinion, this is the best way to present this data.

R#2-Q2 – VSPs - turnover. Although VSPs do spontaneously change during growth in Giardia, certain VSPs confer higher tolerance to bile, or have been hypothesized to increase tolerance to oxidative stress (Ma'ayeh et al, 2015, Int J Parasitol). Previous transcriptomics of Mtz resistance (Ansell et al, 2017, Front. Microbiol.; Muller et al, 2007, J Antimicrob Chemother) have reported drastic changes to VSP expression associated with resistance, including specific variants. Given that VSPs are post-transcriptionally regulated (Prucca et al, 2008, Nature) and are functionally enriched as a cluster in all three lines, our protein expression data is an important complement to transcriptomic data and as such it has been discussed. That we do not see convergence of different MtzR lines to similar variants suggests either that these VSP changes are stochastic turn-over (as per the reviewer's suggestion) or that certain variants confer greater MtzR tolerance. We feel recording this observation is important, but have modified its description in the manuscript to reflect the reviewer's concerns (please see R#2-Q4 below).

R#2-Q3 –Cloning lines for VSP turnover analysis: Given the historical precedence of these lines in Mtz resistance literature, we are not able to clone these isolates out and diverge them from observations in previously-generated datasets.

R#2-Q4 - In the discussion section the lines 1-10 of page 19 are absolutely speculative, considering that the authors do not analyze the VSP turnover in the MtzR lines. We have removed this section from the discussion, and we have amended the section of the discussion regarding VSP turnover and differential expression.

R#2-Q5 - PTM analysis is very preliminary and needs more experiments. Please see R1-Q1 response and additional experimental listed above.

R#2-Q6 - Ponceau vs an internal control, like anti-tubulin, to validate each blot. We have used Ponceau because (1) it is difficult to source verified antibodies for Giardia to use a loading control and (2) with six lines with very different proteomic profiles it is even more difficult to identify a consistent loading control with equal expression across the six lines. Tubulin, for example, would not be an appropriate choice as it is very close to the DE cutoffs in several line). Therefore, we felt Ponceau, which has been used previously for Westerns in Giardia (Sonda et al, 2010, Mol Microbiol), was a reliable alternative.

R#2-Q7 - This experiment should be complemented with IPP using antibodies and mass spectrometry. Characterising protein substrates of PTM networks, including in context to Mtz resistance is indeed the next step in our work, however we feel that these currently uncharacterized networks warrant separate, dedicated analysis. Please see R1-Q2 in regard to identification of PTM modified proteins and IAP experiments.

R#2-Q8: Observations on acetylated or methylated histones. We have now performed additional western blots using Anti-H3 and Anti-H4 side by side with Anti-KAc and Anti-MMe to show that anti-H3/H4 align with the modified bands on the corresponding blots in the wild type isolates (WB, 106 and 713). These have been added in Supplementary Figure S5 and the manuscript altered to reflect the addition of the figure as follows: “Both KAc and K-MMe modification detection for histone variants is consistent with previously detected histone modification states via Western blot in Giardia [54], and subsequent immunoblotting with Anti-H3 and Anti-H4 showed that the modified protein bands at ~17 and ~11KDa in KAc and KMMe blots aligned with antibodies for these histone variants (Supplementary Figure 5).”

R#2-Q9 – Analyzing the expression of HAT, HMT, phosphatases using qRT-PCR. Complete transcriptomic analyses have already been conducted and analysed from the same cell cultures (from total RNA co-purified from the same source cells as all proteins evaluated here for mass spec proteomics) and published earlier this year (Ansell et al, 2017, Front Microbiol). There is good agreement between RNA and protein data (Supplementary Figure S2), and as such the expression of KATs and KDACS were discussed on pg21. The RNAseq data provided in Ansell et al (2017), was internally controlled at publication by qRT-PCR and does not need to be repeated here as we are working from the same cell pellets. We have revised the manuscript to make this clear.

R#2-Q10 – Eliminate discussion section lane 1-22 of page 21. More experiments related with the analysis of the expression of KAT and K/HDAC should be performed. Given we have reasonable correlation between Protein-RNA log2 fold change in the two experiments for genes identified in both datasets (Supplementary Figure S2), we believe it is valid to intersect the protein dataset with the transcript data from these lines. Lines 1-22 reflect a robust discussion of transcript and protein results of KAc network enzymes, not just KAT expression in the transcript data, and we believe it is important to keep this discussion of the wider KAc network in the manuscript.

R#2-Q11 – Why is the PTM pattern at P0 is different from the one in MtzR lines (figure5) if they were growth in the presence of Mtz? There were not changes, but due to increases in the intensity of the histone variant bands (H3 and H4 at ~17 and ~11kDa) and the prominent band at ~30kDa in MMe profiles we had to lower chemiluminescence exposure time to prevent oversaturation of the major bands thus allowing comparisons of profiles between passage timepoints. This resulted in the loss of some low intensity bands. This information on exposure times has been added to the Figure legend for Figure 7 (formerly figure 6) and the methods.

R#2-Q12 - Results found using WB are speculative, and in some cases, like in figure 6 i and ii, there is an increase in the general K-MMe pattern at P8 in 106 that then decrease. Again, it is necessary the use of an anti-tubulin antibody. It would be interesting to include the WB at P24. The increase at P8 and decrease at P16 fits the alternative profile of loss of Mtz resistance at P8 and then gain at P16 in resistance, which is different to the resistance profile and WB profile in 716 which has decreases in resistance at P8 and P16.

R#2-Q13 - All the results related to histone modifications should be avoided. Histone modifications have been addressed above (see R#2-Q6) with the additional Anti-H3 and Anti-H4 Western blots in Supplementary Figure S5.

R#2-Q14 - The conclusions in page 13 line 58 and page 14 (lines 1-4) are speculative. The statement the reviewer is referring to proposed as a hypothesis preceded by the statement "it is possible…". We believe that the observation of lower abundance of ventral disc proteins and lower adherence in these lines is interesting (further reinforced in the new supplementary figure S8, which shows reduced adherence in WB and 713 MtzR lines). We have adjusted our revised manuscript to make more clearly that we are proposing this as a potential hypothesis, not a conclusion. We agree that our data can't test this hypothesis at present.

R#2-Q15 - Sir2 10708 is a hypothetical protein that should be named: putative Sir2. While the 10708 is a hypothetical protein in GiardiaDB, its sequence homology and function was investigated as a Sir2 gene late last year (Carranza et al, 2016 Int J Biochem Cell Biol) and our nomenclature reflects this.

Reviewer 3:
R#3-Q1: Analyze the interactome (the differential protein-protein interaction networks) using DEPs. In order to analyses protein-protein interactions, DEPs from the three lines were submitted to STRING (Search Tool for the Retrieval of Interacting Genes) software (v10.5) (http://string.db.org) . The results of this have been added as Supplementary Figure 4 and networks interactions largely complement the functional enrichment analyses as investigated using DAVID in the manuscript. The methods have been amended to include the reference to STRING, and the supplementary figure has been integrated into the results of the text.

R#3-Q2 – qPCRs to assess comparisons (figure FS2) of protein expression and RNA transcript levels of the respective genes from the same strains (Ansell et al., 2017). Please see R#2-Q7 for our response regarding qPCR-based testing of our RNA-seq data. For our proteomic datasets, we have followed statistical techniques previously employed for TMT proteomics in Giardia (Emery

et al, 2016, Sci Reps) and recognized in the broader discipline of isobaric labelling (Mahoney et al, 2011; Pascovici et al, Proteomics, 2016). We have used robust statistical cutoffs to analyse these data, including 1) setting both fold change and p-value cutoffs for differential expression, 2) using unsupervised multivariate Principal Component Analysis (PCA) and 3) analysing the p-value distribution using the paired t-tests between triplicates of MtzS and MtzR fold changes. All of these are represented in Supplementary Figure 1. We and others have shown (Pascovici et al, Proteomics, 2016) that this approach is more reliable than targeting a small subset of proteins by western blot.

R#3-Q3 – Identify acetylated, methylated or phosphorylated substrates in MtzR lines using specific antibody pulldowns. This is indeed the next step in the work, however, our primary finding in relation to PTMs, is that lysine acetylation is closely associated with MtzR phenotype. This hypothesis is further supported by inhibitor studies provided in our revised submission (please see R#1-Q1). Regarding direct IAP pulldowns, please see our response to R#1-27.

R#3/-Q4 - Explain the isolate variation in Mtz tolerance in the absence of drug. We believe the author is referring to the different IC50 profiles of 713 and 106 through P8, P16 and P24 after cessation of drug selection. In regard to this, we have provided some hypotheses as to why these isolates tolerate Mtz differently after drug selection, in particular the possibility of stable resistance traits in unresolved chromosomal aberrations and/or nonsense mutations in key Mtz resistance genes (on pg 22 of the manuscript discussion). However this is a novel and unexpected result, and will require more thorough and extensive investigation in follow-up experiments beyond the scope of this study.
