# Supplementary material for: Differential protein expression and post-translational modifications in metronidazole-resistant Giardia duodenalis
Source: Gigascience. 2018 Mar 13;7(4):giy024. doi: 10.1093/gigascience/giy024 (PMC5913674; doi:10.1093/gigascience/giy024)
Supplement: Reviewer_1_Report_(Original_Submission) -- Carmen Faso — 05 Sep 2017 Reviewed [file giy024_reviewer_1_report_(original_submission).pdf]

**Reviewer Report**

**Title:** Differential protein expression and post-translational modifications in Metronidazole-resistant Giardia duodenalis

**Version:** Original Submission **Date:** 05 Sep 2017

**Reviewer name:** Carmen Faso

**Reviewer Comments to Author:**

The authors present the first systematic quantitative proteomic study of metronidazole (MTZ) resistance in Giardia duodenalis using quantititative proteomics on 3 cohorts, each consisting of a MTZ-resistant line and its MTZ-sensitive isogenic partner. The decision to use isogenic pairs was a very sound one in that the authors wished to minimize any variability between lines that was not MTZ-resistance related.

The experimental design is sound and includes all controls necessary to address the question of what changes in the proteome of resistant vs. sensitive cell lines. Interestingly and not entirely surprisingly, these changes do not seem to affect a specific protein family rather, MTZ resistance is associated to pleiotropic effects on the proteome. Given the mode of action of MTZ, the involvement of oxidative pathways was to be expected. Post-translational modifications seem to play a role in resistance and its loss, with clear changes in PTM profiles of acetylated, methylated and phosphorylated proteins. The relevance of these data go beyond the Giardia research community, carrying implications for treatment of other parasitic protists.

The work presented is valuable. The manuscript is mostly descriptive and at times reads as a list of protein families. This is often the case when reporting on data generated using shot-gun proteomics as the authors have done. To include more functional data, my suggestion to the authors is to try to validate their PTM-related data using chemical inhibitors of protein acetylation, methylation and phosphorylation. These chemicals are readily available and could be used to test whether treatment mimics MTZ resistance or loss thereof by analysing and comparing total protein protein profiles, as the authors have already done for figure 6. Furthermore, it would be interesting to know whether the authors have specifically identified any of the protein bands which are differentially displayed following PTM detection. I would like to see the results of both experiments i.e. chemical treatment and protein band identification.

Otherwise, the manuscript is well-written, thresholds for significance were well-chosen and the authors strove to intersect their data with previously-generated datasets. The text presents only minor mistakes that can be easily edited. The data is available on the PRIDE database, covers all their experiments and should be made public as soon as possible.

**Methods**

Are the methods appropriate to the aims of the study, are they well described, and are necessary controls included? Yes

**Conclusions**

Are the conclusions adequately supported by the data shown? Yes

**Reporting Standards**

Does the manuscript adhere to the journal's guidelines on [minimum standards of reporting?]() Yes

**Statistics**

Are you able to assess all statistics in the manuscript, including the appropriateness of statistical tests used? Yes, and I have assessed the statistics in my report.

**Quality of Written English**

Please indicate the quality of language in the manuscript: Needs some language corrections before being published

**Declaration of Competing Interests**

Please complete a declaration of competing interests, considering the following questions:

- Have you in the past five years received reimbursements, fees, funding, or salary from an organisation that may in any way gain or lose financially from the publication of this manuscript, either now or in the future?
- Do you hold any stocks or shares in an organisation that may in any way gain or lose financially from the publication of this manuscript, either now or in the future?
- Do you hold or are you currently applying for any patents relating to the content of the manuscript?
- Have you received reimbursements, fees, funding, or salary from an organization that holds or has applied for patents relating to the content of the manuscript?
- Do you have any other financial competing interests?
- Do you have any non-financial competing interests in relation to this paper?

If you can answer no to all of the above, write 'I declare that I have no competing interests' below. If your reply is yes to any, please give details below.

I declare that I have no competing interests

I agree to the open peer review policy of the journal. I understand that my name will be included on my report to the authors and, if the manuscript is accepted for publication, my named report including any attachments I upload will be posted on the website along with the authors' responses. I agree for my report to be made available under an Open Access Creative Commons CC-BY license (http://creativecommons.org/licenses/by/4.0/). I understand that any comments which I do not wish to be included in my named report can be included as confidential comments to the editors, which will not be published.

I agree to the open peer review policy of the journal

To further support our reviewers, we have joined with Publons, where you can gain additional credit to further highlight your hard work (see: https://publons.com/journal/530/gigascience). On publication of this paper, your review will be automatically added to Publons, you can then choose whether or not to claim your Publons credit. I understand this statement.

Yes
