# Supplementary material for: Differential protein expression and post-translational modifications in metronidazole-resistant Giardia duodenalis
Source: Gigascience. 2018 Mar 13;7(4):giy024. doi: 10.1093/gigascience/giy024 (PMC5913674; doi:10.1093/gigascience/giy024)
Supplement: Reviewer_2_Report_(Original_Submission) -- Andrea Rópolo — 14 Sep 2017 Reviewed [file giy024_reviewer_2_report_(original_submission).pdf]

Reviewer Report

Title: Differential protein expression and post-translational modifications in Metronidazole-resistant Giardia duodenalis

Version: Original Submission **Date:** 14 Sep 2017

Reviewer name: Andrea Rópolo

Reviewer Comments to Author:

In this manuscript the authors analyze in susceptible and resistant Mtz lines the protein expression using quantitative proteomics in the protozoan parasite Giardia duodenalis. The authors found differentially expressed proteins in the three resistant lines compared to their isogenic Mtz susceptible lines, and analyze some groups of proteins and enzymes (EGF-like proteins, VSP, oxidoreductase enzymes, PFOR, pyruvate catabolism, kinases, among others). Also, they analyze using WB the acetylation/methylation/ubiquitination/phosphorylation of proteins in susceptible and resistant lines. Finally, they analize the phenotypic plasticity after cessation of drug selection. Although the data obtained from the proteomic analysis is extensively described, the results are very speculative and the data presented here is only preliminary.

Major points: The analysis of the data emerging from the proteomic analysis is complete, however, in the way it is written it is very boring and the reader can not really size the importance of the data, especially because in each issue the results are mixed between the three resistant lines. I suggest to re-write each paragraph analyzing each MtzR line in particular, and then in the discussion it is possible to refer to the similarities and differences between the three lines, considering that although all of them are Mtz resistant, the IC50 are different between them (particularly between 713 and the other ones), and also the proteomic profile is different.
With respect to the analysis performed with VSPs, it is not significant because VSPs vary spontaneously during growth of Giardia in culture. Therefore, I suggest that if the authors want to compare VSP in the three different cell lines they must start the experiment from a clone expressing only one VSP, and do the proteomic analysis at different growth times in each line. In this way they could analyze how the VSP pattern changes in each strain and then compare the MtzR lines. Alternatively, they should repeat the experiment over time, because the expression of each VSP may vary among culture time. There they will likely find different results after P24. However, I consider that the significance of these results is relative, considering that the three strains are different from each other.
In the discussion section the lines 1-10 of page 19 are absolutely speculative, considering that the authors do not analyze the VSP turnover in the MtzR lines.
The analysis of the PTM is very preliminary and need more experiments to infer the involvement of PTM importance in MtzR lines. Although the authors used ponceau to verify consistent protein loading, the use of an internal control, like anti-tubulin, should be added to each blot. On the other hand, this experiment should be complemented with IPP using the antibodies and then mass spectrometry of the putative band differentially expressed to conclude something about PTM in the three MtzR lines. In the

way it is analyzed the data is very speculative. Especially the authors make conclusions related to acetylated or methylated histones only considering the molecular weight of the bands observed. But, to analyze PTM of histones it is necessary to isolate histones (they refer to the reference 54, where the western blot was performed in isolated histones). Considering that histones are not the only one proteins in the range from 20 to 10 kDa, all the conclusions made related to histones should be eliminated from the manuscript.
Considering that the authors found differences in the acetylation/methylation/phosphorylation patterns it would be interesting to analyze the expression of HAT, HMT, phosphatases at least using qRT-PCR. I wonder if they did not found differences in the proteomic analysis related to these enzymes, as was previously found in bacterial-like Sir2 (16569).
In the discussion section lane 1-22 of page 21 should be eliminated considering that the authors were unable to analyze KATs considering that were below the detection thresholds of the assay. More experiments related with the analysis of the expression of KAT and K/HDAC should be performed.
When the authors compare the PTM profile before and after Mtz discontinuation, why the pattern in P0 is different from the one in MtzR lines (figure5) if they were growth in the presence of Mtz? Again, the results found using WB are speculative, and in some cases, like in figure 6 i and ii, there is an increase in the general K-MMe pattern at P8 in 106 that then decrease. Again, it is necessary the use of an anti-tubulin antibody. It would be interesting to include the WB at P24. All the results related to histone modifications should be avoid.
The conclusions in page 13 line 58 and page 14 (lines 1-4) are speculative considering that growth of Giardia trophozoites is not only related with its capacity to adhere, but also to reduced endocytosis, cytokinesis, etc.
Figure 3A: Sir2 10708 is an hypothetical protein in the data base of Giardia lamblia. Therefore it should be named: putative Sir2.

Methods

Are the methods appropriate to the aims of the study, are they well described, and are necessary controls included? No

Conclusions

Are the conclusions adequately supported by the data shown? No

Reporting Standards

Does the manuscript adhere to the journal's guidelines on minimum standards of reporting? Yes

Statistics

Are you able to assess all statistics in the manuscript, including the appropriateness of statistical tests used? There are no statistics in the manuscript.

Quality of Written English

Please indicate the quality of language in the manuscript: Acceptable

Declaration of Competing Interests

Please complete a declaration of competing interests, considering the following questions:

- Have you in the past five years received reimbursements, fees, funding, or salary from an organisation that may in any way gain or lose financially from the publication of this manuscript, either now or in the future?
- Do you hold any stocks or shares in an organisation that may in any way gain or lose financially from the publication of this manuscript, either now or in the future?
- Do you hold or are you currently applying for any patents relating to the content of the manuscript?
- Have you received reimbursements, fees, funding, or salary from an organization that holds or has applied for patents relating to the content of the manuscript?
- Do you have any other financial competing interests?
- Do you have any non-financial competing interests in relation to this paper?

If you can answer no to all of the above, write 'I declare that I have no competing interests' below. If your reply is yes to any, please give details below.

I declare that I have no competing interests below

I agree to the open peer review policy of the journal. I understand that my name will be included on my report to the authors and, if the manuscript is accepted for publication, my named report including any attachments I upload will be posted on the website along with the authors' responses. I agree for my report to be made available under an Open Access Creative Commons CC-BY license (http://creativecommons.org/licenses/by/4.0/). I understand that any comments which I do not wish to be included in my named report can be included as confidential comments to the editors, which will not be published.

I agree to the open peer review policy of the journal

To further support our reviewers, we have joined with Publons, where you can gain additional credit to further highlight your hard work (see: https://publons.com/journal/530/gigascience). On publication of this paper, your review will be automatically added to Publons, you can then choose whether or not to claim your Publons credit. I understand this statement.

Yes
