# Supplementary material for: Differential protein expression and post-translational modifications in metronidazole-resistant Giardia duodenalis
Source: Gigascience. 2018 Mar 13;7(4):giy024. doi: 10.1093/gigascience/giy024 (PMC5913674; doi:10.1093/gigascience/giy024)
Supplement: Reviewer_3_Report_(Original_Submission) -- Guadalupe Ortega-Pierres — 02 Oct 2017 Reviewed [file giy024_reviewer_3_report_(original_submission).pdf]

Reviewer Report

Title: Differential protein expression and post-translational modifications in Metronidazole-resistant Giardia duodenalis

Version: Original Submission **Date:** 02 Oct 2017

Reviewer name: Guadalupe Ortega-Pierres

Reviewer Comments to Author:

Review of the Ms entitled" Differential protein expression and post-translational modifications in Metronidazole resistant Giardia duodenalis" by Samantha J. Emery, Louise Baker, Brendan R.E. Ansell, Mehdi Mirzaei, Paul A. Haynes, Malcom J. McConville, Staffan G. Svärd, Aaron R. Jex
In this work the authors performed quantitative proteomics to describe differentially expressed proteins (DEPs) in three seminal Mtz resistant (MtzR) lines compared to their isogenic, Mtz susceptible, parental line. They also analyzed by Western blot, changes in post-translational modifications (PTMs) including protein acetylation, methylation, ubiquitination and phosphorylation. They quantified over 1000 proteins in each genotype and recorded substantial genotypic variation in DEPs between isotypes. The data obtained showed substantial changes in the antioxidant network, glycolysis and electron transport, and indicates potential links between pyruvate catabolism, acetyl-CoA availability and acetylation marks. Then similar analyses were carried out with lines after cessation of drug selection over 12 weeks and changes in protein profiles were detected which were related to the phenotype of the isolate.
The work included in the manuscript contains comprehensive, interesting and important information on protein profiles among the MTZ sensitive and Mtz resistant lines analyzed. The quantitative proteomics carried out by TMT isobaric labeling is well done and well analyzed. Since they already had obtained all these data, it will be important to analyze the interactome (the differential protein-protein interaction networks). To approach this, the authors can select some of the DEPs included in Table 3 and Supplementary Figure S3 (Functional Enrichments DEPs) to perform this analysis.
In figure (FS2) they compare the expression levels of many proteins detected in this work (Mtz resistance) with the transcript levels of the respective genes from the same strains (Ansell et al., 2017) however, it would be necessary to perform qPCR assays to validate their data for proteins in which substantial changes were observed.
Based on the data reported, it is important to identify all the proteins of the bands recognized by antibodies against acetylated lysine (KAc), mono-methylated lysine (KMMe), ubiquitin (Ubi), phosphorylated tyrosine (pY) (Fig. 5), and with antibodies against acetylated lysine (KAc), mono-methylated lysine (K-Mme) and phosphorylated tyrosine (pY) (Fig. 6). In each band they may identify several proteins, but it is necessary to define which one is modified (acetylated, phosphorylated, ubiquitinated)
It will be also important to identify by MS the specific residues of the PTMs of some acetylated, methylated, phosphorylated (Y), ubiquitinated proteins. Once they identify the specific residues in the proteins modified by PTMs, the data obtained could be related / integrated with those obtained with quantitative proteomics, in which some proteins were up- or down-regulated in resistant strains. This in

turn will allow to predict signaling pathways in which they could participate and the functions they could be regulating.
It would be important if the authors can provide further support to explain the isolate variation in Mtz tolerance in the absence of drug.

Methods

Are the methods appropriate to the aims of the study, are they well described, and are necessary controls included? Yes

Conclusions

Are the conclusions adequately supported by the data shown? Yes

Reporting Standards

Does the manuscript adhere to the journal's guidelines on [minimum standards of reporting?] Yes

Statistics

Are you able to assess all statistics in the manuscript, including the appropriateness of statistical tests used? No, I do not feel adequately qualified to assess the statistics.

Quality of Written English

Please indicate the quality of language in the manuscript: Acceptable

Declaration of Competing Interests

Please complete a declaration of competing interests, considering the following questions:

- Have you in the past five years received reimbursements, fees, funding, or salary from an organisation that may in any way gain or lose financially from the publication of this manuscript, either now or in the future?
- Do you hold any stocks or shares in an organisation that may in any way gain or lose financially from the publication of this manuscript, either now or in the future?
- Do you hold or are you currently applying for any patents relating to the content of the manuscript?
- Have you received reimbursements, fees, funding, or salary from an organization that holds or has applied for patents relating to the content of the manuscript?
- Do you have any other financial competing interests?

- Do you have any non-financial competing interests in relation to this paper?

If you can answer no to all of the above, write 'I declare that I have no competing interests' below. If your reply is yes to any, please give details below.

'I declare that I have no competing interests'

I agree to the open peer review policy of the journal. I understand that my name will be included on my report to the authors and, if the manuscript is accepted for publication, my named report including any attachments I upload will be posted on the website along with the authors' responses. I agree for my report to be made available under an Open Access Creative Commons CC-BY license (http://creativecommons.org/licenses/by/4.0/). I understand that any comments which I do not wish to be included in my named report can be included as confidential comments to the editors, which will not be published.

I agree to the open peer review policy of the journal

To further support our reviewers, we have joined with Publons, where you can gain additional credit to further highlight your hard work (see: https://publons.com/journal/530/gigascience). On publication of this paper, your review will be automatically added to Publons, you can then choose whether or not to claim your Publons credit. I understand this statement.

Yes
