# Supplementary material for: Differential protein expression and post-translational modifications in metronidazole-resistant Giardia duodenalis
Source: Gigascience. 2018 Mar 13;7(4):giy024. doi: 10.1093/gigascience/giy024 (PMC5913674; doi:10.1093/gigascience/giy024)
Supplement: Reviewer_3_Report_(Revision_1) -- Guadalupe Ortega-Pierres — 06 Feb 2018 Reviewed [file giy024_reviewer_3_report_(revision_1).pdf]

**Reviewer Report**

**Title:** Differential protein expression and post-translational modifications in Metronidazole-resistant Giardia duodenalis

**Version:** Revision 1 **Date:** 06 Feb 2018

**Reviewer name:** Guadalupe Ortega-Pierres

**Reviewer Comments to Author:**

Comments to the revised Ms entitled Differential protein expression and post-translational modifications in Metronidazole resistant Giardia duodenalis by Samantha J. Emery, Louise Baker, Brendan R.E. Ansell, Mehdi Mirzaei, Paul A. Haynes, Malcom J. McConville, Staffan G. Svärd, Aaron R. Jex.

The authors have addressed basically all the comments made by the reviewers and they have certainly improved the content of the manuscript. In particular, the validation of the PTM-related data using chemical inhibitors of protein acetylation, methylation and phosphorylation, as was the case on the use of Trichostatin, provided a strong support to the observation that there is a link between lysine acetylation and MtzR. Further, the analysis of the interactome (the differential protein-protein interaction networks) using DEPs from the three lines using STRING (Search Tool for the Retrieval of Interacting Genes) software (v10.5) (http://string.db.org) provided important data on the protein - protein interactions in their study. Interestingly, and as the authors stated the data included in the revised manuscript now confirms that MtzR is polygenic, plastic and likely post-translationally regulated and supports the proposed hypothesis on links between MtzR and epigenetic regulation, and highlights that changes in post-translational modifications in MtzR are widespread. I consider that the data included in the revised manuscript provide a platform for future studies on MtzR in this important parasite.

**Methods**

Are the methods appropriate to the aims of the study, are they well described, and are necessary controls included? Yes

**Conclusions**

Are the conclusions adequately supported by the data shown? Yes

**Reporting Standards**

Does the manuscript adhere to the journal's guidelines on [minimum standards of reporting?] Yes

Choose an item.

**Statistics**

Are you able to assess all statistics in the manuscript, including the appropriateness of statistical tests used? No, I do not feel adequately qualified to assess the statistics.

**Quality of Written English**

Please indicate the quality of language in the manuscript: Acceptable

**Declaration of Competing Interests**

Please complete a declaration of competing interests, considering the following questions:

- Have you in the past five years received reimbursements, fees, funding, or salary from an organisation that may in any way gain or lose financially from the publication of this manuscript, either now or in the future?
- Do you hold any stocks or shares in an organisation that may in any way gain or lose financially from the publication of this manuscript, either now or in the future?
- Do you hold or are you currently applying for any patents relating to the content of the manuscript?
- Have you received reimbursements, fees, funding, or salary from an organization that holds or has applied for patents relating to the content of the manuscript?
- Do you have any other financial competing interests?
- Do you have any non-financial competing interests in relation to this paper?

If you can answer no to all of the above, write 'I declare that I have no competing interests' below. If your reply is yes to any, please give details below.

I declare that I have no competing interests’

I agree to the open peer review policy of the journal. I understand that my name will be included on my report to the authors and, if the manuscript is accepted for publication, my named report including any attachments I upload will be posted on the website along with the authors' responses. I agree for my report to be made available under an Open Access Creative Commons CC-BY license (http://creativecommons.org/licenses/by/4.0/). I understand that any comments which I do not wish to be included in my named report can be included as confidential comments to the editors, which will not be published.

I agree to the open peer review policy of the journal

To further support our reviewers, we have joined with Publons, where you can gain additional credit to further highlight your hard work (see: https://publons.com/journal/530/gigascience). On publication of

this paper, your review will be automatically added to Publons, you can then choose whether or not to claim your Publons credit. I understand this statement.

Yes
